# Supplementary material for: Digital and online symptom checkers and health assessment/triage services for urgent health problems: systematic review
Source: BMJ Open. 2019 Aug 1;9(8):e027743. doi: 10.1136/bmjopen-2018-027743 (PMC6688675; doi:10.1136/bmjopen-2018-027743)
Supplement: Supplementary Appendix 1 [file bmjopen-2018-027743supp001.pdf]

Appendix 1: Database search strategies

Database: Ovid MEDLINE(R) Epub Ahead of Print, In-Process & Other Non-Indexed Citations, Ovid MEDLINE(R) Daily and Ovid MEDLINE(R) <1946 to Present>
Search Strategy:
----
1 (symptom checker or symptoms checker or symptom checkers or symptoms checkers).tw. (21)
2 ("self diagnosis" or "self referral" or "self triage" or "self assessment").tw. (10438)
3 TRIAGE/ (10017)
4 2 or 3 (20415)
5 (online or on-line or web or electronic or automated or internet or digital or app or mobile or smartphone).tw. (658190)
6 4 and 5 (1568)
7 ("online diagnosis" or "web based triage" or "electronic triage" or etriage).tw. (42)
8 1 or 6 or 7 (1608)

****

Embase

----
1 (symptom checker or symptoms checker or symptom checkers or symptoms checkers).tw.
2 ("self diagnosis" or "self referral" or "self triage" or "self assessment").tw.
3 emergency health service/
4 2 or 3
5 (online or on-line or web or electronic or automated or internet or digital or app or mobile or smartphone).tw.
6 4 and 5
7 ("online diagnosis" or "web based triage" or "electronic triage" or etriage).mp. [mp=title, abstract, heading word, drug trade name, original title, device manufacturer, drug manufacturer, device trade name, keyword, floating subheading word, candidate term word]
8 1 or 6 or 7

Cochrane Library

#1 symptom checker or symptoms checker or symptom checkers or symptoms checkers:ti,ab,kw (Word variations have been searched)
#2 "self diagnosis" or "self referral" or "self triage" or "self assessment":ti,ab,kw (Word variations have been searched)
#3 MeSH descriptor: [Triage] explode all trees
#4 #2 or #3
#5 online or on-line or web or electronic or automated or internet or digital or app or mobile or smartphone:ti,ab,kw (Word variations have been searched)
#6 #4 and #5
#7 "online diagnosis" or "web based triage" or "electronic triage" or etriage:ti,ab,kw (Word variations have been searched)
#8 #1 or #6 or #7

CINAHL

S8 (S1 OR S6 OR S7)
S7 TI ("online diagnosis" or "web based triage" or "electronic triage" or etriage) OR AB ("online diagnosis" or "web based triage" or "electronic triage" or etriage)
S6 S4 AND S5

S5 TI (online or on-line or web or electronic or automated or internet or digital or app or mobile or smartphone) OR AB (online or on-line or web or electronic or automated or internet or digital or app or mobile or smartphone)
S4 (S2 OR S3)
S3 (MH "Triage")
S2 TI ("self diagnosis" or "self referral" or "self triage" or "self assessment") OR AB ("self diagnosis" or "self referral" or "self triage" or "self assessment")
S1 TI (symptom checker or symptoms checker or symptom checkers or symptoms checkers) OR AB (symptom checker or symptoms checker or symptom checkers or symptoms checkers)

ACM digital library

WOS
#8 #7 OR #6 OR #1
#7 TS=("online diagnosis" OR "web based triage" OR "electronic triage" OR etriage)
#6 #5 AND #4
#5 TS=(online OR on-line OR web OR electronic OR automated OR internet OR digital OR app OR mobile OR smartphone)
#4 #3 OR #2
#3 TS=triage
#2 TS=("self diagnosis" or "self referral" or "self triage" or "self assessment")
#1 (symptom checker or symptoms checker or symptom checkers or symptoms checkers)

HMIC

1 (symptom checker OR symptoms checker OR symptom checkers OR symptoms checkers).ti,ab
2 ("self diagnosis" OR "self referral" OR "self triage" OR "self assessment").ti,ab
3 TRIAGE/
4 (2 OR 3)
5 (online OR on-line OR web OR electronic OR automated OR internet OR digital OR app OR mobile OR smartphone).ti,ab
6 (4 AND 5)
7 ("online diagnosis" OR "web based triage" OR "electronic triage" OR etriage).ti,ab
8 (1 OR 6 OR 7)
