# Supplementary material for: Digital and online symptom checkers and health assessment/triage services for urgent health problems: systematic review
Source: BMJ Open. 2019 Aug 1;9(8):e027743. doi: 10.1136/bmjopen-2018-027743 (PMC6688675; doi:10.1136/bmjopen-2018-027743)
Supplement: Supplementary Appendix 2 [file bmjopen-2018-027743supp002.pdf]

Appendix 2: Risk of bias tables

Risk of bias results for randomised trials

Short Title	Reference	Selection and performance bias	Detection and attrition bias	Reporting and other bias
Little (2016)	**Study ID**<br>• Reference<br>*Little 2016*[12]	**Random sequence generation**<br>• Low risk<br><br>**Allocation concealment**<br>• Low risk<br><br>**Blinding of participants and personnel***<br>• Unclear	**Blinding of outcome assessment***<br>• Low risk<br>*Blinded assessment of primary care records*<br><br>**Incomplete outcome data***<br>• Low risk	**Selective reporting**<br>• Unclear<br><br>**Anything else, ideally prespecified**<br>• Low risk
Yardley (2010)	**Study ID**<br>• Reference<br>*Yardley 2010*[13]	**Random sequence generation**<br>• Low risk<br><br>**Allocation concealment**<br>• Low risk<br><br>**Blinding of participants and personnel***<br>• Low risk	**Blinding of outcome assessment***<br>• Unclear<br><br>**Incomplete outcome data***<br>• Low risk	**Selective reporting**<br>• Unclear<br><br>**Anything else, ideally prespecified**<br>• Low risk

Risk of bias results for cohort/cross-sectional studies

Reference	Questions 1-4	Questions 5-7	Questions 8-10
• Reference<br>*Backman A-S et al. 2012*[30]	**1. Was the research question clearly stated?**<br>• Yes<br>*The aims refer to "non-urgent" but the information is sought prior to visiting ED.*<br><br>**2. Was the study population clearly specified and defined?**<br>• Yes<br><br>**3. Was the participation rate at least 50%?**<br>• Yes<br>*79%*<br><br>**4. Were all the subjects selected or recruited from the same or similar populations?**<br>• Yes<br>*Primary care and ED attendees*	**5. Was a sample size justification provided?**<br>• No<br><br>**6. Did the study examine exposure levels?**<br>• Yes<br>*Health advice seeking*<br><br>**7. Were exposure measures clearly defined?**<br>• Unclear<br>*Measures are vague, e.g. "previous use" of information Also, discriminating between types of information*	**8. Were outcome measures clearly defined?**<br>• Unclear<br>*"Health care information use in the past"*<br><br>**9. Were outcome assessors blinded?**<br>• Not applicable<br><br>**10. Were confounders adjusted for?**<br>• Yes<br>*To some extent: participant and physician attributes assessed for influence on the results.*
• Reference<br>*Carter 2018* [26]	**1. Was the research question clearly stated?**<br>• Yes	**5. Was a sample size justification provided?**<br>• No	**8. Were outcome measures clearly defined?**<br>• Yes<br>*Attitudes and experiences of practice staff and*

	**2. Was the study population clearly specified and defined?**<br>• Yes<br>*GPs, practice staff and their patients at 6 practices in Devon*<br><br>**3. Was the participation rate at least 50%?**<br>• No<br>*Postal survey only had response rate of 35.1% but also GPs judgement of webGP requests and 5GPs and 5 administrators were interviewed.*<br><br>**4. Were all the subjects selected or recruited from the same or similar populations?**<br>• Yes<br>*GPs, practice staff and their patients at 6 practices in Devon*	**6. Did the study examine exposure levels?**<br>• Not applicable<br><br>**7. Were exposure measures clearly defined?**<br>• Not applicable	*patients on webGP.*<br><br>**9. Were outcome assessors blinded?**<br>• Not applicable<br><br>**10. Were confounders adjusted for?**<br>• Not applicable
• Reference<br>*Cowie 2018*[27]	**1. Was the research question clearly stated?**<br>• Yes<br><br>**2. Was the study population clearly specified and defined?**<br>• Yes	**5. Was a sample size justification provided?**<br>• No<br><br>**6. Did the study examine exposure levels?**<br>• No	**8. Were outcome measures clearly defined?**<br>• Yes<br><br>**9. Were outcome assessors blinded?**<br>• No<br><br>**10. Were confounders adjusted for?**

	**3. Was the participation rate at least 50%?**<br>• No<br>*No for patient surveys*<br><br>**4. Were all the subjects selected or recruited from the same or similar populations?**<br>• Yes	**7. Were exposure measures clearly defined?**<br>• Not applicable	• Yes
• Reference *Joury et al. 2016 US*[31]	**1. Was the research question clearly stated?**<br>• Yes<br><br>**2. Was the study population clearly specified and defined?**<br>• Not applicable<br><br>**3. Was the participation rate at least 50%?**<br>• Not applicable<br><br>**4. Were all the subjects selected or recruited from the same or similar populations?**<br>• Not applicable	**5. Was a sample size justification provided?**<br>• No<br><br>**6. Did the study examine exposure levels?**<br>• Not applicable<br><br>**7. Were exposure measures clearly defined?**<br>• Not applicable	**8. Were outcome measures clearly defined?**<br>• Yes<br>*Scores used for readability, popularity, content and quality*<br><br>**9. Were outcome assessors blinded?**<br>• Not applicable<br><br>**10. Were confounders adjusted for?**<br>• Unclear
• Reference *Kellermann 2010* [11]	**1. Was the research question clearly stated?**<br>• Unclear<br><br>**2. Was the study population clearly specified and**	**5. Was a sample size justification provided?**<br>• Not applicable	**8. Were outcome measures clearly defined?**<br>• Not applicable<br><br>**9. Were outcome assessors blinded?**

	**defined?**<br>• Unclear<br>*Patients with influenza-like illness in US that accessed one of 2 websites http://www.flu.gov and www.H1N2 ResponseCenter.com*<br><br>**3. Was the participation rate at least 50%?**<br>• Not applicable<br><br>**4. Were all the subjects selected or recruited from the same or similar populations?**<br>• Unclear<br>*Only counted web hits, no demographic data available on patients. No data on usage of algorithm by clinicians or call centers.*	**6. Did the study examine exposure levels?**<br>• Not applicable<br><br>**7. Were exposure measures clearly defined?**<br>• Not applicable	• Not applicable<br><br>**10. Were confounders adjusted for?**<br>• Not applicable
• Reference<br>*Lanseng & Andreassen 2007 Norway*[32]	**1. Was the research question clearly stated?**<br>• Yes<br><br>**2. Was the study population clearly specified and defined?**<br>• Yes<br><br>**3. Was the participation rate at least 50%?**<br>• Unclear	**5. Was a sample size justification provided?**<br>• No<br><br>**6. Did the study examine exposure levels?**<br>• No<br>*Readiness*<br><br>**7. Were exposure**	**8. Were outcome measures clearly defined?**<br>• Yes<br>*Use of TRI*<br><br>**9. Were outcome assessors blinded?**<br>• No<br><br>**10. Were confounders adjusted for?**<br>• Unclear

	**4. Were all the subjects selected or recruited from the same or similar populations?**<br>• Yes	**measures clearly defined?**<br>• Not applicable	
• Reference *Luger et al. 2014*[23]	**1. Was the research question clearly stated?**<br>• Yes<br><br>**2. Was the study population clearly specified and defined?**<br>• Yes<br><br>**3. Was the participation rate at least 50%?**<br>• Unclear<br><br>**4. Were all the subjects selected or recruited from the same or similar populations?**<br>• Yes	**5. Was a sample size justification provided?**<br>• No<br><br>**6. Did the study examine exposure levels?**<br>• No<br><br>**7. Were exposure measures clearly defined?**<br>• Not applicable	**8. Were outcome measures clearly defined?**<br>• Yes<br><br>**9. Were outcome assessors blinded?**<br>• Not applicable<br><br>**10. Were confounders adjusted for?**<br>• Unclear
• Reference *Marco-Ruiz et al. 2017 Norway*[24]	**1. Was the research question clearly stated?**<br>• Yes<br><br>**2. Was the study population clearly specified and defined?**<br>• No<br><br>**3. Was the participation rate at least 50%?**	**5. Was a sample size justification provided?**<br>• No<br><br>**6. Did the study examine exposure levels?**<br>• No	**8. Were outcome measures clearly defined?**<br>• Not applicable<br><br>**9. Were outcome assessors blinded?**<br>• Not applicable<br><br>**10. Were confounders adjusted for?**<br>• Unclear

	• Yes<br>*53%*<br><br>**4. Were all the subjects selected or recruited from the same or similar populations?**<br>• Unclear	**7. Were exposure measures clearly defined?**<br>• Not applicable	
• Reference<br>*Nagykaldi 2010* [25]	**1. Was the research question clearly stated?**<br>• Yes<br><br>**2. Was the study population clearly specified and defined?**<br>• Yes<br>*Study population was patients from 12 primary care practices in US.*<br><br>**3. Was the participation rate at least 50%?**<br>• Not applicable<br><br>**4. Were all the subjects selected or recruited from the same or similar populations?**<br>• Yes<br>*All participants were patients from 12 primary care practices that accessed customised practice website or telephone helpline*	**5. Was a sample size justification provided?**<br>• Not applicable<br><br>**6. Did the study examine exposure levels?**<br>• Not applicable<br><br>**7. Were exposure measures clearly defined?**<br>• Not applicable	**8. Were outcome measures clearly defined?**<br>• Yes<br>*Web hits on customised practice website influenza self-management webpages. Downloads of self-management influeza toolkit. Completion of Iflueza self-triage module sessions. Volume of calls to telephone hotlines. Qualitative feedback from patients on statisfaction with and utility of self-management websites and telephone hotline. Qualitative feedback from clinicians around their involvement and their perceptionsof patient self-management techniques.*<br><br>**9. Were outcome assessors blinded?**<br>• Not applicable<br><br>**10. Were confounders adjusted for?**<br>• Not applicable

• Reference<br>*Nijland 2009*[29]	**1. Was the research question clearly stated?**<br>• Yes<br><br>**2. Was the study population clearly specified and defined?**<br>• Yes<br><br>**3. Was the participation rate at least 50%?**<br>• Unclear<br><br>**4. Were all the subjects selected or recruited from the same or similar populations?**<br>• Yes	**5. Was a sample size justification provided?**<br>• No<br><br>**6. Did the study examine exposure levels?**<br>• Not applicable<br><br>**7. Were exposure measures clearly defined?**<br>• Not applicable	**8. Were outcome measures clearly defined?**<br>• Yes<br><br>**9. Were outcome assessors blinded?**<br>• No<br><br>**10. Were confounders adjusted for?**<br>• Yes<br>*Methods not very clearly reported but appears to be multiple regression*
• Reference<br>*Nijland 2016*[19]	**1. Was the research question clearly stated?**<br>• Yes<br><br>**2. Was the study population clearly specified and defined?**<br>• Yes<br><br>**3. Was the participation rate at least 50%?**<br>• No<br>*Low participation rate in survey relative to users of triage system (though unclear how many were invited to participate)*	**5. Was a sample size justification provided?**<br>• No<br><br>**6. Did the study examine exposure levels?**<br>• Not applicable<br><br>**7. Were exposure measures clearly defined?**	**8. Were outcome measures clearly defined?**<br>• Yes<br><br>**9. Were outcome assessors blinded?**<br>• No<br><br>**10. Were confounders adjusted for?**<br>• Unclear

	**4. Were all the subjects selected or recruited from the same or similar populations?**<br>• Yes	• Not applicable	
• Reference<br>*North et. al. 2011*[34]	**1. Was the research question clearly stated?**<br>• Yes<br><br>**2. Was the study population clearly specified and defined?**<br>• Yes<br><br>**3. Was the participation rate at least 50%?**<br>• Not applicable<br><br>**4. Were all the subjects selected or recruited from the same or similar populations?**<br>• Not applicable	**5. Was a sample size justification provided?**<br>• Not applicable<br><br>**6. Did the study examine exposure levels?**<br>• Yes<br>*Self-exposure*<br><br>**7. Were exposure measures clearly defined?**<br>• Not applicable	**8. Were outcome measures clearly defined?**<br>• Yes<br><br>**9. Were outcome assessors blinded?**<br>• Not applicable<br><br>**10. Were confounders adjusted for?**<br>• Unclear<br>*Some discussion of potential confounders.*
• Reference<br>*Sole 2006*[18]	**1. Was the research question clearly stated?**<br>• Yes<br>*"The primary purpose of this study was to identify and describe the demographic profile of students who used the newly implemented Web-based triage system. A secondary purpose was to compare Web-based triage diagnoses to the diagnoses made in clinic for a subset*	**5. Was a sample size justification provided?**<br>• No<br><br>**6. Did the study examine exposure**	**8. Were outcome measures clearly defined?**<br>• Not applicable<br><br>**9. Were outcome assessors blinded?**<br>• Not applicable

	of students who requested appointments"<br><br>**2. Was the study population clearly specified and defined?**<br>• Yes<br>*Students who used the web based triage over a four month implementation period (1290 students). Then of those students, those who requested an appointment via email (143 students), then of those 59 who attended the health centre after requesting an email appointment.*<br><br>**3. Was the participation rate at least 50%?**<br>• Not applicable<br><br>**4. Were all the subjects selected or recruited from the same or similar populations?**<br>• Yes	**levels?**<br>• Yes<br><br>**7. Were exposure measures clearly defined?**<br>• Yes	**10. Were confounders adjusted for?**<br>• Not applicable

Risk of bias results for diagnostic studies

Reference	Questions 1 to 4	Questions 5 to 8	Questions 9 to 11
**Study ID**<br>•<br>Reference<br>*Poote*	**1. Representative spectrum?**<br>• No<br>*Study participants were all patients registered at a student health centre in England attending with new acute*	**5. Differential verification avoided?**<br>• Not applicable?	**9. Relevant clinical information?**<br>• Yes<br><br>**10. Were uninterpretable results reported?**

2014 [17]	*symptoms. If the self-assessment triage system was only for students to be representative the study population would have needed to include range of student health centres in different areas. If the system was for any UK general practices the study population would have needed to include patients of all ages, ethnicity, gender etc from a range GP practices in different areas.*<br><br>**2. Acceptable reference standard?**<br>• Yes<br><br>**3. Acceptable delay between tests?**<br>• Yes<br><br>**4. Partial verification avoided?**<br>• Yes<br>*All patients that completed self-triage also had a GP consultation where the GP rated the urgency of their consultation.*	**6. Was the reference standard independent of the index test?**<br>• Unclear<br>*Patients took the assessment from self-triage through to their GP consultation.*<br><br>**7. Index test results blinded?**<br>• No<br>*Patients took the assessment from self-triage through to their GP consultation.*<br><br>**8. Reference standard results blinded?**<br>• Yes	• Not applicable<br><br>**11. Were withdrawals from the study explained?**<br>• Yes
**Study ID**	**1. Representative spectrum?**	**5. Differential**	**9. Relevant clinical information?**

• Reference<br>*Price 2013* [20]	• No<br>*SORT was only trialled in 2 Emergency Departments in US, a larger range would be needed for a representative spectrum. Also, patients were from ED not home so potentially sicker patients in the sample.*<br><br>**2. Acceptable reference standard?**<br>• Yes<br>*Sensitivity of SORT for kids algorithm in identifying the need for ED care was based on an explicit gold standard: documented evidence that the child received 1 or more of 5 ED-specific interventions.*<br><br>**3. Acceptable delay between tests?**<br>• Yes<br><br>**4. Partial verification avoided?**<br>• Yes	**verification avoided?**<br>• Not applicable?<br><br>**6. Was the reference standard independent of the index test?**<br>• Yes<br><br>**7. Index test results blinded?**<br>• Yes<br><br>**8. Reference standard results blinded?**<br>• Yes	• Yes<br><br>**10. Were uninterpretable results reported?**<br>• Not applicable<br><br>**11. Were withdrawals from the study explained?**<br>• No
**Study ID**<br>•<br>Reference<br>*Semigran 2015* [4]	**1. Representative spectrum?**<br>• Unclear<br>*There were 45 standardised patient vignettes which were divided into three levels of triage urgency and included more and less common conditions. It is not clear how closely this replicates the spectrum of conditions that people use symptom checkers for.*	**5. Differential verification avoided?**<br>• Not applicable?<br><br>**6. Was the reference standard independent of the**	**9. Relevant clinical information?**<br>• Yes<br>*This is the clinical information that would be supplied by the patient which may or may not differ from the information given by the vignette.*<br>[#548 Semigran 2015.pdf] Page 8: *ion of the true clinical accuracy of symptom checkers.33 Some standardized patient vignettes con- tained specifc clinical language (for*

	**2. Acceptable reference standard?**<br>• Yes<br>[#548 Semigran 2015.pdf] Page 2: *The source for each vignette also provided the associated correct diagnosis.*<br><br>**3. Acceptable delay between tests?**<br>• Not applicable<br><br>**4. Partial verification avoided?**<br>• Not applicable	**index test?**<br>• Yes<br><br>**7. Index test results blinded?**<br>• Yes<br><br>**8. Reference standard results blinded?**<br>• Yes	*example, mouth ulcers, tonsils with exudate), and actual patients with the same condition might struggle with the words to use to describe their symptoms or use diferent terms. Therefore, our analysis represents an indirect assess- ment of how well symptom checkers would perform with actual patients*<br><br>**10. Were uninterpretable results reported?**<br>• Yes<br>[#548 Semigran 2015.pdf] Page 3: *ns for diagnosis and triage was high (Cohen's κ 0.90). In some cases we could not evaluate a vignette because some symptom checkers focus only on children or on adults or the symptom checker did not list or ask for the key symp- tom in the vignette. To avoid penalizing these symptom checkers, we referred to standardized patient vignettes that successfully yielded an output as "standardized patient evaluations."*<br><br>**11. Were withdrawals from the study explained?**<br>• Not applicable
**Study ID**<br>•<br>Reference *Semigran 2016* [8]	**1. Representative spectrum?**<br>• Unclear<br>*There were 45 standardised patient vignettes which were divided into three levels of triage urgency and included more and less common conditions. It is not clear how closely this replicates the spectrum of conditions that people use symptom checkers for.*	**5. Differential verification avoided?**<br>• Not applicable?<br><br>**6. Was the**	**9. Relevant clinical information?**<br>• Yes<br>*The physicians and the symptom checkers used the same vignettes*<br><br>**10. Were uninterpretable results reported?**

	**2. Acceptable reference standard?**<br>• Yes<br><br>**3. Acceptable delay between tests?**<br>• Not applicable<br><br>**4. Partial verification avoided?**<br>• No<br>*There was a total of 234 physicians involved in the study and of the 45 vignettes, each was solved by at least 20 physicians but it is not clear why they chose the specific vignettes to solve.*	**reference standard independent of the index test?**<br>• Not applicable<br><br>**7. Index test results blinded?**<br>• Yes<br><br>**8. Reference standard results blinded?**<br>• Yes	• Not applicable<br><br>**11. Were withdrawals from the study explained?**<br>• No<br>*It is unclear why the physicians chose to solve the specific vignettes*
