# Supplementary material for: Digital and online symptom checkers and health assessment/triage services for urgent health problems: systematic review
Source: BMJ Open. 2019 Aug 1;9(8):e027743. doi: 10.1136/bmjopen-2018-027743 (PMC6688675; doi:10.1136/bmjopen-2018-027743)
Supplement: Author's manuscript [file bmjopen-2018-027743.draft_revisions.pdf]

BMJ Open is committed to open peer review. As part of this commitment we make the peer review history of every article we publish publicly available.

When an article is published we post the peer reviewers' comments and the authors' responses online. We also post the versions of the paper that were used during peer review. These are the versions that the peer review comments apply to.

The versions of the paper that follow are the versions that were submitted during the peer review process. They are not the versions of record or the final published versions. They should not be cited or distributed as the published version of this manuscript.

BMJ Open is an open access journal and the full, final, typeset and author-corrected version of record of the manuscript is available on our site with no access controls, subscription charges or pay-per-view fees (http://bmjopen.bmj.com).

If you have any questions on BMJ Open's open peer review process please email info.bmjopen@bmj.com

BMJ Open

Digital and online symptom checkers and health assessment/triage services for urgent health problems: systematic review

Journal:	*BMJ Open*
Manuscript ID	bmjopen-2018-027743
Article Type:	Research
Date Submitted by the Author:	06-Nov-2018
Complete List of Authors:	Chambers, Duncan ; University of Sheffield, ScHARR;<br>Cantrell, Anna; University of Sheffield, ScHARR<br>Johnson, Maxine; University of Sheffield, ScHARR; University of Sheffield<br>Preston, Louise; University of Sheffield, ScHARR<br>Baxter, Susan; University of Sheffield, ScHARR<br>Booth, Andrew; University of Sheffield, ScHARR<br>Turner, Janette; University of Sheffield, ScHARR
Keywords:	urgent care, symptom checkers, systematic reviews

SCHOLARONE™
Manuscripts

Digital and online symptom checkers and health assessment/triage services for urgent health problems: systematic review

Duncan Chambers, Anna Cantrell, Maxine Johnson, Louise Preston, Susan K Baxter, Andrew Booth and Janette Turner

School of Health and Related Research (ScHARR), University of Sheffield, Regent Court, Sheffield S1 4DA, UK Duncan Chambers research fellow, Anna Cantrell research fellow, Maxine Johnson research fellow, Louise Preston research fellow, Susan K Baxter senior research fellow, Andrew Booth reader in evidence-based information practice, Janette Turner reader in emergency and urgent care research

*Correspondence to Duncan Chambers: d.chambers@sheffield.ac.uk

Contributor/guarantor information:

Susan K Baxter (Senior Research Fellow in Public Health): Protocol development; study selection; report writing; co-ordinated PPI meeting; Andrew Booth (Reader in Evidence-Based Information Practice): Information retrieval; study selection; report writing; Anna Cantrell (Research Associate in Health Economics and Decision Science): Information retrieval; study selection; data extraction; quality assessment; report writing; Duncan Chambers (Research Fellow in Public Health): Project co-ordination; study selection; data extraction; quality assessment; report writing; Maxine Johnson (Research Fellow in Public Health): Study selection; data extraction; quality assessment; report writing; Louise Preston (Research Fellow in Health Economics and Decision Science): Study selection; data extraction; quality assessment; report writing; Janette Turner (Reader in Emergency & Urgent Care Research): Topic expert advice: report writing. All authors commented on drafts of the protocol and report. Duncan Chambers is the guarantor for this work. The corresponding author attests that all listed authors meet authorship criteria and that no others meeting the criteria have been omitted.

For peer review only - http://bmjopen.bmj.com/site/about/guidelines.xhtml

For peer review only

Competing interests

None of the authors have any competing interests

Copyright

The Corresponding Author has the right to grant on behalf of all authors and does grant on behalf of all authors, a non-exclusive worldwide licence to the Publishers and its licensees in perpetuity, in all forms, formats and media (whether known now or created in the future), to i) publish, reproduce, distribute, display and store the Contribution, ii) translate the Contribution into other languages, create adaptations, reprints, include within collections and create summaries, extracts and/or, abstracts of the Contribution, iii) create any other derivative work(s) based on the Contribution, iv) to exploit all subsidiary rights in the Contribution, v) the inclusion of electronic links from the Contribution to third party material where-ever it may be located; and, vi) licence any third party to do any or all of the above.

For peer review only

Abstract

Objectives: In England, the NHS111 service provides assessment and triage by telephone for problems that are urgent but not classified as an emergency. A digital version of this service is currently being evaluated. We aimed to systematically review the evidence on digital and online symptom checkers and health assessment/triage services.

Design: Systematic review with narrative synthesis.

Setting: Primary care.

Participants: General population seeking information online or digitally to address an urgent health problem.

Interventions: Any online or digital service designed to assess symptoms, provide health advice and direct patients to appropriate services.

Primary and secondary outcome measures: Safety; clinical effectiveness; costs/cost-effectiveness; accuracy; impact on service use; compliance with advice received; patient/carer satisfaction; and equity and inclusion. Accuracy covered 1) ability to provide a correct diagnosis and 2) ability to distinguish between high and low acuity/urgency problems (and hence direct patients to appropriate services).

Results: We included 29 publications (27 studies). Evidence on patient safety was weak. Diagnostic accuracy varied between different systems but was generally low if health professionals' diagnoses were used as the reference standard. Algorithm-based triage tended to be more risk-averse than that of health professionals. There was very limited evidence on patients' compliance with online triage advice. Study participants generally expressed high levels of satisfaction with digital and online triage services, albeit in mainly uncontrolled studies. Younger and more highly educated people were more likely to use these services.

Conclusions: The English 'digital 111' service is being implemented against a background of uncertainty around the likely impact on important outcomes. The health system may need to respond to short-term increases (or decreases) in demand and/or shifts from one part of the system to another. The popularity of online and digital services with younger and more educated people has implications for health equity.

Registration: PROSPERO (registration number CRD42018093564)

For peer review only - http://bmjopen.bmj.com/site/about/guidelines.xhtml

Strengths and limitations of this study

- This systematic review was based on a rigorous search of the literature which maximised efficiency by combining an initial focused search with subsequent rounds of follow-up searching, including searches for named symptom checker systems.
- Our narrative synthesis approach used a mixture of description and tabulation to summarise the evidence, including overall strength of the evidence base for each of the pre-specified outcomes of interest.
- Given the decision to implement a national urgent care service based on digital symptom checkers in the NHS in England, our study highlights areas of uncertainty that will need to be resolved by research and data collection.
- The review inclusion criteria were relatively broad and findings from symptom checker systems for specific conditions may not be applicable to more general systems and vice versa.
- We have also included studies of symptom checkers as part of electronic consultation systems in general practice, which again represents a slightly different setting from a general 'digital 111' service, and this should be kept in mind when interpreting the results.

For peer review only

Introduction

Digital and online symptom checkers and assessment services are used by patients seeking guidance about health problems, including some that may require urgent action. These services generally provide people with possible alternative diagnoses based on their reported symptoms and/or suggest a course of action (e.g. self-care, make a GP appointment or go to an emergency department (ED)).

In England, the NHS111 service provides assessment and triage by telephone for problems that are urgent but not classified as emergencies. The latest data from NHS England[1] show that in September2018 there were over 1.27 million calls to NHS111, an average of 42,400 per day. Outcomes of these calls were that 13.2% had ambulances despatched; 9.5% were recommended to attend an ED; 58.7% were recommended to attend primary care; 4.8% to attend another service; and 13.8% were not recommended to attend another service (e.g. their condition was considered suitable for self-care)

NHS England is planning to introduce a digital platform to make NHS111accessible via a website or smartphone app. A beta version of the service (referred to as 'NHS111 Online') is available at https://111.nhs.uk/ (accessed 26 October 2018). The 'digital 111' service is seen as key to reducing demand for the telephone 111 service, enabling resources to be redirected to supporting 'integrated urgent and emergency care systems' as outlined in the 'NHS 5-year Forward View' and its 2017 update 'Next Steps on the NHS 5-year Forward View'[2 3].

There is an expectation that a digital 111 platform will help to manage demand and increase efficiency in the urgent and emergency care system, complementing the agenda of locally based Sustainability and Transformation Partnerships (STPs). However, there is a risk of increasing demand, duplicating healthcare contacts and providing advice that is not safe or clinically appropriate. For example, an evaluation of the NHS111 telephone service at four pilot sites and three control sites found that in its first year the service was not successful in reducing 999 emergency calls or in shifting patients from emergency to urgent care[4]. A recent study of 23 symptom checker algorithms providing diagnostic and triage advice that would form the basis of a 'digital 111' platform found deficiencies in both their diagnostic and triage capabilities[5].

For peer review only - http://bmjopen.bmj.com/site/about/guidelines.xhtml

In 2017, NHS England carried out pilot evaluations of different systems in four regions of England. The evaluations aimed to assess whether digital/online triage was acceptable to users and connected them to appropriate clinical care[6]. The full report of the evaluations was not yet published at the time of writing. The objective of this systematic review was to inform further development of the proposed digital platform by summarising and critiquing the previous research in this area, both from the UK and overseas. The overall research question was: for people seeking guidance about an urgent health problem, what is the effect of digital and online services designed to assess symptoms and signpost patients to appropriate services (compared with non-digital services or no comparator) on important clinical and health service outcomes? Outcomes include safety; clinical and cost-effectiveness; diagnostic and triage accuracy; impact on service use; patient/carer satisfaction; compliance with advice received; and outcomes related to equity and inclusion.

For peer review only

Methods

The review protocol was registered with PROSPERO (registration number CRD42018093564) and is available from the project website (https://www.journalslibrary.nihr.ac.uk/programmes/hsdr/164717/).

Literature search and screening

Initial scoping searches revealed that a highly sensitive search strategy, as typically conducted for systematic reviews, retrieved a disproportionately high number of references on GP decision-making and triage. We therefore devised a three stage retrieval strategy as an acceptable alternative to comprehensive topic-based searching. This involved:

1. Targeted searches of precise high specificity terms in seven databases (MEDLINE, EMBASE, the Cochrane Library, CINAHL, HMIC (Health Management Information Consortium), Web of Science and ACM Digital Library). These searches were not restricted by language or date. A sample search strategy is presented in Appendix 1.

2. Phrase searching for names of known symptom checkers using a list compiled from Semigran 2015 and other sources

For peer review only - http://bmjopen.bmj.com/site/about/guidelines.xhtml

3. Citation searches and reference checking of key included studies and reviews, complemented by contact with service providers (directly and via websites).

Search results were stored in a reference management system (EndNote) and imported into EPPI-Reviewer software for screening, data extraction and quality assessment. The search results were screened against the inclusion criteria by one reviewer, with a 10% sample screened by a second reviewer. Uncertainties were resolved by discussion among the review team.

Inclusion and exclusion criteria

Population: General population seeking information online or digitally to address an urgent health problem, including adults and children and issues arising from both acute and long-term chronic illness.

Intervention: Any online or digital service designed to assess symptoms, provide health advice and direct patients to appropriate services. Services that only provide health advice were excluded, as were those that offer treatment, e.g. online CBT services.

Comparator: The 'gold standard' comparator is current practice of telephone assessment (e.g. NHS111) or face to face assessment (e.g. general practice, urgent care centre or ED). However, studies with other relevant comparators (e.g. comparative performance in tests or simulations) or with no comparator were included if they addressed the research questions.

Outcomes: The main outcomes of interest were safety (e.g. any evidence of adverse events arising from following or ignoring advice from online/digital services); clinical effectiveness; costs/cost-effectiveness; accuracy; impact on service use; compliance with advice received; patient/carer satisfaction; and equity and inclusion. 'Accuracy covered 1) ability to provide a correct diagnosis and 2) ability to distinguish between high and low acuity/urgency problems (and hence direct patients to appropriate services).

Study design: We did not restrict inclusion by study design (and included relevant audits or service evaluations in addition to formal research studies) but included studies had to evaluate (quantitatively or qualitatively) some aspect of an online/digital service

Other: Studies from any developed country healthcare system were eligible for inclusion

Excluded: Purely descriptive studies, conceptual papers, projections of possible future developments and studies conducted in low or middle income countries were excluded from the review.

For peer review only - http://bmjopen.bmj.com/site/about/guidelines.xhtml

Data extraction and quality/strength of evidence assessment

We extracted and tabulated key data from the included studies, including study design, population/setting, results and key limitations. Data extraction was performed by one reviewer, with a 10% sample checked for accuracy and consistency.

To characterise the included digital and online systems as interventions, we identified studies reporting on a particular system and extracted data from all relevant studies using a modification of the TIDieR (Template for Intervention Description and Replication) checklist[7] which we designated TIDieST (Template for Intervention Description for Systems for Triage). Further details may be found in the full report (Chambers et al., in preparation).

Quality (risk of bias) assessment was undertaken for peer-reviewed full publications only (i.e. not grey literature publications or conference abstracts). Randomised controlled trials were assessed using the Cochrane Collaboration risk of bias tool. For diagnostic accuracy type studies, we used the Cochrane Collaboration version of QUADAS and for other study design we used the National Heart Lung and Blood Institute tool for observational cohort and cross-sectional studies. Quality assessment was performed by one reviewer, with a 10% sample checked for accuracy and consistency.

Assessment of the overall strength (quality and relevance) of evidence for each research question is part of the narrative synthesis. Overall strength of the evidence base for key outcomes was assessed using an adaptation of the method described by Baxter et al.[8] This involves classifying evidence as 'stronger', 'weaker', 'conflicting' or 'insufficient' based on study numbers and design. Specifically, "stronger evidence" represented generally consistent findings in multiple studies with a comparator group design or comparative diagnostic accuracy studies; "weaker evidence" represented generally consistent findings in one study with a comparator group design and several non-comparator studies or multiple non-comparator studies; "very limited evidence" represented an outcome reported by a single study; and finally, "inconsistent evidence" represented an outcome where fewer than 75% of studies agreed on the direction of effect. All studies in the review, including those that did not meet criteria for risk of bias assessment, were included in the strength of evidence assessment.

For peer review only - http://bmjopen.bmj.com/site/about/guidelines.xhtml

Evidence synthesis

We performed a narrative synthesis structured around the pre-specified research questions and outcomes. We did not perform any meta-analyses because the included studies varied widely in terms of design, methodology and outcomes.

Patient and public involvement (PPI)

The review was discussed at two meetings of an existing PPI group covering the programme from which the review was commissioned (Sheffield HS&DR Evidence Synthesis Centre). At the meetings there was discussion regarding the focus of the work, including a presentation on previous research on NHS111 telephone services to provide a context for understanding the current work. The meetings also included presentation and discussion of the findings of the review, in order to explore key messages for patients which could inform dissemination of the findings. Discussion during one meeting was structured using a SWOT (strengths, weaknesses, opportunities and threats) analysis approach, which revealed a number of potential concerns amongst patients as well as potential perceived benefits. Involvement of the advisory group was beneficial in highlighting some issues that had also emerged from the systematic review, and enabled the reviewers to structure the review findings taking this into account.

Results

Results of literature search

Twenty-seven studies (29 publications) were included in the review. Figure 1 presents the flow of studies through the selection process.

For peer review only

For peer review only - http://bmjopen.bmj.com/site/about/guidelines.xhtml

Figure 1: PRISMA flow diagram

Characteristics of included studies

Seventeen studies (Table 1) evaluated symptom checkers as a self-contained intervention, of which eight covered a limited range of symptoms, e.g. respiratory[9-11] or gastrointestinal[12 13] symptoms which we considered to be 'urgent'. The remaining studies in this group evaluated symptom checkers covering a wider range of common urgent care symptoms. Studies either evaluated a single system[14-17] or multiple systems[5 18]. We found only one study of a symptom checker specifically intended for assessment of children's symptoms, a development of the SORT (Strategy for Off-Site Rapid Triage) system for influenza-like illness[19] Two reports with some overlap of content evaluated the 'babylon check' app[14 20]

Five studies[6 21-24] evaluated symptom checkers as part of a broader self-assessment and consultation system (often referred to as electronic consultation or e-consultation). Study characteristics are summarised in Table 2. In this type of system, the role of symptom checkers is to help patients decide whether their symptoms require a consultation with a doctor or other health professional or can be dealt with by self-care. If a consultation is required, details of the symptoms and a request for an appointment or call-back can be submitted electronically. This type of study is important because it considers the service within the broader context of the urgent and emergency care system. A limitation is that some studies focused mainly on the 'downstream' elements of the pathway, e.g. consultation with GPs, and provided limited data on the symptom checker element of the system.

A final group of five studies examined patient and/or public attitudes to online self-diagnosis in the context of urgent care[25-29].See the full report for further details (Chambers et al. in preparation).

For peer review only - http://bmjopen.bmj.com/site/about/guidelines.xhtml

Table 1: Studies of symptom checkers as a self-contained intervention

Reference	Study design	System type	Comparator	Population/sample
Babylon Health 2017[20]	• Uncontrolled observational<br>*No control group but some comparison with NHS111 telephone data*	• Digital<br>*Smartphone app*	• Health professional performance on real-world data<br>• Other<br>*NHS111 data for 12 months from February 2017*	• General population<br>*Participants in the London pilot evaluation of 'digital 111' services*
Berry 2016[12]	• Simulation<br>*Evaluation of symptom checker performance on clinical vignettes*	• Online<br>*17 symptom checkers*	• None	• Specific condition(s)<br>*Gastrointestinal symptoms*
Berry 2017[30]	• Controlled observational	• Online<br>*Three online symptom checkers (WebMD, iTriage and FreeMD)*	• Health professional performance on real-world data	• Specific condition(s)<br>*Patients with a cough presenting to an internal medicine clinic*
Berry 2017[13]	• Controlled observational	• Online<br>*Three online symptom checkers (WebMD, iTriage, FreeMD)*	• Health professional performance on real-world data	• Specific condition(s)<br>*Abdominal pain*
Kellermann 2010 [9]	• Simulation<br>*The developed algorithm was tested against past patient records..*	• Online<br>*SORT was available on 2 interactive websites*	• Health professional performance on real-world data<br>*The algorithm was tested against clinicians' decision on past patient records.*	• Specific condition(s)<br>*Influenza symptoms*

For peer review only - http://bmjopen.bmj.com/site/about/guidelines.xhtml

For peer review only

Little 2016[10]	• Experimental<br>*Randomised controlled trial (RCT)*	• Online<br>*'Internet Doctor' website*	• Other<br>*Usual GP care without access to the Internet Doctor website*	• Specific condition(s)<br>*Respiratory infections and associated symptoms*
Luger et al. 2014 [31]	• Simulation<br>*Described as "human-computer interaction study" using think-aloud protocols.*	• Online<br>*Google and WebMD*	• Other<br>*Comparing two internet health tools.*	• General population<br>*Older adults (50 years or older)*
Marco-Ruiz et al. 2017 [32]	• Qualitative<br>*Qualitative element*<br>• Other<br>*1. Online evaluation by users (problem detection) 2. Think aloud technique by smaller sample of participants (usability)*	• Online<br>*Erdusyk*	• None	• General population<br>*Internet tool users*
Middleton 2016[14]	• Simulation	• Digital<br>*'babylon check' automatic triage system*	• Health professional performance on test/simulation<br>*Twelve 'clinicians' (doctors) and 17 nurses*	• General population
Nagykaldi 2010 [33]	• Uncontrolled observational	• Online<br>*Customised practice website including a bilingual influenza self-triage module, a downloadable influenza toolkit and electronic messaging capability.*	• None	• Specific condition(s)<br>*Influenza*

For peer review only - http://bmjopen.bmj.com/site/about/guidelines.xhtml

		A bilingual seasonal influenza telephone hotline was available as an alternative.		
Nijland 2016[17]	• Uncontrolled observational *Retrospective analysis of 15 months' data*	• Online *Web-based triage system (http://www.dokterdokter.nl)*	• None	• General population
Poote 2014 [15]	• Uncontrolled observational	• Online *Prototype self-assessment triage system*	• Health professional performance on real-world data *GPs triage rating was compared with rating from the self-assessment system*	• General population *Students attending a University Student Health Centre with new acute symptoms*
Price 2013 [19]	• Uncontrolled observational	• Online *A web-based decision support tool - Strategy for Off-site Rapid Triage (SORT) for Kids designed to help parents and adult caregivers decide whether a child with possible influenza symptoms needs to visit the emergency department for immediate care.*	• Health professional performance on real-world data *The sensitivity of the algorithm was compared with a gold standard - evidence form child's medical records that they received 1 or more of 5 ED-specific interventions.*	• Specific condition(s) *Influenza in children*
Semigran 2015 [5]	• Experimental *Described as an audit study*	• Multiple *23 symptom checkers were evaluated. Symptom checkers available as apps (via the App Store and Google Play) were identified through searching for "symptom checker" and "medical diagnosis" and screened the first 240 results. Symptom checkers*	• Other *Vignettes had a diagnosis and triage attached to them and these were compared against the*	• General population *Where a single class of illness was examined by the symptom checker, the symptom checker was*

For peer review only - http://bmjopen.bmj.com/site/about/guidelines.xhtml

		available online were identified through searching Google and Google Scholar for "symptom checker" and "medical diagnosis" and screened the first 300 results.	*symptom checker advice.*	*excluded from the study.*
Semigran 2016 [18]	• Experimental<br>*Comparison of physician and symptom checker diagnoses based on clinical vignettes*	• Multiple<br>*"Human Dx is a web-and app based platform"*	• Health professional performance on test/simulation<br>*Clincial vignettes - a comparison of 23 symptom checkers with physician diagnosis for 45 vignettes*	• General population<br>*Of the 45 condition vignettes - there were 15 low, 15 medium and 15 huigh acuity vignettes - there were 26 common and 19 uncommon condition vignettes*
Sole 2006 [16]	• Uncontrolled observational<br>*Descriptive comparative study*	• Online<br>*A web-based triage system (24/7 WebMed)*	• Health professional performance on real-world data<br>*Data was evaluated from students who had used the web based triage and then requested an appointment via email (so triage data was available for comparison).*	• General population
Yardley 2010[11]	• Experimental<br>*Exploratory randomised trial*	• Online<br>*'Internet Doctor' website*	• Other<br>*Self-care information provided as a static web page with no symptom checker or triage advice*	• Specific condition(s)<br>*Minor respiratory symptoms, e.g. cough, sore throat, fever, runny nose*

For peer review only - http://bmjopen.bmj.com/site/about/guidelines.xhtml

Table 2: Studies of symptom checkers as part of an electronic consultation system

Reference	Study design	System type	Comparator	Population/sample
Carter 2018 [21]	• Uncontrolled observational *Mixed-methods evaluation*	• Online *webGP(subsequently known as eConsult)*	• Other *Investigate patient experience by surveying patients who had used webGP and comparing their experience with controls (patients who had received a face-to-face consultation during the same time period) matched for age and gender*	• General population *General practices in NHS Northern, Eastern and Western Devon Clinical Commissioning Group's area*
Cowie 2018[22]	• Uncontrolled observational *6-month evaluation at 11 GP practices in Scotland*	• Online *eConsult, accessed via GP surgery websites. Service provides self-care assessment and advice, including symptom checkers; triage and signposting to alternative services; access to NHS24 (phone service); and e-consults allowing submission of details by e-mail)*	• None	• General population *Patients registered with participating GP practices*
Madan 2014[23]	• Uncontrolled observational *Report of 6-month pilot study*	• Online *webGP (subsequently known as eConsult)*	• None	• General population
NHS England[6]	• Uncontrolled observational *Analysis of data from four pilot studies together*	• Multiple *Pilots featured NHS Pathways (Web-based; West Yorkshire); Sense.ly ('voice-activated avatar'; West Midlands); Espert 24 (Web-based; Suffolk) and babylon (app; North Central*	• None *Authors stated it was not appropriate to compare pilot sites because of differences in starting date, 'footprints' covered, method of uptake*	• General population

For peer review only - http://bmjopen.bmj.com/site/about/guidelines.xhtml

	with data from other sources	*London)*	*and underlying population*	
Nijland 2009[24]	• Other<br>*Online survey*	• Online<br>*Responses of interest relate to 'indirect e-consultation' (consulting a GP via secure e-mail with intervention of a Web-based triage system)*	• None	• General population<br>*Patients with Internet access but no experience of e-consultation*

For peer review only

For peer review only - http://bmjopen.bmj.com/site/about/guidelines.xhtml

Results by outcome

Safety

None of the six included studies that reported on safety outcomes identified any problems or differences in outcomes between symptom checkers and health professionals. Most of the studies compared system performance with that of health professionals using real or simulated data. The only study with no comparison group was the 6-month pilot study of webGP[23], which reported 'no major incidents'.

Limitations of the studies included not being based on real patient data[14]; covering only a limited range of conditions[9 19]; and sampling a young healthy population (students) not representative of the general population of users of the urgent care system[15]. Studies of e-consultation systems did not generally collect data on those respondents who decided not to seek an appointment, limiting their ability to assess any impact on safety for this group. Overall, the evidence should be interpreted cautiously as indicating no evidence of a detrimental impact on safety rather than evidence of no detrimental effect.

Clinical effectiveness

Only two studies reported on clinical effectiveness outcomes, making it difficult to draw any firm conclusions. In the study by Little et al., those who used the Internet Doctor website experienced longer illness duration and more days of illness rated moderately bad or worse than the usual care group[10] The pilot study of the webGP system[23] reported that several patients received advice to seek treatment for serious symptoms that might otherwise have been ignored. However, no details or quantitative data were provided.

Costs/cost-effectiveness

Two included studies provided limited data on possible cost savings. Based on 6 months of pilot data, Madan[23]estimated savings of £11,000 annually for an average general practice (6,500 patients) compared with current practice. The report also suggested a saving to commissioners equivalent to £414,000 annually for a CCG covering 250,000 patients. These savings were specifically related to self-reported diversion of patients from GP appointments to self-care and from urgent care to e-consultation. Using similar methodology, the manufacturers of the 'babylon check' app claimed average savings of over £10/triage

For peer review only - http://bmjopen.bmj.com/site/about/guidelines.xhtml

compared with NHS111 by telephone, based on a higher proportion of patients being recommended to self-care[20].

Diagnostic accuracy

Eight studies reported at least some data on the diagnostic accuracy of symptom checkers. In spite of the diverse methods and comparisons in the included studies, almost all agreed that the diagnostic accuracy of symptom checkers was poor in absolute terms (e.g. in evaluating 'vignettes' designed to test knowledge of specific conditions, where the correct diagnosis was already known by definition) or relative to that of health professionals. In the most comprehensive evaluation, Semigran et al. evaluated 23 symptom checkers across 770 standardised patient evaluations[5]. Overall the correct diagnosis was made in 34% of cases (95% CI 31%-37%), although performance varied widely between symptom checkers, high and low acuity conditions and common and rare conditions. When the same authors compared the 23 symptom checkers with physicians using 43 vignettes, physicians were more likely to list the correct diagnosis first (out of three differential diagnoses) (72.1% vs. 34% $p<0.001$) as well as among the top three diagnoses (84.3% vs. 51.2% $p<0.001$)[18].

The only exception to the rule was an evaluation carried out at a student health centre[16]. Using data from 59 participants who used the 24/7 WebMed system and who were subsequently treated at the health centre, the study found good agreement between chief complaint, 24/7 WebMed classification and provider diagnosis (kappa values 0f 0.89 to 0.94). This study differed from the others in using data from students rather than a general population sample. In addition, the students' complaints were generally common and uncomplicated, a scenario in which symptom checkers performed relatively well in the study by Semigran et al.[18].

Accuracy of disposition (triage and signposting to appropriate services)

Six included studies reported on this outcome, all except one of which[13] evaluated a 'general purpose' symptom checker. As with diagnostic accuracy, diverse methodologies and outcome measures were used.

The results overall presented a mixed picture but most studies indicated that symptom checkers were inferior and/or more cautious in their triage advice compared with doctors or

For peer review only - http://bmjopen.bmj.com/site/about/guidelines.xhtml

other health professionals. In their review of 23 symptom checkers, Semigran et al. found that the systems provided appropriate triage advice in 57% (95% CI 52% to 61%) of cases[5]. Performance varied across the systems evaluated, correct triage ranging from 33% to 78%. The NHS England pilot evaluation of four systems[6] found that agreement with clinical experts varied from 30% to 95%, although the number of responses also varied, reducing the comparability of the results.

For abdominal pain, Berry et al. evaluated three symptom checkers and found that 33% of diagnoses were at the same level of urgency as physician diagnoses (emergency, non-emergency or self-care); 39% were diagnosed as more serious and 30% less serious than the physician's judgement[13]. A similar level of agreement between algorithm and clinician (39%) was reported by Poote et al.[15], while the system evaluated by Nijland et al. advised patients to visit a doctor in 85% of cases, even when the symptoms were appropriate for self-care[17].

The only studies to report clearly equal or superior accuracy of disposition using an automated system were the evaluations of Babylon check by the company that developed the system. Middleton et al.[14] reported that using patient vignettes, the app gave an accurate triage outcome in 88.2% of cases, compared with 75.5% for doctors and 73.5% for nurses. When vignettes were delivered by a medical professional rather than actors, the accuracy of Babylon check increased to over 90%. A later report looked at triage results obtained as part of the NHS England pilot evaluation, concluding that all of 74 referrals to urgent or emergency care were appropriate[20].

Impact on service use/diversion

Eight studies reported on this outcome, although one of them[9] merely stated that it was not possible to assess the effect of the intervention (a web-based influenza triage system) on patients' use of health services.

The pilot evaluation of the webGP system reported that 18% of users planned to book an appointment but chose not to do so[23] In addition, 14% of users reported that they would have attended a walk-in centre or other urgent care service if they had not had access to the webGP system.

For peer review only - http://bmjopen.bmj.com/site/about/guidelines.xhtml

The NHS England pilot evaluation of four online/digital systems in different regions of England[6] compared the recommendations of the digital systems with those of the NHS111 telephone service over a similar time period (the first months of 2017). Compared with the telephone service, the online and digital services directed a slightly higher proportion of patients to self-care (18% vs. 14%) and a lower proportion to other primary care services such as GPs, dental and pharmacy (40 vs. 60%). The manufacturer's data on the 'babylon check' app collected as part of the NHS England evaluation indicated that patients were more likely to be triaged to self-care by the app compared with NHS111 by telephone (40 vs. 14%)[20]. This figure includes people who received information leaflets on self-care as well as those who were actively triaged. If the former group is excluded, the figures for the two services are similar (14% for NHS111 and 15.6% for 'babylon check'[20].

In their study of self-assessment for students attending a university health centre, Poote et al. found that the prototype system they studied was able to identify a proportion of cases that doctors considered appropriate for self-care, suggesting a potential to reduce service use[15]. Similarly, Little et al's RCT of a web-based symptom checker designed to support self-care for respiratory symptoms[10] reported that patients in the intervention group had fewer contacts with doctors than the usual care control group despite having a longer duration of illness and more days with relatively severe symptoms. This was balanced by an increase in contacts with the NHS Direct telephone service (which preceded NHS 111) and it should be noted that the system under evaluation recommended people needing treatment to contact NHS Direct rather than go directly to a doctor. Finally, a study of young adults (students) found that intention to seek treatment for a hypothetical illness was stronger when the diagnosis was made with the aid of WebMD or Google than with no electronic aid[28].

Patient compliance with triage advice

Only two of the included studies reported specifically on patients' compliance (or intention to comply) with advice received. The NHS England pilot evaluation in four regions asked participants in two of those regions (Suffolk and London) what they intended to do based on the advice received[6]. It appears that the question was asked when patients were aware of the advice from the system but it was unclear whether the evaluation covered real or hypothetical cases. No quantitative data were provided but the report stated that in the Suffolk pilot,

For peer review only - http://bmjopen.bmj.com/site/about/guidelines.xhtml

'overall users would have followed the advice given'. However, those who were recommended to call 999 or attend an ED were more likely to seek advice from primary care or self-management. Similarly, in the London region there was generally good agreement between advice and intended action but patients recommended to call 999 or go to an ED indicated that they would seek advice from a GP. In a study of a web-based triage system in the Netherlands, 192 patients were asked about their intention to comply immediately after receiving advice from the system[17]. Thirty-five patients responded to a follow-up survey on actual compliance, of whom 20 (57%) reported that they had followed the advice. Compliance was correlated with intention to comply, which in turn was correlated with the patient's attitude towards the advice received.

Equity and inclusion

Fourteen studies investigated the outcome of equity and inclusion or compared users and non-users. One study [10]reported that patients who were classed as less deprived were more likely to agree to use "Internet Doctor" than decline participation, although no relationship was found between deprivation and results in this study or between e-Consult use and deprivation in another study [22]. Association between e-consultation use and education levels was explored in a third study. Patients with low to medium levels of education tended to be motivated toward indirect e-consultation (which involves contact with a health professional via e-mail), mainly to reduce uncertainty [24]

Evidence from included studies suggests that users of e-consultation were more likely to be young[6 21-23], employed[17 21 23] and female[6 17 22 23]than non-users. One study also found a significantly larger use by white patients (78%) than other ethnicities[22].

Risk of bias assessment

We assessed risk of bias in the two included RCTs[10 11] using the Cochrane risk of bias tool. Thirteen studies[9 16 17 21 22 24-27 29 31-33] were assessed with the tool for cross-sectional and cohort studies and four (six publications[5 15 18 19 34 35]) with the modified QUADAS tool. Seven grey literature reports and conference abstracts were not formally assessed for risk of bias[6 12-14 23 28 30]. Identified limitations were extracted for all included studies.

For peer review only - http://bmjopen.bmj.com/site/about/guidelines.xhtml

Risk of bias results are presented in Appendix 2. With the possible exception of the two randomised trials, the included studies generally had at least a moderate risk of bias. However, the diverse designs and objectives of the studies made risk of bias difficult to assess in some cases with the available tools. Grey literature reports containing relevant data were included in the review but not formally assessed for risk of bias. Reports prepared by individuals with a commercial interest in a specific system and published without independent peer review[14 23] should be treated with particular caution because of possible conflicts of interest.

Overall strength of evidence assessment/evidence map

The overall strength of evidence for key outcomes is summarised in Table 3. We found relatively strong evidence that the diagnostic accuracy of digital and online symptom checkers tends to be lower than that of health professionals; and that patients who have used these systems generally show high levels of satisfaction (mainly in non-comparative studies). Areas where evidence is lacking or inconsistent include clinical and cost-effectiveness, accuracy of disposition to appropriate services and patient compliance with advice received. For safety, we found no evidence of an increased risk with digital/online systems but the available evidence was weak.

For peer review only - http://bmjopen.bmj.com/site/about/guidelines.xhtml

Table 3: Overall strength of evidence by outcome

Outcome	Relevant studies	Evidence statement	Strength of evidence	Comments
Safety	**=Kellermann 2010**[9]<br>**=Little 2016**[10]<br>**=Middleton 2016**[14]<br>**=Poote 2014**[15]<br>**=Price 2013**[19]<br>Madan 2014[23]	No evidence of a difference in risk between health professionals and symptom checkers	Weaker	Rating changed from stronger based on study numbers and design to weaker because of low numbers of adverse events reported
Clinical effectiveness	**-Little 2016**[10]<br>?Madan 2014[23]	Insufficient evidence to draw any firm conclusions	Very limited	
Costs/cost-effectiveness	+Babylon Health 2017[20]<br>+/-Cowie 2018[22]<br>+Madan 2014[23]	Insufficient evidence to draw any firm conclusions	Inconsistent	
Diagnostic accuracy	?Berry 2016[12]<br>**-Berry 2017**[30]<br>**-Berry 2017**[13]<br>**-Price 2013**[19]<br>?Semigran 2015[5]<br>**-Semigran 2016**[18]<br>**=Sole 2006**[16]	Symptom checkers appear inferior to health professionals in terms of diagnostic accuracy	Stronger	Mainly for specific conditions or pre-prepared vignettes
Disposition accuracy	=Babylon Health 2017[20]<br>**-Berry 2017**[13]<br>=**Middleton 2016**[14]<br>?Nijland 2010[17]<br>**-Poote 2014**[15]<br>+/-Semigran 2015[5]	Inconsistent findings on accuracy of disposition	Inconsistent	Performance variable between different systems

For peer review only

Outcome	Relevant studies	Evidence statement	Strength of evidence	Comments
	+/-**NHS England 2017**[6]			
Service use/diversion	**?Kellermann 2010**[9]<br>**+/-Little 2016**[10]<br>+/-**Poote 2014**[15]<br>?**Carter 2018**[21]<br>?Cowie 2018[22]<br>+Madan 2014[23]<br>+/- **NHS England 2017**[6]<br>+Babylon Health 2017[20]<br>-Luger 2011[31]	Inconsistent findings on effects on service use	Inconsistent	
Compliance	?Nijland[17]<br>?NHS England 2017[6]	No comparative data on compliance	Very limited	
Patient/carer satisfaction	?Nagykaldi 2010[33]<br>?Nijland[17]<br>?Price 2013[19]<br>+Yardley[11]<br>?Carter 2018[21]<br>?Cowie 2018[22]<br>?Madan 2014[23]<br>?NHS England 2017[6]<br>?Lanseng 2007[27]	Most studies report high rates of patient satisfaction with symptom checkers and e-consultation systems generally	Weaker	Few studies with comparator data

Controlled studies in bold; = means no significant difference in outcomes; + means better outcome with symptom checker; +/- varying results within study; ? results difficult to interpret in comparative terms

For peer review only

Discussion

Main findings

The literature search identified 29 publications describing 27 studies that met the inclusion criteria. The overall strength of the evidence base varied between outcomes (Table 3), but in absolute terms the evidence is weak, being based largely on observational studies. A substantial component of grey literature of uncertain quality complicates the interpretation of the evidence. Interpretation of the evidence should also take into account risks of bias in individual studies.

We found little evidence to indicate whether or not digital and online symptom checkers are detrimental to patient safety. The studies that reported on the outcome were mostly short-term and involved relatively small samples and hence reported few or no adverse events. Some were limited to people with specific types of symptoms and others recruited from specific population groups not representative of typical users of urgent care services. This body of evidence should therefore be interpreted cautiously and not extrapolated to the possible impact of a nationally available digital urgent care service being used by millions of people annually.

The evidence on patient satisfaction with digital and online systems also had some limitations but these findings appear more likely to be generalisable. Study participants generally expressed high levels of satisfaction, albeit in uncontrolled studies. For example, in the NHS England pilot evaluation, 70–80% of users were satisfied with their experience at each of the pilot sites[6]. This evidence, together with the increasing reliance on digital technology in all areas of life, suggests that any national digital urgent care service may be popular and well-used.

Digital and online systems have yet to achieve a high level of accuracy in the diagnosis of specific conditions. This finding applies both to 'general purpose' symptom checkers and to those limited to particular conditions. Although the evidence was classified as relatively strong, several caveats should be applied. Some of the included studies did not recruit representative populations and others were based on standardised vignettes rather than real-

For peer review only - http://bmjopen.bmj.com/site/about/guidelines.xhtml

world data. In addition, studies that compared symptom checkers with health professionals tended to use the doctors' clinical diagnosis as the reference standard, which would bias the comparison in favour of the health professionals.

Accuracy of signposting of patients to the most appropriate level of service is closely related to diagnostic accuracy, but results for this outcome were inconsistent between studies. In general, algorithm-based triage tended to be more risk-averse than that of health professionals, with 85% of respondents being advised to visit their doctor in one study[17]. While there is considerable uncertainty about the magnitude of the effect, a national digital urgent care service could result in considerable numbers of patients receiving inappropriate advice to visit the ED or request an urgent GP appointment. Middleton and colleagues[14] claimed that the 'babylon check' app had a high degree of triage accuracy for vignettes compared with health professionals, but this non-peer-reviewed report requires further validation.

We also found inconsistent evidence on effects on service use. There was some indication that symptom checkers can influence the pattern of service use but the magnitude and direction of the effect varied between studies. Patients' reactions to online triage advice and whether they follow the advice or seek further help or information would have implications for service use but we found limited evidence for this outcome. Preliminary findings from the NHS England evaluation suggest that patients may be more likely to seek further advice for more urgent conditions[6] but further confirmation is required.

Over half of the included studies considered equity and inclusion issues either directly or by comparing users and non-users of digital triage systems. Not surprisingly, studies revealed a clear consensus that younger and more highly educated people are more likely to use these services while older and less educated patients are more likely to prefer telephone or face-to-face contact. This could have implications for health equity if urgent care pathways prioritise (or appear to prioritise) requests originating from digital sources. Problems have arisen in primary care because patients using e-consultation systems to request an appointment following online triage may be seen more quickly than those contacting the practice by telephone.

For peer review only - http://bmjopen.bmj.com/site/about/guidelines.xhtml

Strengths and limitations

This systematic review was undertaken on a short timescale using a relatively large team of experienced researchers, including both methodological and topic experts. We performed a rigorous search of the literature including reference checking and citation searching. Rather than a conventional highly sensitive search (which would have resulted in inefficiencies in the screening process), we combined an initial focused search with subsequent rounds of follow-up searching, including searches for named symptom checker systems. We assessed risk of bias in individual studies using a variety of appropriate checklists as well as summarising the overall strength of evidence for key outcomes (Table 3).

The heterogeneous and descriptive nature of the included studies meant that meta-analysis was not feasible for any of the outcomes of interest. Our narrative synthesis approach used a mixture of description and tabulation to summarise the evidence for each of the pre-specified outcomes of interest. This was a review of published (including non-peer-reviewed) literature and the coverage of systems is not exhaustive; for example, we did not extract data from websites. We also did not carry out any original analyses of raw data even where such data were available. The timing of the review meant that final results of NHS England's pilot evaluation were not available to us. We were able to make use of a draft report that was published online[6] but we acknowledge that the findings of the final evaluation report, when available, will supersede those of the 2017 draft.

The review inclusion criteria were relatively broad and findings from symptom checker systems for specific conditions may not be applicable to more general systems and vice versa. We have also included studies of symptom checkers as part of electronic consultation systems in general practice, which again represents a slightly different setting from a general 'digital 111' service, and this should be kept in mind when interpreting the results.

Implications for service delivery and research

The implications of this systematic review for service delivery should be considered in the context that a decision has already been taken to introduce a 'digital 111' service and implementation of the service is in progress. Achieving a high level of diagnostic accuracy will be key to the success of a 'digital 111' service. Failure to provide an accurate diagnosis

For peer review only - http://bmjopen.bmj.com/site/about/guidelines.xhtml

may result in outcomes including patient dissatisfaction and unwillingness to use the service again; increased use of other urgent and emergency care services; and possible risks to patient safety (although the cautious approach characteristic of most existing systems may help to mitigate this).

The studies included in the review suggest a high level of uncertainty about the impact of 'digital 111' on the urgent care system and the wider healthcare system. Some of these uncertainties can be addressed by research and data collection but the health service may need to respond to short-term increases (or decreases) in demand and/or shifts from one part of the system to another. This may increase pressure on the system, at least in the short-term. In the longer-term, if usage of the 111 telephone service decreases as planned, there may be opportunities to reconfigure the workforce to support the integrated urgent care agenda.

Based on the areas of limited evidence identified by the review, priorities for research (in addition to ongoing collection of data to monitor usage and safety of the 'digital 111' service) include studies to compare the performance of different systems directly; rigorous economic evaluations based on real-world data; research to investigate the pathways followed by patients using the service; evaluation of systems designed for childhood illnesses; and investigation of the possible role of behaviour change theory in the development and implementation of symptom checkers.

Ethical approval

Ethical approval was not required for this work

Funding

This report presents independent research funded by the National Institute for Health Research (NIHR) Health Services & Delivery Research Programme (project number HSDR16/47/17). The funding programme approved the review protocol but had no role in the collection, analysis and interpretation of the data, the writing of this paper or the decision to submit the paper for publication. The views and opinions expressed are those of the authors and do not necessarily reflect those of the NHS, the NIHR, NETSCC, the HS&DR programme or the Department of Health.

For peer review only - http://bmjopen.bmj.com/site/about/guidelines.xhtml

Data sharing

No new data have been created in the preparation of this report and therefore there is nothing available for access and further sharing. All queries should be submitted to the corresponding author.

References

1. NHS England. NHS 111 minimum data set 2018-19 2018 [Available from: https://www.england.nhs.uk/statistics/statistical-work-areas/nhs-111-minimum-data-set/statistical-work-areas-nhs-111-minimum-data-set-nhs-111-minimum-data-set-2018-19/ accessed 29 October 2018.
2. NHS England. Five year forward view. Leeds: NHS England, 2014.
3. NHS England. Next steps on the NHS Five Year Forward View. Leeds: NHS England, 2017.
4. Turner J, O'Cathain A, Knowles E, et al. Impact of the urgent care telephone service NHS 111 pilot sites: a controlled before and after study. *BMJ Open* 2013;3(11):e003451. doi: 10.1136/bmjopen-2013-003451
5. Semigran HL, Linder JA, Gidengil C, et al. Evaluation of symptom checkers for self diagnosis and triage: audit study. *BMJ* 2015;351:h3480.
6. NHS England. NHS111 online evaluation. Leeds: NHS England, 2017.
7. Hoffmann TC, Glasziou PP, Boutron I, et al. Better reporting of interventions: template for intervention description and replication (TIDieR) checklist and guide. *BMJ* 2014;348:g1687. doi: 10.1136/bmj.g1687
8. Baxter S, Johnson M, Chambers D, et al. The effects of integrated care: a systematic review of UK and international evidence. *BMC Health Serv Res* 2018;18(1):350. doi: 10.1186/s12913-018-3161-3
9. Kellermann AL, Isakov AP, Parker R, et al. Web-based self-triage of influenza-like illness during the 2009 H1N1 influenza pandemic. *Annals of Emergency Medicine* 2010;56(3):288-94.e6.
10. Little P, Stuart B, Andreou P, et al. Primary care randomised controlled trial of a tailored interactive website for the self-management of respiratory infections (Internet Doctor).[Erratum appears in BMJ Open. 2017 Mar 21;7(3):e009769corr1; PMID: 28325861]. *BMJ Open* 2016;6(4):e009769.
11. Yardley L, Joseph J, Michie S, et al. Evaluation of a Web-based intervention providing tailored advice for self-management of minor respiratory symptoms: exploratory randomized controlled trial. *Journal of Medical Internet Research* 2010;12(4):e66.
12. Berry AC, Berry BB, Nakshabendi R, et al. Evaluation of Accuracy Between Online Symptom Checkers for Diagnosis of Gastrointestinal Symptoms from MKSAP Clinical Vignette Board Review Questions. *Gastroenterology* 2016;150(4):S849-S50. doi: 10.1016/s0016-5085(16)32869-4
13. Berry AC, Cash BD, Mulekar MS, et al. Symptom checkers vs. Doctors, the ultimate test: a prospective study of patients presenting with abdominal pain. *Gastroenterology* 2017;152(5):S852-S53. doi: 10.1016/s0016-5085(17)32937-2
14. Middleton K, Butt M, Hammerla N, et al. Sorting out symptoms: design and evaluation of the 'babylon check' automated triage system. London: Babylon Health, 2016.
15. Poote AE, French DP, Dale J, et al. A study of automated self-assessment in a primary care student health centre setting. *Journal of Telemedicine & Telecare* 2014;20(3):123-7.
16. Sole ML, Stuart PL, Deichen M. Web-based triage in a college health setting. *Journal of American College Health* 2006;54(5):289-94.

For peer review only - http://bmjopen.bmj.com/site/about/guidelines.xhtml

17. Nijland N, Cranen K, Boer H, et al. Patient use and compliance with medical advice delivered by a web-based triage system in primary care. *Journal of Telemedicine and Telecare* 2010;16(1):8-11. doi: 10.1258/jtt.2009.001004
18. Semigran HL, Levine DM, Nundy S, et al. Comparison of Physician and Computer Diagnostic Accuracy. *JAMA Intern Med* 2016;176(12):1860-61. doi: 10.1001/jamainternmed.2016.6001
19. Price RA, Fagbuyi D, Harris R, et al. Feasibility of web-based self-triage by parents of children with influenza-like illness: A cautionary tale. *JAMA Pediatrics* 2013;167(2):112-18.
20. Babylon Health. NHS111 powered by babylon: outcomes evaluation. London: Babylon Health, 2017.
21. Carter M, Fletcher E, Sansom A, et al. Feasibility, acceptability and effectiveness of an online alternative to face-to-face consultation in general practice: a mixed-methods study of webGP in six Devon practices. *BMJ Open* 2018;8(2):e018688.
22. Cowie J, Calveley E, Bowers G, et al. Evaluation of a digital consultation and self-care advice tool in primary care: a multi-methods study. *International Journal of Environmental Research and Public Health* 2018;15(896)
23. Madan A. WebGP: the Virtual general practice. London: Hurley Group, 2014.
24. Nijland N, van Gemert-Pijnen J, Boer H, et al. Increasing the use of e-consultation in primary care: Results of an online survey among non-users of e-consultation. *International Journal of Medical Informatics* 2009;78(10):688-703. doi: 10.1016/j.ijmedinf.2009.06.002
25. Backman AS, Lagerlund M, Svensson T, et al. Use of healthcare information and advice among non-urgent patients visiting emergency department or primary care. *Emergency Medicine Journal* 2012;29(12):1004-06.
26. Joury AU, Alshathri M, Alkhunaizi M, et al. Internet Websites for Chest Pain Symptoms Demonstrate Highly Variable Content and Quality. *Academic Emergency Medicine* 2016;23(10):1146-52.
27. Lanseng EJ, Andreassen TW. Electronic healthcare: a study of people's readiness and attitude toward performing self-diagnosis. *International Journal of Service Industry Management* 2007;18(3-4):394-417. doi: 10.1108/09564230710778155
28. Luger TM, Suls J. Online health information and intentions to seek healthcare. *Psychosomatic Medicine* 2011;73 (3):A59.
29. North F, Varkey P, Laing B, et al. Are e-health web users looking for different symptom information than callers to triage centers? *Telemedicine Journal & E-Health* 2011;17(1):19-24.
30. Berry AC, Berry NA, Wang B, et al. Symptom checkers versus doctors: A prospective, head-to-head comparison for GERD vs. Non-GERD Cough. *American Journal of Gastroenterology* 2017;112 (Supplement 1):S190. doi: http://dx.doi.org/10.1038/ajg.2017.299
31. Luger TM, Houston TK, Suls J. Older adult experience of online diagnosis: results from a scenario-based think-aloud protocol. *Journal of Medical Internet Research* 2014;16(1):e16.
32. Marco-Ruiz L, Bones E, de la Asuncion E, et al. Combining multivariate statistics and the think-aloud protocol to assess Human-Computer Interaction barriers in symptom checkers. *Journal of Biomedical Informatics* 2017;74:104-22.
33. Nagykaldi Z, Calmbach W, Dealleaume L, et al. Facilitating patient self-management through telephony and web technologies in seasonal influenza. *Informatics in Primary Care* 2010;18(1):9-16.
34. Fraser HSF, Clamp S, Wilson CJ. Limitations of Study on Symptom Checkers. *JAMA Internal Medicine* 2017;177(5):740-41.
35. Mehrotra A, Semigran HL, Levine DM, et al. Limitations of Study on Symptom Checkers. *JAMA Internal Medicine* 2017;177(5):741-41.

.

For peer review only - http://bmjopen.bmj.com/site/about/guidelines.xhtml

For peer review only

For peer review only - http://bmjopen.bmj.com/site/about/guidelines.xhtml

PRISMA 2009 Flow Diagram

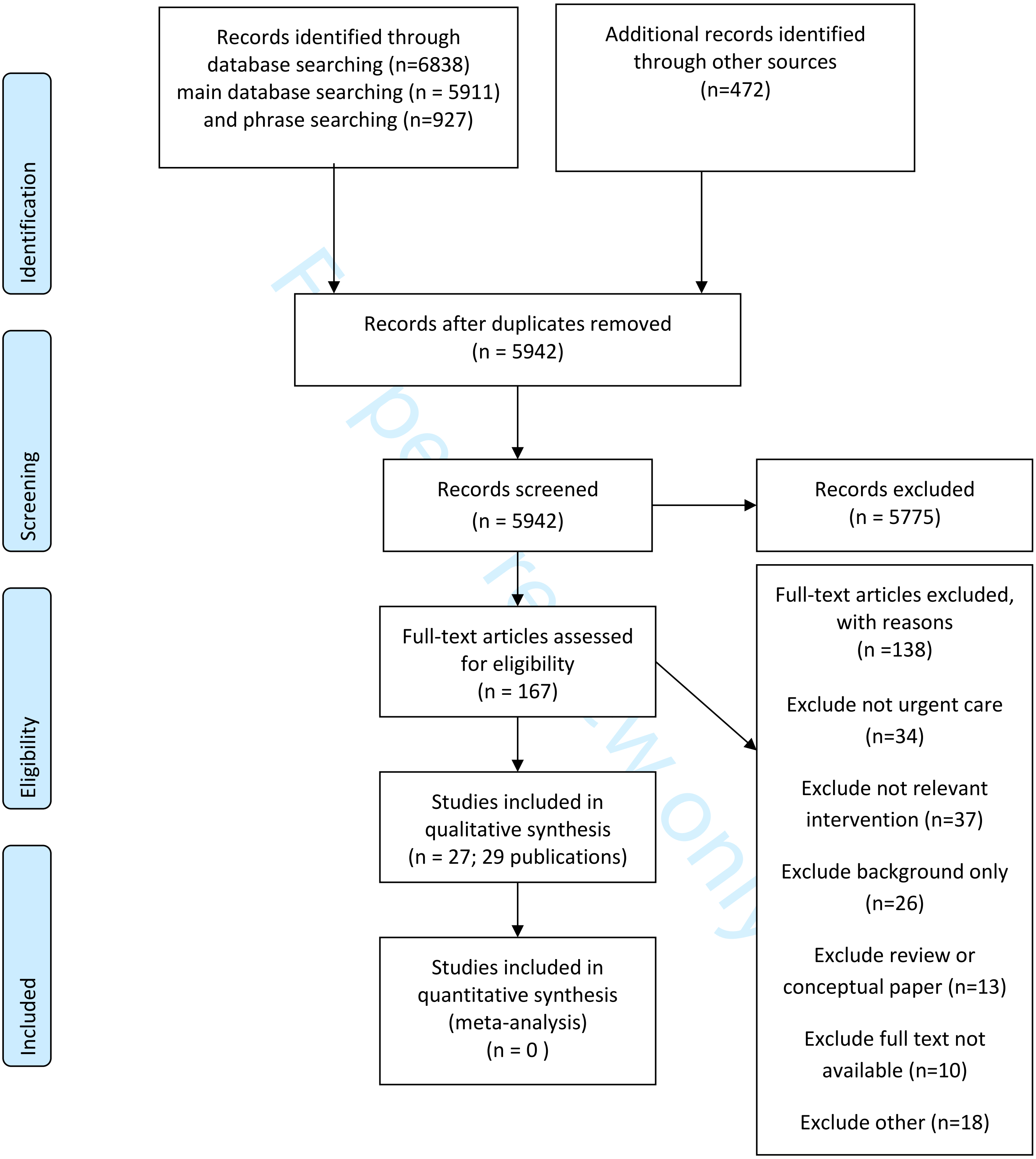

For peer review only

For peer review only

Appendix 1: Highly focused specific MEDLINE search strategy (adapted for other databases)

Database: Ovid MEDLINE(R) Epub Ahead of Print, In-Process & Other Non-Indexed Citations, Ovid MEDLINE(R) Daily and Ovid MEDLINE(R) <1946 to Present>
Search Strategy:
----

1. (symptom checker or symptoms checker or symptom checkers or symptoms checkers).tw.
2. ("self diagnosis" or "self referral" or "self triage" or "self assessment").tw. (10403)
3. TRIAGE/
4. 2 or 3
5. (online or on-line or web or electronic or automated or internet or digital or app or mobile or smartphone).tw.
6. 4 and 5
7. ("online diagnosis" or "web based triage" or "electronic triage" or etriage).tw.
8. 1 or 6 or 7

For peer review only

Appendix 2: Risk of bias tables

Risk of bias results for randomised trials

Short Title	Reference	Selection and performance bias	Detection and attrition bias	Reporting and other bias
Little (2016)	**Study ID**<br>• Reference<br>*Little 2016*[12]	**Random sequence generation**<br>• Low risk<br><br>**Allocation concealment**<br>• Low risk<br><br>**Blinding of participants and personnel***<br>• Unclear	**Blinding of outcome assessment***<br>• Low risk<br>*Blinded assessment of primary care records*<br><br>**Incomplete outcome data***<br>• Low risk	**Selective reporting**<br>• Unclear<br><br>**Anything else, ideally prespecified**<br>• Low risk
Yardley (2010)	**Study ID**<br>• Reference<br>*Yardley 2010*[13]	**Random sequence generation**<br>• Low risk<br><br>**Allocation concealment**<br>• Low risk<br><br>**Blinding of participants and personnel***<br>• Low risk	**Blinding of outcome assessment***<br>• Unclear<br><br>**Incomplete outcome data***<br>• Low risk	**Selective reporting**<br>• Unclear<br><br>**Anything else, ideally prespecified**<br>• Low risk

For peer review only

Risk of bias results for cohort/cross-sectional studies

**Reference**	**Questions 1-4**	**Questions 5-7**	**Questions 8-10**
• Reference<br>*Backman A-S et al. 2012*[30]	**1. Was the research question clearly stated?**<br>• Yes<br>*The aims refer to "non-urgent" but the information is sought prior to visiting ED.*<br><br>**2. Was the study population clearly specified and defined?**<br>• Yes<br><br>**3. Was the participation rate at least 50%?**<br>• Yes<br>*79%*<br><br>**4. Were all the subjects selected or recruited from the same or similar populations?**<br>• Yes<br>*Primary care and ED attendees*	**5. Was a sample size justification provided?**<br>• No<br><br>**6. Did the study examine exposure levels?**<br>• Yes<br>*Health advice seeking*<br><br>**7. Were exposure measures clearly defined?**<br>• Unclear<br>*Measures are vague, e.g. "previous use" of information Also, discriminating between types of information*	**8. Were outcome measures clearly defined?**<br>• Unclear<br>*"Health care information use in the past"*<br><br>**9. Were outcome assessors blinded?**<br>• Not applicable<br><br>**10. Were confounders adjusted for?**<br>• Yes<br>*To some extent: participant and physician attributes assessed for influence on the results.*
• Reference<br>*Carter 2018* [26]	**1. Was the research question clearly stated?**<br>• Yes	**5. Was a sample size justification provided?**<br>• No	**8. Were outcome measures clearly defined?**<br>• Yes<br>*Attitudes and experiences of practice staff and*

For peer review only

	**2. Was the study population clearly specified and defined?**<br>• Yes<br>*GPs, practice staff and their patients at 6 practices in Devon*<br><br>**3. Was the participation rate at least 50%?**<br>• No<br>*Postal survey only had response rate of 35.1% but also GPs judgement of webGP requests and 5GPs and 5 administrators were interviewed.*<br><br>**4. Were all the subjects selected or recruited from the same or similar populations?**<br>• Yes<br>*GPs, practice staff and their patients at 6 practices in Devon*	**6. Did the study examine exposure levels?**<br>• Not applicable<br><br>**7. Were exposure measures clearly defined?**<br>• Not applicable	*patients on webGP.*<br><br>**9. Were outcome assessors blinded?**<br>• Not applicable<br><br>**10. Were confounders adjusted for?**<br>• Not applicable
• Reference *Cowie 2018*[27]	**1. Was the research question clearly stated?**<br>• Yes<br><br>**2. Was the study population clearly specified and defined?**<br>• Yes	**5. Was a sample size justification provided?**<br>• No<br><br>**6. Did the study examine exposure levels?**<br>• No	**8. Were outcome measures clearly defined?**<br>• Yes<br><br>**9. Were outcome assessors blinded?**<br>• No<br><br>**10. Were confounders adjusted for?**

For peer review only

	**3. Was the participation rate at least 50%?**<br>• No<br>*No for patient surveys*<br><br>**4. Were all the subjects selected or recruited from the same or similar populations?**<br>• Yes	**7. Were exposure measures clearly defined?**<br>• Not applicable	• Yes
• Reference *Joury et al. 2016 US*[31]	**1. Was the research question clearly stated?**<br>• Yes<br><br>**2. Was the study population clearly specified and defined?**<br>• Not applicable<br><br>**3. Was the participation rate at least 50%?**<br>• Not applicable<br><br>**4. Were all the subjects selected or recruited from the same or similar populations?**<br>• Not applicable	**5. Was a sample size justification provided?**<br>• No<br><br>**6. Did the study examine exposure levels?**<br>• Not applicable<br><br>**7. Were exposure measures clearly defined?**<br>• Not applicable	**8. Were outcome measures clearly defined?**<br>• Yes<br>*Scores used for readability, popularity, content and quality*<br><br>**9. Were outcome assessors blinded?**<br>• Not applicable<br><br>**10. Were confounders adjusted for?**<br>• Unclear
• Reference *Kellermann 2010* [11]	**1. Was the research question clearly stated?**<br>• Unclear<br><br>**2. Was the study population clearly specified and**	**5. Was a sample size justification provided?**<br>• Not applicable	**8. Were outcome measures clearly defined?**<br>• Not applicable<br><br>**9. Were outcome assessors blinded?**

For peer review only

	**defined?**<br>• Unclear<br>*Patients with influenza-like illness in US that accessed one of 2 websites http://www.flu.gov and www.H1N2 ResponseCenter.com*<br><br>**3. Was the participation rate at least 50%?**<br>• Not applicable<br><br>**4. Were all the subjects selected or recruited from the same or similar populations?**<br>• Unclear<br>*Only counted web hits, no demographic data available on patients. No data on usage of algorithm by clinicians or call centers.*	**6. Did the study examine exposure levels?**<br>• Not applicable<br><br>**7. Were exposure measures clearly defined?**<br>• Not applicable	• Not applicable<br><br>**10. Were confounders adjusted for?**<br>• Not applicable
• Reference<br>*Lanseng & Andreassen 2007 Norway*[32]	**1. Was the research question clearly stated?**<br>• Yes<br><br>**2. Was the study population clearly specified and defined?**<br>• Yes<br><br>**3. Was the participation rate at least 50%?**<br>• Unclear	**5. Was a sample size justification provided?**<br>• No<br><br>**6. Did the study examine exposure levels?**<br>• No<br>*Readiness*<br><br>**7. Were exposure**	**8. Were outcome measures clearly defined?**<br>• Yes<br>*Use of TRI*<br><br>**9. Were outcome assessors blinded?**<br>• No<br><br>**10. Were confounders adjusted for?**<br>• Unclear

For peer review only

	**4. Were all the subjects selected or recruited from the same or similar populations?**<br>• Yes	**measures clearly defined?**<br>• Not applicable	
• Reference *Luger et al. 2014*[23]	**1. Was the research question clearly stated?**<br>• Yes<br><br>**2. Was the study population clearly specified and defined?**<br>• Yes<br><br>**3. Was the participation rate at least 50%?**<br>• Unclear<br><br>**4. Were all the subjects selected or recruited from the same or similar populations?**<br>• Yes	**5. Was a sample size justification provided?**<br>• No<br><br>**6. Did the study examine exposure levels?**<br>• No<br><br>**7. Were exposure measures clearly defined?**<br>• Not applicable	**8. Were outcome measures clearly defined?**<br>• Yes<br><br>**9. Were outcome assessors blinded?**<br>• Not applicable<br><br>**10. Were confounders adjusted for?**<br>• Unclear
• Reference *Marco-Ruiz et al. 2017 Norway*[24]	**1. Was the research question clearly stated?**<br>• Yes<br><br>**2. Was the study population clearly specified and defined?**<br>• No<br><br>**3. Was the participation rate at least 50%?**	**5. Was a sample size justification provided?**<br>• No<br><br>**6. Did the study examine exposure levels?**<br>• No	**8. Were outcome measures clearly defined?**<br>• Not applicable<br><br>**9. Were outcome assessors blinded?**<br>• Not applicable<br><br>**10. Were confounders adjusted for?**<br>• Unclear

For peer review only

	• Yes<br>*53%*<br><br>**4. Were all the subjects selected or recruited from the same or similar populations?**<br>• Unclear	**7. Were exposure measures clearly defined?**<br>• Not applicable	
• Reference<br>*Nagykaldi 2010* [25]	**1. Was the research question clearly stated?**<br>• Yes<br><br>**2. Was the study population clearly specified and defined?**<br>• Yes<br>*Study population was patients from 12 primary care practices in US.*<br><br>**3. Was the participation rate at least 50%?**<br>• Not applicable<br><br>**4. Were all the subjects selected or recruited from the same or similar populations?**<br>• Yes<br>*All participants were patients from 12 primary care practices that accessed customised practice website or telephone helpline*	**5. Was a sample size justification provided?**<br>• Not applicable<br><br>**6. Did the study examine exposure levels?**<br>• Not applicable<br><br>**7. Were exposure measures clearly defined?**<br>• Not applicable	**8. Were outcome measures clearly defined?**<br>• Yes<br>*Web hits on customised practice website influenza self-management webpages. Downloads of self-management influeza toolkit. Completion of Iflueza self-triage module sessions. Volume of calls to telephone hotlines. Qualitative feedback from patients on statisfaction with and utility of self-management websites and telephone hotline. Qualitative feedback from clinicians around their involvement and their perceptionsof patient self-management techniques.*<br><br>**9. Were outcome assessors blinded?**<br>• Not applicable<br><br>**10. Were confounders adjusted for?**<br>• Not applicable

For peer review only

• Reference *Nijland 2009*[29]	**1. Was the research question clearly stated?**<br>• Yes<br><br>**2. Was the study population clearly specified and defined?**<br>• Yes<br><br>**3. Was the participation rate at least 50%?**<br>• Unclear<br><br>**4. Were all the subjects selected or recruited from the same or similar populations?**<br>• Yes	**5. Was a sample size justification provided?**<br>• No<br><br>**6. Did the study examine exposure levels?**<br>• Not applicable<br><br>**7. Were exposure measures clearly defined?**<br>• Not applicable	**8. Were outcome measures clearly defined?**<br>• Yes<br><br>**9. Were outcome assessors blinded?**<br>• No<br><br>**10. Were confounders adjusted for?**<br>• Yes<br>*Methods not very clearly reported but appears to be multiple regression*
• Reference *Nijland 2016*[19]	**1. Was the research question clearly stated?**<br>• Yes<br><br>**2. Was the study population clearly specified and defined?**<br>• Yes<br><br>**3. Was the participation rate at least 50%?**<br>• No<br>*Low participation rate in survey relative to users of triage system (though unclear how many were invited to participate)*	**5. Was a sample size justification provided?**<br>• No<br><br>**6. Did the study examine exposure levels?**<br>• Not applicable<br><br>**7. Were exposure measures clearly defined?**	**8. Were outcome measures clearly defined?**<br>• Yes<br><br>**9. Were outcome assessors blinded?**<br>• No<br><br>**10. Were confounders adjusted for?**<br>• Unclear

For peer review only

	**4. Were all the subjects selected or recruited from the same or similar populations?**<br>• Yes	• Not applicable	
• Reference<br>*North et. al. 2011*[34]	**1. Was the research question clearly stated?**<br>• Yes<br><br>**2. Was the study population clearly specified and defined?**<br>• Yes<br><br>**3. Was the participation rate at least 50%?**<br>• Not applicable<br><br>**4. Were all the subjects selected or recruited from the same or similar populations?**<br>• Not applicable	**5. Was a sample size justification provided?**<br>• Not applicable<br><br>**6. Did the study examine exposure levels?**<br>• Yes<br>*Self-exposure*<br><br>**7. Were exposure measures clearly defined?**<br>• Not applicable	**8. Were outcome measures clearly defined?**<br>• Yes<br><br>**9. Were outcome assessors blinded?**<br>• Not applicable<br><br>**10. Were confounders adjusted for?**<br>• Unclear<br>*Some discussion of potential confounders.*
• Reference<br>*Sole 2006*[18]	**1. Was the research question clearly stated?**<br>• Yes<br>*"The primary purpose of this study was to identify and describe the demographic profile of students who used the newly implemented Web-based triage system. A secondary purpose was to compare Web-based triage diagnoses to the diagnoses made in clinic for a subset*	**5. Was a sample size justification provided?**<br>• No<br><br>**6. Did the study examine exposure**	**8. Were outcome measures clearly defined?**<br>• Not applicable<br><br>**9. Were outcome assessors blinded?**<br>• Not applicable

For peer review only

	of students who requested appointments"<br><br>**2. Was the study population clearly specified and defined?**<br>• Yes<br>*Students who used the web based triage over a four month implementation period (1290 students). Then of those students, those who requested an appointment via email (143 students), then of those 59 who attended the health centre after requesting an email appointment.*<br><br>**3. Was the participation rate at least 50%?**<br>• Not applicable<br><br>**4. Were all the subjects selected or recruited from the same or similar populations?**<br>• Yes	**levels?**<br>• Yes<br><br>**7. Were exposure measures clearly defined?**<br>• Yes	**10. Were confounders adjusted for?**<br>• Not applicable

For peer review only

Risk of bias results for diagnostic studies

Reference	Questions 1 to 4	Questions 5 to 8	Questions 9 to 11
**Study ID**<br>•<br>Reference<br>*Poote*	**1. Representative spectrum?**<br>• No<br>*Study participants were all patients registered at a student health centre in England attending with new acute*	**5. Differential verification avoided?**<br>• Not applicable?	**9. Relevant clinical information?**<br>• Yes<br><br>**10. Were uninterpretable results reported?**

2014 [17]	*symptoms. If the self-assessment triage system was only for students to be representative the study population would have needed to include range of student health centres in different areas. If the system was for any UK general practices the study population would have needed to include patients of all ages, ethnicity, gender etc from a range GP practices in different areas.*<br><br>**2. Acceptable reference standard?**<br>• Yes<br><br>**3. Acceptable delay between tests?**<br>• Yes<br><br>**4. Partial verification avoided?**<br>• Yes<br>*All patients that completed self-triage also had a GP consultation where the GP rated the urgency of their consultation.*	**6. Was the reference standard independent of the index test?**<br>• Unclear<br>*Patients took the assessment from self-triage through to their GP consultation.*<br><br>**7. Index test results blinded?**<br>• No<br>*Patients took the assessment from self-triage through to their GP consultation.*<br><br>**8. Reference standard results blinded?**<br>• Yes	• Not applicable<br><br>**11. Were withdrawals from the study explained?**<br>• Yes
**Study ID**	**1. Representative spectrum?**	**5. Differential**	**9. Relevant clinical information?**

For peer review only

• Reference *Price 2013* [20]	• No<br>*SORT was only trialled in 2 Emergency Departments in US, a larger range would be needed for a representative spectrum. Also, patients were from ED not home so potentially sicker patients in the sample.*<br><br>**2. Acceptable reference standard?**<br>• Yes<br>*Sensitivity of SORT for kids algorithm in identifying the need for ED care was based on an explicit gold standard: documented evidence that the child received 1 or more of 5 ED-specific interventions.*<br><br>**3. Acceptable delay between tests?**<br>• Yes<br><br>**4. Partial verification avoided?**<br>• Yes	**verification avoided?**<br>• Not applicable?<br><br>**6. Was the reference standard independent of the index test?**<br>• Yes<br><br>**7. Index test results blinded?**<br>• Yes<br><br>**8. Reference standard results blinded?**<br>• Yes	• Yes<br><br>**10. Were uninterpretable results reported?**<br>• Not applicable<br><br>**11. Were withdrawals from the study explained?**<br>• No
**Study ID**<br>• Reference *Semigran 2015* [4]	**1. Representative spectrum?**<br>• Unclear<br>*There were 45 standardised patient vignettes which were divided into three levels of triage urgency and included more and less common conditions. It is not clear how closely this replicates the spectrum of conditions that people use symptom checkers for.*	**5. Differential verification avoided?**<br>• Not applicable?<br><br>**6. Was the reference standard independent of the**	**9. Relevant clinical information?**<br>• Yes<br>*This is the clinical information that would be supplied by the patient which may or may not differ from the information given by the vignette.*<br>[#548 Semigran 2015.pdf] Page 8: *ion of the true clinical accuracy of symptom checkers.33 Some standardized patient vignettes con- tained specifc clinical language (for*

	**2. Acceptable reference standard?**<br>• Yes<br>[#548 Semigran 2015.pdf] Page 2: *The source for each vignette also provided the associated correct diagnosis.*<br><br>**3. Acceptable delay between tests?**<br>• Not applicable<br><br>**4. Partial verification avoided?**<br>• Not applicable	**index test?**<br>• Yes<br><br>**7. Index test results blinded?**<br>• Yes<br><br>**8. Reference standard results blinded?**<br>• Yes	*example, mouth ulcers, tonsils with exudate), and actual patients with the same condition might struggle with the words to use to describe their symptoms or use diferent terms. Therefore, our analysis represents an indirect assess- ment of how well symptom checkers would perform with actual patients*<br><br>**10. Were uninterpretable results reported?**<br>• Yes<br>[#548 Semigran 2015.pdf] Page 3: *ns for diagnosis and triage was high (Cohen's κ 0.90). In some cases we could not evaluate a vignette because some symptom checkers focus only on children or on adults or the symptom checker did not list or ask for the key symp- tom in the vignette. To avoid penalizing these symptom checkers, we referred to standardized patient vignettes that successfully yielded an output as "standardized patient evaluations."*<br><br>**11. Were withdrawals from the study explained?**<br>• Not applicable
**Study ID**<br>•<br>Reference *Semigran 2016* [8]	**1. Representative spectrum?**<br>• Unclear<br>*There were 45 standardised patient vignettes which were divided into three levels of triage urgency and included more and less common conditions. It is not clear how closely this replicates the spectrum of conditions that people use symptom checkers for.*	**5. Differential verification avoided?**<br>• Not applicable?<br><br>**6. Was the**	**9. Relevant clinical information?**<br>• Yes<br>*The physicians and the symptom checkers used the same vignettes*<br><br>**10. Were uninterpretable results reported?**

	**2. Acceptable reference standard?**<br>• Yes<br><br>**3. Acceptable delay between tests?**<br>• Not applicable<br><br>**4. Partial verification avoided?**<br>• No<br>*There was a total of 234 physicians involved in the study and of the 45 vignettes, each was solved by at least 20 physicians but it is not clear why they chose the specific vignettes to solve.*	**reference standard independent of the index test?**<br>• Not applicable<br><br>**7. Index test results blinded?**<br>• Yes<br><br>**8. Reference standard results blinded?**<br>• Yes	• Not applicable<br><br>**11. Were withdrawals from the study explained?**<br>• No<br>*It is unclear why the physicians chose to solve the specific vignettes*

For peer review only

PRISMA 2009 Checklist

Section/topic	#	Checklist item	Reported on page #
**TITLE**			
Title	1	Identify the report as a systematic review, meta-analysis, or both.	1
**ABSTRACT**			
Structured summary	2	Provide a structured summary including, as applicable: background; objectives; data sources; study eligibility criteria, participants, and interventions; study appraisal and synthesis methods; results; limitations; conclusions and implications of key findings; systematic review registration number.	2-3
**INTRODUCTION**			
Rationale	3	Describe the rationale for the review in the context of what is already known.	4-5
Objectives	4	Provide an explicit statement of questions being addressed with reference to participants, interventions, comparisons, outcomes, and study design (PICOS).	5
**METHODS**			
Protocol and registration	5	Indicate if a review protocol exists, if and where it can be accessed (e.g., Web address), and, if available, provide registration information including registration number.	5
Eligibility criteria	6	Specify study characteristics (e.g., PICOS, length of follow-up) and report characteristics (e.g., years considered, language, publication status) used as criteria for eligibility, giving rationale.	6
Information sources	7	Describe all information sources (e.g., databases with dates of coverage, contact with study authors to identify additional studies) in the search and date last searched.	5-6
Search	8	Present full electronic search strategy for at least one database, including any limits used, such that it could be repeated.	Appendix 1
Study selection	9	State the process for selecting studies (i.e., screening, eligibility, included in systematic review, and, if applicable, included in the meta-analysis).	6
Data collection process	10	Describe method of data extraction from reports (e.g., piloted forms, independently, in duplicate) and any processes for obtaining and confirming data from investigators.	7
Data items	11	List and define all variables for which data were sought (e.g., PICOS, funding sources) and any assumptions and simplifications made.	7
Risk of bias in individual studies	12	Describe methods used for assessing risk of bias of individual studies (including specification of whether this was done at the study or outcome level), and how this information is to be used in any data synthesis.	7
Summary measures	13	State the principal summary measures (e.g., risk ratio, difference in means).	N/A
Synthesis of results	14	Describe the methods of handling data and combining results of studies, if done, including measures of consistency (e.g., I^2) for each meta-analysis.	N/A

For peer review only

For peer review only - http://bmjopen.bmj.com/site/about/guidelines.xhtml

PRISMA 2009 Checklist

Section/topic	#	Checklist item	Reported on page #
Risk of bias across studies	15	Specify any assessment of risk of bias that may affect the cumulative evidence (e.g., publication bias, selective reporting within studies).	N/A
Additional analyses	16	Describe methods of additional analyses (e.g., sensitivity or subgroup analyses, meta-regression), if done, indicating which were pre-specified.	7
**RESULTS**			
Study selection	17	Give numbers of studies screened, assessed for eligibility, and included in the review, with reasons for exclusions at each stage, ideally with a flow diagram.	9
Study characteristics	18	For each study, present characteristics for which data were extracted (e.g., study size, PICOS, follow-up period) and provide the citations.	10-16
Risk of bias within studies	19	Present data on risk of bias of each study and, if available, any outcome level assessment (see item 12).	Appendix 2
Results of individual studies	20	For all outcomes considered (benefits or harms), present, for each study: (a) simple summary data for each intervention group (b) effect estimates and confidence intervals, ideally with a forest plot.	17-22
Synthesis of results	21	Present results of each meta-analysis done, including confidence intervals and measures of consistency.	N/A
Risk of bias across studies	22	Present results of any assessment of risk of bias across studies (see Item 15).	N/A
Additional analysis	23	Give results of additional analyses, if done (e.g., sensitivity or subgroup analyses, meta-regression [see Item 16]).	22-23
**DISCUSSION**			
Summary of evidence	24	Summarize the main findings including the strength of evidence for each main outcome; consider their relevance to key groups (e.g., healthcare providers, users, and policy makers).	26-27
Limitations	25	Discuss limitations at study and outcome level (e.g., risk of bias), and at review-level (e.g., incomplete retrieval of identified research, reporting bias).	28
Conclusions	26	Provide a general interpretation of the results in the context of other evidence, and implications for future research.	28-29
**FUNDING**			
Funding	27	Describe sources of funding for the systematic review and other support (e.g., supply of data); role of funders for the systematic review.	29

From: Moher D, Liberati A, Tetzlaff J, Altman DG, The PRISMA Group (2009). Preferred Reporting Items for Systematic Reviews and Meta-Analyses: The PRISMA Statement. PLoS Med 6(7): e1000097. doi:10.1371/journal.pmed1000097

For more information, visit: **www.prisma-statement.org**.

For peer review only - http://bmjopen.bmj.com/site/about/guidelines.xhtml

BMJ Open

Digital and online symptom checkers and health assessment/triage services for urgent health problems: systematic review

Journal:	*BMJ Open*
Manuscript ID	bmjopen-2018-027743.R1
Article Type:	Research
Date Submitted by the Author:	02-Apr-2019
Complete List of Authors:	Chambers, Duncan ; The University of Sheffield, ScHARR<br>Cantrell, Anna; The University of Sheffield, ScHARR<br>Johnson, Maxine; The University of Sheffield, ScHARR<br>Preston, Louise; The University of Sheffield, ScHARR<br>Baxter, Susan; The University of Sheffield, ScHARR<br>Booth, Andrew; The University of Sheffield, ScHARR<br>Turner, Janette; The University of Sheffield, ScHARR
**Primary Subject Heading**:	Health services research
Secondary Subject Heading:	Diagnostics
Keywords:	urgent care, symptom checkers, systematic reviews

SCHOLARONE™
Manuscripts

Digital and online symptom checkers and health assessment/triage services for urgent health problems: systematic review

Duncan Chambers[1], Anna Cantrell[1], Maxine Johnson[1], Louise Preston[1], Susan K Baxter[1], Andrew Booth[1] and Janette Turner[1]

[1]School of Health and Related Research (ScHARR), University of Sheffield, Regent Court, Sheffield S1 4DA, UK

*Correspondence to Duncan Chambers: d.chambers@sheffield.ac.uk

Contributor/guarantor information:

DC contributed to the planning (project co-ordination and protocol development), conduct (study selection, data extraction and quality assessment) and reporting (report writing) of the study. AC contributed to the planning (protocol development), conduct (information retrieval, study selection, data extraction and quality assessment) and reporting (report writing) of the study. MJ contributed to the planning (protocol development), conduct (study selection, data extraction and quality assessment) and reporting (report writing) of the study. LP contributed to the planning (protocol development), conduct (study selection, data extraction and quality assessment) and reporting (report writing) of the study. SB contributed to the planning (protocol development), conduct (study selection, data extraction and quality assessment) and reporting (report writing) of the study. AB contributed to the planning (protocol development), conduct (information retrieval and study selection) and reporting (report writing) of the study. JT contributed to the planning, conduct and reporting of the study by providing expert topic advice at all stages. All the authors contributed to the study conception and design (protocol development), acquisition of data (study selection and data extraction) and analysis or interpretation of data (writing sections and/or commenting on drafts of the report). Duncan Chambers is the guarantor for this work. The corresponding author attests

For peer review only

that all listed authors meet authorship criteria and that no others meeting the criteria have been omitted.

Competing interests

None of the authors have any competing interests

Copyright

The Corresponding Author has the right to grant on behalf of all authors and does grant on behalf of all authors, a non-exclusive worldwide licence to the Publishers and its licensees in perpetuity, in all forms, formats and media (whether known now or created in the future), to i) publish, reproduce, distribute, display and store the Contribution, ii) translate the Contribution into other languages, create adaptations, reprints, include within collections and create summaries, extracts and/or, abstracts of the Contribution, iii) create any other derivative work(s) based on the Contribution, iv) to exploit all subsidiary rights in the Contribution, v) the inclusion of electronic links from the Contribution to third party material where-ever it may be located; and, vi) licence any third party to do any or all of the above.

For peer review only

Abstract

Objectives: In England, the NHS111 service provides assessment and triage by telephone for urgent health problems. A digital version of this service has recently been introduced. We aimed to systematically review the evidence on digital and online symptom checkers and similar services.

Design: Systematic review.

Data sources: We searched MEDLINE, EMBASE, the Cochrane Library, CINAHL, HMIC (Health Management Information Consortium), Web of Science and ACM Digital Library up to April 2018, supplemented by phrase searches for known symptom checkers and citation searching of key studies.

Eligibility criteria: Studies of any design that evaluated a digital or online symptom checker or health assessment service for people seeking advice about an urgent health problem.

Data extraction and synthesis: Data extraction and quality assessment (using the Cochrane Collaboration version of QUADAS for diagnostic accuracy studies and the National Heart, Lung and Blood Institute tool for observational studies) -were done by one reviewer with a sample checked for accuracy and consistency. We performed a narrative synthesis of the included studies structured around pre-defined research questions and key outcomes.

Results: We included 29 publications (27 studies). Evidence on patient safety was weak. Diagnostic accuracy varied between different systems but was generally low. Algorithm-based triage tended to be more risk-averse than that of health professionals. There was very limited evidence on patients' compliance with online triage advice. Study participants generally expressed high levels of satisfaction, albeit in mainly uncontrolled studies. Younger and more highly educated people were more likely to use these services.

Conclusions: The English 'digital 111' service has been implemented against a background of uncertainty around the likely impact on important outcomes. The health system may need to respond to short-term changes and/or shifts in demand. The popularity of online and digital services with younger and more educated people has implications for health equity.

Registration: PROSPERO (registration number CRD42018093564)

Strengths and limitations of this study

For peer review only - http://bmjopen.bmj.com/site/about/guidelines.xhtml

- This systematic review was based on a rigorous search of the literature which maximised efficiency by combining an initial focused search with subsequent rounds of follow-up searching, including searches for named symptom checker systems.
- Our narrative synthesis approach used a mixture of description and tabulation to summarise the evidence, including overall strength of the evidence base for each of the pre-specified outcomes of interest.
- Given the decision to implement a national urgent care service based on digital symptom checkers in the NHS in England, our study highlights areas of uncertainty that will need to be resolved by research and data collection.
- The review inclusion criteria were relatively broad and findings from symptom checker systems for specific conditions may not be applicable to more general systems and vice versa.
- We have also included studies of symptom checkers as part of electronic consultation systems in general practice, which again represents a slightly different setting from a general 'digital 111' service, and this should be kept in mind when interpreting the results.

For peer review only

Introduction

Digital and online symptom checkers and assessment services are used by patients seeking guidance about health problems, including some that may require urgent action. These services generally provide people with possible alternative diagnoses based on their reported symptoms and/or suggest a course of action (e.g. self-care, make a GP appointment or go to an emergency department (ED)).

In England, the NHS111 service provides assessment and triage by telephone for problems that are urgent but not classified as emergencies. The latest data from NHS England[1] show that in September2018 there were over 1.27 million calls to NHS111, an average of 42,400 per day. Outcomes of these calls were that 13.2% had ambulances despatched; 9.5% were recommended to attend an ED; 58.7% were recommended to attend primary care; 4.8% to attend another service; and 13.8% were not recommended to attend another service (e.g. their condition was considered suitable for self-care)

NHS England has recently introduced a digital platform to make NHS111accessible via a website or smartphone app. A beta version of the service (referred to as 'NHS111 Online') is available at https://111.nhs.uk/ (accessed 1 April 2019). The 'digital 111' service is seen as key to reducing demand for the telephone 111 service, enabling resources to be redirected to supporting 'integrated urgent and emergency care systems' as outlined in the 'NHS 5-year Forward View' and its 2017 update 'Next Steps on the NHS 5-year Forward View'[2 3].

There is an expectation that a digital 111 platform will help to manage demand and increase efficiency in the urgent and emergency care system, complementing the agenda of locally based Sustainability and Transformation Partnerships (STPs) which involve the health service and local government working together to integrate and co-ordinate care[4]. However, there is a risk of increasing demand, duplicating healthcare contacts (by increasing the number of potential access routes into the system) and providing advice that is not safe or clinically appropriate. For example, an evaluation of the NHS111 telephone service at four pilot sites and three control sites found that in its first year the service was not successful in reducing 999 emergency calls or in shifting patients from emergency to urgent care[5]. A recent study of 23 symptom checker algorithms providing diagnostic and triage advice that would form the

For peer review only - http://bmjopen.bmj.com/site/about/guidelines.xhtml

basis of a ‘digital 111’ platform found deficiencies in both their diagnostic and triage capabilities (based on patient vignettes)[6].

In 2017, NHS England carried out pilot evaluations of different systems in four regions of England. The evaluations aimed to assess whether digital/online triage was acceptable to users and connected them to appropriate clinical care[7]. The full report of the evaluations was not yet published at the time of writing. The objective of this systematic review was to inform further development of the proposed digital platform by summarising and critiquing the previous research in this area, both from the UK and overseas. The overall research question was: for people seeking guidance about an urgent health problem, what is the effect of digital and online services designed to assess symptoms and signpost patients to appropriate services (compared with non-digital services or no comparator) on important clinical and health service outcomes? Outcomes include safety; clinical and cost-effectiveness; diagnostic and triage accuracy; impact on service use; patient/carer satisfaction; compliance with advice received; and outcomes related to equity and inclusion.

Methods

The review protocol was registered with PROSPERO (registration number CRD42018093564) and is available from the project website (https://www.journalslibrary.nihr.ac.uk/programmes/hsdr/164717/).

Literature search and screening

Initial scoping searches revealed that a highly sensitive search strategy, as typically conducted for systematic reviews, retrieved a disproportionately high number of references on GP decision-making and triage as demonstrated by examination of sample search results (e.g. first 100). We therefore devised a three stage retrieval strategy as an acceptable alternative to comprehensive topic-based searching. This involved:

1. Targeted searches of precise high specificity terms in seven databases (MEDLINE, EMBASE, the Cochrane Library, CINAHL, HMIC (Health Management Information Consortium), Web of Science and ACM Digital Library). These searches were not restricted by language or date. Search strategies are presented in Appendix 1.

For peer review only - http://bmjopen.bmj.com/site/about/guidelines.xhtml

2. Phrase searching for names of known symptom checkers using a list compiled from Semigran 2015 and other sources

3. Citation searches and reference checking of key included studies and reviews, complemented by contact with service providers (directly and via websites).

The main literature search was completed in April 2018 and follow-up searches in May 2018. Search results were stored in a reference management system (EndNote) and imported into EPPI-Reviewer software for screening, data extraction and quality assessment. The search results were screened against the inclusion criteria by one reviewer, with a 10% random sample screened by a second reviewer. Uncertainties were resolved by discussion among the review team.

Inclusion and exclusion criteria

Population: General population seeking information online or digitally to address an urgent health problem, including adults and children and issues arising from both acute and long-term chronic illness.

Intervention: Any online or digital service designed to assess symptoms, provide health advice and direct patients to appropriate services. Services that only provide health advice were excluded, as were those that offer treatment, e.g. online CBT services.

Comparator: The ‘gold standard’ comparator is current practice of telephone assessment (e.g. NHS111) or face to face assessment (e.g. general practice, urgent care centre or ED). However, studies with other relevant comparators (e.g. comparative performance in tests or simulations) or with no comparator were included if they addressed the research questions.

Outcomes: The main outcomes of interest were safety (e.g. any evidence of adverse events arising from following or ignoring advice from online/digital services); clinical effectiveness; costs/cost-effectiveness; accuracy; impact on service use; compliance with advice received; patient/carer satisfaction; and equity and inclusion. ‘Accuracy covered 1) ability to provide a correct diagnosis and 2) ability to distinguish between high and low acuity/urgency problems (and hence direct patients to appropriate services).

Study design: We did not restrict inclusion by study design (and included relevant audits or service evaluations in addition to formal research studies) but included studies had to evaluate (quantitatively or qualitatively) some aspect of an online/digital service

Other: Studies from any developed country healthcare system were eligible for inclusion

For peer review only - http://bmjopen.bmj.com/site/about/guidelines.xhtml

Excluded: Purely descriptive studies, conceptual papers, projections of possible future developments and studies conducted in low or middle income countries were excluded from the review.

Data extraction and quality/strength of evidence assessment

We extracted and tabulated key data from the included studies, including study design, population/setting, results and key limitations. Data extraction was performed by one reviewer, with a 10% random sample checked for accuracy and consistency.

To characterise the included digital and online systems as interventions, we identified studies reporting on a particular system and extracted data from all relevant studies using a modification of the TIDieR (Template for Intervention Description and Replication) checklist[8] which we designated TIDieST (Template for Intervention Description for Systems for Triage). Further details may be found in the full report (Chambers et al., *Health Services & Delivery Research* 2019 (in press)).

Quality (risk of bias) assessment was undertaken for peer-reviewed full publications only (i.e. not grey literature publications (such as research reports, working papers, or reports produced by government departments, academics, business and industry) or conference abstracts). Randomised controlled trials were assessed using the Cochrane Collaboration risk of bias tool. For diagnostic accuracy type studies, we used the Cochrane Collaboration version of QUADAS[9] and for other study designs we used the National Heart Lung and Blood Institute tool for observational cohort and cross-sectional studies (https://www.nhlbi.nih.gov/health-topics/study-quality-assessment-tools, accessed 25th March 2019). Quality assessment was performed by one reviewer, with a random 10% sample checked for accuracy and consistency.

Assessment of the overall strength (quality and relevance) of evidence for each research question is part of the narrative synthesis. Overall strength of the evidence base for key outcomes was assessed using an adaptation of the method described by Baxter et al.[10] This involves classifying evidence as ‘stronger’, ‘weaker’, ‘conflicting’ or ‘insufficient’ based on study numbers and design. Specifically, “stronger evidence” represented generally consistent findings in multiple studies with a comparator group design or comparative diagnostic

For peer review only - http://bmjopen.bmj.com/site/about/guidelines.xhtml

accuracy studies; "weaker evidence" represented generally consistent findings in one study with a comparator group design and several non-comparator studies or multiple non-comparator studies; "very limited evidence" represented an outcome reported by a single study; and finally, "inconsistent evidence" represented an outcome where fewer than 75% of studies agreed on the direction of effect. All studies in the review, including those that did not meet criteria for risk of bias assessment, were included in the strength of evidence assessment.

Evidence synthesis

We performed a narrative synthesis structured around the pre-specified research questions and outcomes. We did not perform any meta-analyses because the included studies varied widely in terms of design, methodology and outcomes.

Patient and public involvement (PPI)

The review was discussed at two meetings of an existing PPI group covering the programme from which the review was commissioned (Sheffield HS&DR Evidence Synthesis Centre). At the meetings there was discussion regarding the focus of the work, including a presentation on previous research on NHS111 telephone services to provide a context for understanding the current work. The meetings also included presentation and discussion of the findings of the review, in order to explore key messages for patients which could inform dissemination of the findings. Discussion during one meeting was structured using a SWOT (strengths, weaknesses, opportunities and threats) analysis approach, which revealed a number of potential concerns amongst patients (e.g. reliability and consistency; high costs of programming and development; whether patients would follow advice given; and threats to equity) as well as potential perceived benefits (e.g. improved access to care at all hours; value to those who might feel embarrassed discussing their problem with a health professional). Involvement of the advisory group was beneficial in highlighting some issues that had also emerged from the systematic review, and enabled the reviewers to structure the review findings taking this into account. For example, the group's uncertainty about the likely impact of 'digital 111' was reflected in the review findings and recommendations for ongoing evaluation and further research. The review report also reflects the group's relatively cautious attitude (while recognising the need to update the way services are accessed) which contrasts with the strong belief in some quarters that 'digital 111' will help to ensure that patients

For peer review only - http://bmjopen.bmj.com/site/about/guidelines.xhtml

receive appropriate care more quickly while reducing ‘inappropriate’ visits to EDs and GP appointments.

Results

Results of literature search

Twenty-seven studies (29 publications) were included in the review. Figure 1 presents the flow of studies through the selection process. Inter-rater agreement on initial study selection was moderate (Kappa = 0.582). This reflects a degree of learning by the review team: our initial sift of the search results consciously favoured inclusivity and items found not to meet the inclusion criteria on detailed examination were subsequently discarded.

For peer review only

For peer review only - http://bmjopen.bmj.com/site/about/guidelines.xhtml

Figure 1: PRISMA flow diagram

Characteristics of included studies

Seventeen studies (Table 1) evaluated symptom checkers as a self-contained intervention, of which eight covered a limited range of symptoms, e.g. respiratory[11-13] or gastrointestinal[14 15] symptoms which we considered to be 'urgent'. The remaining studies in this group evaluated symptom checkers covering a wider range of common urgent care symptoms. Studies either evaluated a single system[16-19] or multiple systems[6 20]. We found only one study of a symptom checker specifically intended for assessment of children's symptoms, a development of the SORT (Strategy for Off-Site Rapid Triage) system for influenza-like illness[21] Two reports with some overlap of content evaluated the 'babylon check' app[16 22]

Five studies[7 23-26] evaluated symptom checkers as part of a broader self-assessment and consultation system (often referred to as electronic consultation or e-consultation). Study characteristics are summarised in Table 2. In this type of system, the role of symptom checkers is to help patients decide whether their symptoms require a consultation with a doctor or other health professional or can be dealt with by self-care. If a consultation is required, details of the symptoms and a request for an appointment or call-back can be submitted electronically. This type of study is important because it considers the service within the broader context of the urgent and emergency care system. A limitation is that some studies focused mainly on the 'downstream' elements of the pathway, e.g. consultation with GPs, and provided limited data on the symptom checker element of the system.

A final group of five studies examined patient and/or public attitudes to online self-diagnosis in the context of urgent care[27-31].See the full report for further details (Chambers et al. *Health Services & Delivery Research* 2019 (in press)).

For peer review only - http://bmjopen.bmj.com/site/about/guidelines.xhtml

Table 1: Studies of symptom checkers as a self-contained intervention

Reference	Study design	System type	Comparator	Population/sample
Babylon Health 2017[22]	• Uncontrolled observational<br>*No control group but some comparison with NHS111 telephone data*	• Digital<br>*Smartphone app*	• Health professional performance on real-world data<br>• Other<br>*NHS111 data for 12 months from February 2017*	• General population<br>*Participants in the London pilot evaluation of 'digital 111' services*
Berry 2016[14]	• Simulation<br>*Evaluation of symptom checker performance on clinical vignettes*	• Online<br>*17 symptom checkers*	• None	• Specific condition(s)<br>*Gastrointestinal symptoms*
Berry 2017[32]	• Controlled observational	• Online<br>*Three online symptom checkers (WebMD, iTriage and FreeMD)*	• Health professional performance on real-world data	• Specific condition(s)<br>*Patients with a cough presenting to an internal medicine clinic*
Berry 2017[15]	• Controlled observational	• Online<br>*Three online symptom checkers (WebMD, iTriage, FreeMD)*	• Health professional performance on real-world data	• Specific condition(s)<br>*Abdominal pain*
Kellermann 2010 [11]	• Simulation<br>*The developed algorithm was tested against past patient records..*	• Online<br>*SORT was available on 2 interactive websites*	• Health professional performance on real-world data<br>*The algorithm was tested against clinicians' decision on past patient records.*	• Specific condition(s)<br>*Influenza symptoms*

For peer review only - http://bmjopen.bmj.com/site/about/guidelines.xhtml

Little 2016[12]	• Experimental<br>*Randomised controlled trial (RCT)*	• Online<br>*'Internet Doctor' website*	• Other<br>*Usual GP care without access to the Internet Doctor website*	• Specific condition(s)<br>*Respiratory infections and associated symptoms*
Luger et al. 2014 [33]	• Simulation<br>*Described as "human-computer interaction study" using think-aloud protocols.*	• Online<br>*Google and WebMD*	• Other<br>*Comparing two internet health tools.*	• General population<br>*Older adults (50 years or older)*
Marco-Ruiz et al. 2017 [34]	• Qualitative<br>*Qualitative element*<br>• Other<br>*1. Online evaluation by users (problem detection) 2. Think aloud technique by smaller sample of participants (usability)*	• Online<br>*Erdusyk*	• None	• General population<br>*Internet tool users*
Middleton 2016[16]	• Simulation	• Digital<br>*'babylon check' automatic triage system*	• Health professional performance on test/simulation<br>*Twelve 'clinicians' (doctors) and 17 nurses*	• General population
Nagykaldi 2010 [35]	• Uncontrolled observational	• Online<br>*Customised practice website including a bilingual influenza self-triage module, a downloadable influenza toolkit and electronic messaging capability. A bilingual seasonal influenza telephone hotline was*	• None	• Specific condition(s)<br>*Influenza*

For peer review only - http://bmjopen.bmj.com/site/about/guidelines.xhtml

		available as an alternative.		
Nijland 2016[19]	• Uncontrolled observational *Retrospective analysis of 15 months' data*	• Online *Web-based triage system (http://www.dokterdokter.nl)*	• None	• General population
Poote 2014 [17]	• Uncontrolled observational	• Online *Prototype self-assessment triage system*	• Health professional performance on real-world data *GPs triage rating was compared with rating from the self-assessment system*	• General population *Students attending a University Student Health Centre with new acute symptoms*
Price 2013 [21]	• Uncontrolled observational	• Online *A web-based decision support tool - Strategy for Off-site Rapid Triage (SORT) for Kids designed to help parents and adult caregivers decide whether a child with possible influenza symptoms needs to visit the emergency department for immediate care.*	• Health professional performance on real-world data *The sensitivity of the algorithm was compared with a gold standard - evidence form child's medical records that they received 1 or more of 5 ED-specific interventions.*	• Specific condition(s) *Influenza in children*
Semigran 2015 [6]	• Experimental *Described as an audit study*	• Multiple *23 symptom checkers were evaluated. Symptom checkers available as apps (via the App Store and Google Play) were identified through searching for "symptom checker" and "medical diagnosis" and screened the first 240 results. Symptom checkers available online were identified through searching Google and Google Scholar for "symptom checker"*	• Other *Vignettes had a diagnosis and triage attached to them and these were compared against the symptom checker advice.*	• General population *Where a single class of illness was examined by the symptom checker, the symptom checker was excluded from the study.*

For peer review only - http://bmjopen.bmj.com/site/about/guidelines.xhtml

		and "medical diagnosis" and screened the first 300 results.		
Semigran 2016 [20]	• Experimental<br>*Comparison of physician and symptom checker diagnoses based on clinical vignettes*	• Multiple<br>*"Human Dx is a web-and app based platform"*	• Health professional performance on test/simulation<br>*Clincial vignettes - a comparison of 23 symptom checkers with physician diagnosis for 45 vignettes*	• General population<br>*Of the 45 condition vignettes - there were 15 low, 15 medium and 15 huigh acuity vignettes - there were 26 common and 19 uncommon condition vignettes*
Sole 2006 [18]	• Uncontrolled observational<br>*Descriptive comparative study*	• Online<br>*A web-based triage system (24/7 WebMed)*	• Health professional performance on real-world data<br>*Data was evaluated from students who had used the web based triage and then requested an appointment via email (so triage data was available for comparison).*	• General population
Yardley 2010[13]	• Experimental<br>*Exploratory randomised trial*	• Online<br>*'Internet Doctor' website*	• Other<br>*Self-care information provided as a static web page with no symptom checker or triage advice*	• Specific condition(s)<br>*Minor respiratory symptoms, e.g. cough, sore throat, fever, runny nose*

For peer review only

For peer review only - http://bmjopen.bmj.com/site/about/guidelines.xhtml

Table 2: Studies of symptom checkers as part of an electronic consultation system

Reference	Study design	System type	Comparator	Population/sample
Carter 2018 [23]	• Uncontrolled observational<br>*Mixed-methods evaluation*	• Online<br>*webGP(subsequently known as eConsult)*	• Other<br>*Investigate patient experience by surveying patients who had used webGP and comparing their experience with controls (patients who had received a face-to-face consultation during the same time period) matched for age and gender*	• General population<br>*General practices in NHS Northern, Eastern and Western Devon Clinical Commissioning Group's area*
Cowie 2018[24]	• Uncontrolled observational<br>*6-month evaluation at 11 GP practices in Scotland*	• Online<br>*eConsult, accessed via GP surgery websites. Service provides self-care assessment and advice, including symptom checkers; triage and signposting to alternative services; access to NHS24 (phone service); and e-consults allowing submission of details by e-mail)*	• None	• General population<br>*Patients registered with participating GP practices*
Madan 2014[25]	• Uncontrolled observational<br>*Report of 6-month pilot study*	• Online<br>*webGP (subsequently known as eConsult)*	• None	• General population
NHS England[7]	• Uncontrolled observational<br>*Analysis of data from four pilot studies together with data from other*	• Multiple<br>*Pilots featured NHS Pathways (Web-based; West Yorkshire); Sense.ly ('voice-activated avatar'; West Midlands); Espert 24 (Web-based; Suffolk) and babylon (app; North Central London)*	• None<br>*Authors stated it was not appropriate to compare pilot sites because of differences in starting date, 'footprints' covered, method of uptake and underlying population*	• General population

For peer review only - http://bmjopen.bmj.com/site/about/guidelines.xhtml

	sources			
Nijland 2009[26]	• Other<br>*Online survey*	• Online<br>*Responses of interest relate to 'indirect e-consultation' (consulting a GP via secure e-mail with intervention of a Web-based triage system)*	• None	• General population<br>*Patients with Internet access but no experience of e-consultation*

For peer review only

For peer review only - http://bmjopen.bmj.com/site/about/guidelines.xhtml

Results by outcome

Safety

None of the six included studies that reported on safety outcomes identified any problems or differences in outcomes between symptom checkers and health professionals. Most of the studies compared system performance with that of health professionals using real or simulated data. The only study with no comparison group was the 6-month pilot study of webGP[25], which reported 'no major incidents'.

Limitations of the studies included not being based on real patient data[16]; covering only a limited range of conditions[11 21]; and sampling a young healthy population (students) not representative of the general population of users of the urgent care system[17]. Studies of e-consultation systems did not generally collect data on those respondents who decided not to seek an appointment, limiting their ability to assess any impact on safety for this group. Overall, the evidence should be interpreted cautiously as indicating no evidence of a detrimental impact on safety rather than evidence of no detrimental effect.

Clinical effectiveness

Only two studies reported on clinical effectiveness outcomes, making it difficult to draw any firm conclusions. In the study by Little et al., those who used the Internet Doctor website experienced longer illness duration and more days of illness rated moderately bad or worse than the usual care group[12] The pilot study of the webGP system[25] reported that several patients received advice to seek treatment for serious symptoms that might otherwise have been ignored. However, no details or quantitative data were provided.

Costs/cost-effectiveness

Two included studies provided limited data on possible cost savings. Based on 6 months of pilot data, Madan[25]estimated savings of £11,000 annually for an average general practice (6,500 patients) compared with current practice. The report also suggested a saving to commissioners equivalent to £414,000 annually for a CCG (Clinical Commissioning Group, responsible for specifying and purchasing most health services in the NHS in England) covering 250,000 patients. These savings were specifically related to self-reported diversion of patients from GP appointments to self-care and from urgent care to e-consultation. Using

For peer review only - http://bmjopen.bmj.com/site/about/guidelines.xhtml

similar methodology, the manufacturers of the ‘babylon check’ app claimed average savings of over £10/triage compared with NHS111 by telephone, based on a higher proportion of patients being recommended to self-care[22].

Diagnostic accuracy

Eight studies reported at least some data on the diagnostic accuracy of symptom checkers. In spite of the diverse methods and comparisons in the included studies, almost all agreed that the diagnostic accuracy of symptom checkers was poor in absolute terms (e.g. in evaluating ‘vignettes’ designed to test knowledge of specific conditions, where the correct diagnosis was already known by definition) or relative to that of health professionals. In the most comprehensive evaluation, Semigran et al. evaluated 23 symptom checkers across 770 standardised patient evaluations[6]. Overall the correct diagnosis was made in 34% of cases (95% CI 31%-37%), although performance varied widely between symptom checkers, high and low acuity conditions and common and rare conditions. When the same authors compared the 23 symptom checkers with physicians using 43 vignettes, physicians were more likely to list the correct diagnosis first (out of three differential diagnoses) (72.1% vs. 34% $p<0.001$) as well as among the top three diagnoses (84.3% vs. 51.2% $p<0.001$)[20].

The only exception to the rule was an evaluation carried out at a student health centre[18]. Using data from 59 participants who used the 24/7 WebMed system and who were subsequently treated at the health centre, the study found good agreement between chief complaint, 24/7 WebMed classification and provider diagnosis (kappa values 0f 0.89 to 0.94). This study differed from the others in using data from students rather than a general population sample. In addition, the students’ complaints were generally common and uncomplicated, a scenario in which symptom checkers performed relatively well in the study by Semigran et al.[20].

Accuracy of disposition (triage and signposting to appropriate services)

Six included studies reported on this outcome, all except one of which[15] evaluated a ‘general purpose’ symptom checker. As with diagnostic accuracy, diverse methodologies and outcome measures were used.

For peer review only - http://bmjopen.bmj.com/site/about/guidelines.xhtml

The results overall presented a mixed picture but most studies indicated that symptom checkers were inferior and/or more cautious in their triage advice compared with doctors or other health professionals. In their review of 23 symptom checkers, Semigran et al. found that the systems provided appropriate triage advice in 57% (95% CI 52% to 61%) of cases[6]. Performance varied across the systems evaluated, correct triage ranging from 33% to 78%. The NHS England pilot evaluation of four systems[7] found that agreement with clinical experts varied from 30% to 95%, although the number of responses also varied, reducing the comparability of the results.

For abdominal pain, Berry et al. evaluated three symptom checkers and found that 33% of diagnoses were at the same level of urgency as physician diagnoses (emergency, non-emergency or self-care); 39% were diagnosed as more serious and 30% less serious than the physician's judgement[15]. A similar level of agreement between algorithm and clinician (39%) was reported by Poote et al.[17], while the system evaluated by Nijland et al. advised patients to visit a doctor in 85% of cases, even when the symptoms were appropriate for self-care[19].

The only studies to report clearly equal or superior accuracy of disposition using an automated system were the evaluations of Babylon check by the company that developed the system. Middleton et al.[16] reported that using patient vignettes, the app gave an accurate triage outcome in 88.2% of cases, compared with 75.5% for doctors and 73.5% for nurses (unaware of the 'correct' diagnosis for the vignettes). When vignettes were delivered by a medical professional rather than actors, the accuracy of Babylon check increased to over 90%. A later report looked at triage results obtained as part of the NHS England pilot evaluation, concluding that all of 74 referrals to urgent or emergency care were appropriate[22].

Impact on service use/diversion

Eight studies reported on this outcome, although one of them[11] merely stated that it was not possible to assess the effect of the intervention (a web-based influenza triage system) on patients' use of health services.

The pilot evaluation of the webGP system reported that 18% of users planned to book an appointment but chose not to do so[25] In addition, 14% of users reported that they would have

For peer review only - http://bmjopen.bmj.com/site/about/guidelines.xhtml

attended a walk-in centre or other urgent care service if they had not had access to the webGP system.

The NHS England pilot evaluation of four online/digital systems in different regions of England[7] compared the recommendations of the digital systems with those of the NHS111 telephone service over a similar time period (the first months of 2017). Compared with the telephone service, the online and digital services directed a slightly higher proportion of patients to self-care (18% vs. 14%) and a lower proportion to other primary care services such as GPs, dental and pharmacy (40 vs. 60%). The manufacturer's data on the 'babylon check' app collected as part of the NHS England evaluation indicated that patients were more likely to be triaged to self-care by the app compared with NHS111 by telephone (40 vs. 14%)[22]. This figure includes people who received information leaflets on self-care as well as those who were actively triaged. If the former group is excluded, the figures for the two services are similar (14% for NHS111 and 15.6% for 'babylon check'[22].

In their study of self-assessment for students attending a university health centre, Poote et al. found that the prototype system they studied was able to identify a proportion of cases that doctors considered appropriate for self-care, suggesting a potential to reduce service use[17]. Similarly, Little et al's RCT of a web-based symptom checker designed to support self-care for respiratory symptoms[12] reported that patients in the intervention group had fewer contacts with doctors than the usual care control group despite having a longer duration of illness and more days with relatively severe symptoms. This was balanced by an increase in contacts with the NHS Direct telephone service (which preceded NHS 111) and it should be noted that the system under evaluation recommended people needing treatment to contact NHS Direct rather than go directly to a doctor. Finally, a study of young adults (students) found that intention to seek treatment for a hypothetical illness was stronger when the diagnosis was made with the aid of WebMD or Google than with no electronic aid[30].

Patient compliance with triage advice

Only two of the included studies reported specifically on patients' compliance (or intention to comply) with advice received. The NHS England pilot evaluation in four regions asked participants in two of those regions (Suffolk and London) what they intended to do based on the advice received[7]. No quantitative data were provided but the report stated that in the

For peer review only - http://bmjopen.bmj.com/site/about/guidelines.xhtml

Suffolk pilot, 'overall users would have followed the advice given'. However, those who were recommended to call 999 or attend an ED were more likely to seek advice from primary care or self-management. Similarly, in the London region there was generally good agreement between advice and intended action but patients recommended to call 999 or go to an ED indicated that they would seek advice from a GP. In a study of a web-based triage system in the Netherlands, 192 patients were asked about their intention to comply immediately after receiving advice from the system[19]. Thirty-five patients responded to a follow-up survey on actual compliance, of whom 20 (57%) reported that they had followed the advice. Compliance was correlated with intention to comply, which in turn was correlated with the patient's attitude towards the advice received.

Equity and inclusion

Fourteen studies investigated the outcome of equity and inclusion or compared users and non-users. One study [12]reported that patients who were classed as less deprived were more likely to agree to use "Internet Doctor" than decline participation, although no relationship was found between deprivation and results in this study or between e-Consult use and deprivation in another study [24]. Association between e-consultation use and education levels was explored in a third study. Patients with low to medium levels of education tended to be motivated toward indirect e-consultation (which involves contact with a health professional via e-mail), mainly to reduce uncertainty [26]

Evidence from included studies suggests that users of e-consultation were more likely to be young[7 23-25], employed[19 23 25] and female[7 19 24 25]than non-users. One study also found a significantly larger use by white patients (78%) than other ethnicities[24].

Risk of bias assessment

We assessed risk of bias in the two included RCTs[12 13] using the Cochrane risk of bias tool. Thirteen studies[11 18 19 23 24 26-29 31 33-35] were assessed with the tool for cross-sectional and cohort studies and four (six publications[6 17 20 21 36 37]) with the modified QUADAS tool. Seven grey literature reports and conference abstracts were not formally assessed for risk of bias[7 14-16 25 30 32]. Identified limitations were extracted for all included studies.

For peer review only - http://bmjopen.bmj.com/site/about/guidelines.xhtml

Risk of bias results are presented in Appendix 2. With the possible exception of the two randomised trials, the included studies generally had at least a moderate risk of bias. However, the diverse designs and objectives of the studies made risk of bias difficult to assess in some cases with the available tools. Grey literature reports containing relevant data were included in the review but not formally assessed for risk of bias. Reports prepared by individuals with a commercial interest in a specific system and published without independent peer review[16 25] should be treated with particular caution because of possible conflicts of interest.

For peer review only

Overall strength of evidence assessment/evidence map

The overall strength of evidence for key outcomes is summarised in Table 3. We found relatively strong evidence that the diagnostic accuracy of digital and online symptom checkers tends to be lower than that of health professionals; and that patients who have used these systems generally show high levels of satisfaction (mainly in non-comparative studies). Areas where evidence is lacking or inconsistent include clinical and cost-effectiveness, accuracy of disposition to appropriate services and patient compliance with advice received. For safety, we found no evidence of an increased risk with digital/online systems but the available evidence was weak.

For peer review only - http://bmjopen.bmj.com/site/about/guidelines.xhtml

Table 3: Overall strength of evidence by outcome

Outcome	Relevant studies	Evidence statement	Strength of evidence	Comments
Safety	**=Kellermann 2010[11]**<br>**=Little 2016[12]**<br>**=Middleton 2016[16]**<br>**=Poote 2014[17]**<br>**=Price 2013[21]**<br>Madan 2014[25]	No evidence of a difference in risk between health professionals and symptom checkers	Weaker	Rating changed from stronger based on study numbers and design to weaker because of low numbers of adverse events reported
Clinical effectiveness	**-Little 2016[12]**<br>?Madan 2014[25]	Insufficient evidence to draw any firm conclusions	Very limited	
Costs/cost-effectiveness	+Babylon Health 2017[22]<br>+/-Cowie 2018[24]<br>+Madan 2014[25]	Insufficient evidence to draw any firm conclusions	Inconsistent	
Diagnostic accuracy	?Berry 2016[14]<br>**-Berry 2017[32]**<br>**-Berry 2017[15]**<br>**-Price 2013[21]**<br>?Semigran 2015[6]<br>**-Semigran 2016[20]**<br>**=Sole 2006[18]**	Symptom checkers appear inferior to health professionals in terms of diagnostic accuracy	Stronger	Mainly for specific conditions or pre-prepared vignettes
Disposition accuracy	=Babylon Health 2017[22]<br>**-Berry 2017[15]**<br>=**Middleton 2016[16]**<br>?Nijland 2010[19]<br>**-Poote 2014[17]**<br>+/-Semigran 2015[6]	Inconsistent findings on accuracy of disposition	Inconsistent	Performance variable between different systems

For peer review only - http://bmjopen.bmj.com/site/about/guidelines.xhtml

Outcome	Relevant studies	Evidence statement	Strength of evidence	Comments
	+/-**NHS England 2017**[7]			
Service use/diversion	**?Kellermann 2010**[11]<br>**+/-Little 2016**[12]<br>+/-**Poote 2014**[17]<br>?**Carter 2018**[23]<br>?Cowie 2018[24]<br>+Madan 2014[25]<br>+/- **NHS England 2017**[7]<br>+Babylon Health 2017[22]<br>-Luger 2011[33]	Inconsistent findings on effects on service use	Inconsistent	
Compliance	?Nijland[19]<br>?NHS England 2017[7]	No comparative data on compliance	Very limited	
Patient/carer satisfaction	?Nagykaldi 2010[35]<br>?Nijland[19]<br>?Price 2013[21]<br>+Yardley[13]<br>?Carter 2018[23]<br>?Cowie 2018[24]<br>?Madan 2014[25]<br>?NHS England 2017[7]<br>?Lanseng 2007[29]	Most studies report high rates of patient satisfaction with symptom checkers and e-consultation systems generally	Weaker	Few studies with comparator data

Controlled studies in bold; = means no significant difference in outcomes; + means better outcome with symptom checker; +/- varying results within study; ? results difficult to interpret in comparative terms

For peer review only - http://bmjopen.bmj.com/site/about/guidelines.xhtml

Discussion

Main findings

The literature search identified 29 publications describing 27 studies that met the inclusion criteria. The overall strength of the evidence base varied between outcomes (Table 3), but in absolute terms the evidence is weak, being based largely on observational studies. A substantial component of grey literature of uncertain quality complicates the interpretation of the evidence. Interpretation of the evidence should also take into account risks of bias in individual studies.

We found little evidence to indicate whether or not digital and online symptom checkers are detrimental to patient safety. The studies that reported on the outcome were mostly short-term and involved relatively small samples and hence reported few or no adverse events. Some were limited to people with specific types of symptoms and others recruited from specific population groups not representative of typical users of urgent care services. This body of evidence should therefore be interpreted cautiously and not extrapolated to the possible impact of a nationally available digital urgent care service being used by millions of people annually.

The evidence on patient satisfaction with digital and online systems also had some limitations but these findings appear more likely to be generalisable. Study participants generally expressed high levels of satisfaction, albeit in uncontrolled studies. For example, in the NHS England pilot evaluation, 70–80% of users were satisfied with their experience at each of the pilot sites[7]. This evidence, together with the increasing reliance on digital technology in all areas of life, suggests that any national digital urgent care service may be popular and well-used, although different sections of the population may differ in their degree of engagement (see the discussion of equity and inclusion below)..

Digital and online systems have yet to achieve a high level of accuracy in the diagnosis of specific conditions. This finding applies both to 'general purpose' symptom checkers and to those limited to particular conditions. Although the evidence was classified as relatively strong, several caveats should be applied. Some of the included studies did not recruit

For peer review only - http://bmjopen.bmj.com/site/about/guidelines.xhtml

representative populations and others were based on standardised vignettes rather than real-world data. In addition, studies that compared symptom checkers with health professionals tended to use the doctors' clinical diagnosis as the reference standard, which would bias the comparison in favour of the health professionals.

Accuracy of signposting of patients to the most appropriate level of service is closely related to diagnostic accuracy, but results for this outcome were inconsistent between studies. In general, algorithm-based triage tended to be more risk-averse than that of health professionals, with 85% of respondents being advised to visit their doctor in one study[19]. While there is considerable uncertainty about the magnitude of the effect, a national digital urgent care service could result in considerable numbers of patients receiving inappropriate advice to visit the ED or request an urgent GP appointment. Middleton and colleagues[16] claimed that the 'babylon check' app had a high degree of triage accuracy for vignettes compared with health professionals, but this non-peer-reviewed report requires further validation.

We also found inconsistent evidence on effects on service use. There was some indication that symptom checkers can influence the pattern of service use but the magnitude and direction of the effect varied between studies. Patients' reactions to online triage advice and whether they follow the advice or seek further help or information would have implications for service use but we found limited evidence for this outcome. Preliminary findings from the NHS England evaluation suggest that patients may be more likely to seek further advice for more urgent conditions[7] but further confirmation is required.

Over half of the included studies considered equity and inclusion issues either directly or by comparing users and non-users of digital triage systems. Not surprisingly, studies revealed a clear consensus that younger and more highly educated people are more likely to use these services while older and less educated patients are more likely to prefer telephone or face-to-face contact. This could have implications for health equity if urgent care pathways prioritise (or appear to prioritise) requests originating from digital sources. Problems have arisen in primary care because patients using e-consultation systems to request an appointment following online triage may be seen more quickly than those contacting the practice by telephone.

For peer review only - http://bmjopen.bmj.com/site/about/guidelines.xhtml

Strengths and limitations

This systematic review was undertaken on a short timescale using a relatively large team of experienced researchers, including both methodological and topic experts. We performed a rigorous search of the literature including reference checking and citation searching. Rather than a conventional highly sensitive search (which would have resulted in inefficiencies in the screening process), we combined an initial focused search with subsequent rounds of follow-up searching, including searches for named symptom checker systems. We assessed risk of bias in individual studies using a variety of appropriate checklists as well as summarising the overall strength of evidence for key outcomes (Table 3).

The heterogeneous and descriptive nature of the included studies meant that meta-analysis was not feasible for any of the outcomes of interest. Our narrative synthesis approach used a mixture of description and tabulation to summarise the evidence for each of the pre-specified outcomes of interest. This was a review of published (including non-peer-reviewed) literature and the coverage of systems is not exhaustive; for example, we did not extract data from websites. We also did not carry out any original analyses of raw data even where such data were available. The timing of the review meant that final results of NHS England's pilot evaluation were not available to us. We were able to make use of a draft report that was published online[7] but we acknowledge that the findings of the final evaluation report, when available, will supersede those of the 2017 draft.

The review inclusion criteria were relatively broad and findings from symptom checker systems for specific conditions may not be applicable to more general systems and vice versa. We have also included studies of symptom checkers as part of electronic consultation systems in general practice, which again represents a slightly different setting from a general 'digital 111' service, and this should be kept in mind when interpreting the results.

A systematic review in such a topical area of research will require regular updating to keep track of new studies. For example, Verzantvoort et al.[38] published a study of self-triage using a smartphone app for out-of-hours primary care in the Netherlands shortly after our literature searches were completed. The app was rated highly for clarity and patient satisfaction. Sensitivity and specificity (using nurse telephone triage as reference standard) were 84% and 74% respectively, although diagnostic accuracy was only evaluated in a sample of

For peer review only - http://bmjopen.bmj.com/site/about/guidelines.xhtml

participants (126/4456). Inclusion of this study would not have affected the main conclusions of our review.

Implications for service delivery and research

The implications of this systematic review for service delivery should be considered in the context that a decision has already been taken to introduce a ‘digital 111’ service and the service became available across England by December 2018. Achieving a high level of diagnostic accuracy will be key to the success of a ‘digital 111’ service. Failure to provide an accurate diagnosis may result in outcomes including patient dissatisfaction and unwillingness to use the service again; increased use of other urgent and emergency care services; and possible risks to patient safety (although the cautious approach characteristic of most existing systems may help to mitigate this).

The studies included in the review suggest a high level of uncertainty about the impact of ‘digital 111’ on the urgent care system and the wider healthcare system. Some of these uncertainties can be addressed by research and data collection but the health service may need to respond to short-term increases (or decreases) in demand and/or shifts from one part of the system to another. This may increase pressure on the system, at least in the short-term. In the longer-term, if usage of the 111 telephone service decreases as planned, there may be opportunities to reconfigure the workforce to support the integrated urgent care agenda.

Based on the areas of limited evidence identified by the review, priorities for research (in addition to ongoing collection of data to monitor usage and safety of the ‘digital 111’ service) include studies to compare the performance of different systems directly; rigorous economic evaluations based on real-world data; research to investigate the pathways followed by patients using the service; evaluation of systems designed for childhood illnesses; and investigation of the possible role of behaviour change theory in the development and implementation of symptom checkers. Qualitative research to investigate perceptions of symptom checkers and barriers to their use by people who are less familiar with digital technology would also be of value.

For peer review only - http://bmjopen.bmj.com/site/about/guidelines.xhtml

Ethical approval

Ethical approval was not required for this work

Funding

This report presents independent research funded by the National Institute for Health Research (NIHR) Health Services & Delivery Research Programme (project number HSDR16/47/17). The funding programme approved the review protocol but had no role in the collection, analysis and interpretation of the data, the writing of this paper or the decision to submit the paper for publication. The views and opinions expressed are those of the authors and do not necessarily reflect those of the NHS, the NIHR, NETSCC, the HS&DR programme or the Department of Health.

Data sharing

No new data have been created in the preparation of this report and therefore there is nothing available for access and further sharing. All queries should be submitted to the corresponding author.

References

1. NHS England. NHS 111 minimum data set 2018-19 2018 [Available from: https://www.england.nhs.uk/statistics/statistical-work-areas/nhs-111-minimum-data-set/statistical-work-areas-nhs-111-minimum-data-set-nhs-111-minimum-data-set-2018-19/ accessed 29 October 2018.
2. NHS England. Five year forward view. Leeds: NHS England, 2014.
3. NHS England. Next steps on the NHS Five Year Forward View. Leeds: NHS England, 2017.
4. NHS England. Sustainability and transformation partnerships [Available from: https://www.england.nhs.uk/integratedcare/stps/ accessed March 25 2019.
5. Turner J, O'Cathain A, Knowles E, et al. Impact of the urgent care telephone service NHS 111 pilot sites: a controlled before and after study. *BMJ Open* 2013;3(11):e003451. doi: 10.1136/bmjopen-2013-003451
6. Semigran HL, Linder JA, Gidengil C, et al. Evaluation of symptom checkers for self diagnosis and triage: audit study. *BMJ* 2015;351:h3480.
7. NHS England. NHS111 online evaluation. Leeds: NHS England, 2017.
8. Hoffmann TC, Glasziou PP, Boutron I, et al. Better reporting of interventions: template for intervention description and replication (TIDieR) checklist and guide. *BMJ* 2014;348:g1687. doi: 10.1136/bmj.g1687

For peer review only - http://bmjopen.bmj.com/site/about/guidelines.xhtml

9. Reitsma JB, Rutjes AWS, Whiting P, et al. Chapter 9: Assessing methodological quality. In: Deeks JJ, Bossuyt PM, Gatsonis C, eds. Cochrane Handbook for Systematic Reviews of Diagnostic Test Accuracy Version 100: The Cochrane Collaboration 2009.
10. Baxter S, Johnson M, Chambers D, et al. The effects of integrated care: a systematic review of UK and international evidence. *BMC Health Serv Res* 2018;18(1):350. doi: 10.1186/s12913-018-3161-3
11. Kellermann AL, Isakov AP, Parker R, et al. Web-based self-triage of influenza-like illness during the 2009 H1N1 influenza pandemic. *Annals of Emergency Medicine* 2010;56(3):288-94.e6.
12. Little P, Stuart B, Andreou P, et al. Primary care randomised controlled trial of a tailored interactive website for the self-management of respiratory infections (Internet Doctor).[Erratum appears in BMJ Open. 2017 Mar 21;7(3):e009769corr1; PMID: 28325861]. *BMJ Open* 2016;6(4):e009769.
13. Yardley L, Joseph J, Michie S, et al. Evaluation of a Web-based intervention providing tailored advice for self-management of minor respiratory symptoms: exploratory randomized controlled trial. *Journal of Medical Internet Research* 2010;12(4):e66.
14. Berry AC, Berry BB, Nakshabendi R, et al. Evaluation of Accuracy Between Online Symptom Checkers for Diagnosis of Gastrointestinal Symptoms from MKSAP Clinical Vignette Board Review Questions. *Gastroenterology* 2016;150(4):S849-S50. doi: 10.1016/s0016-5085(16)32869-4
15. Berry AC, Cash BD, Mulekar MS, et al. SYMPTOM CHECKERS VS. DOCTORS, THE ULTIMATE TEST: A PROSPECTIVE STUDY OF PATIENTS PRESENTING WITH ABDOMINAL PAIN. *Gastroenterology* 2017;152(5):S852-S53. doi: 10.1016/s0016-5085(17)32937-2
16. Middleton K, Butt M, Hammerla N, et al. Sorting out symptoms: design and evaluation of the 'babylon check' automated triage system. London: Babylon Health, 2016.
17. Poote AE, French DP, Dale J, et al. A study of automated self-assessment in a primary care student health centre setting. *Journal of Telemedicine & Telecare* 2014;20(3):123-7.
18. Sole ML, Stuart PL, Deichen M. Web-based triage in a college health setting. *Journal of American College Health* 2006;54(5):289-94.
19. Nijland N, Cranen K, Boer H, et al. Patient use and compliance with medical advice delivered by a web-based triage system in primary care. *Journal of Telemedicine and Telecare* 2010;16(1):8-11. doi: 10.1258/jtt.2009.001004
20. Semigran HL, Levine DM, Nundy S, et al. Comparison of Physician and Computer Diagnostic Accuracy. *JAMA Intern Med* 2016;176(12):1860-61. doi: 10.1001/jamainternmed.2016.6001
21. Price RA, Fagbuyi D, Harris R, et al. Feasibility of web-based self-triage by parents of children with influenza-like illness: A cautionary tale. *JAMA Pediatrics* 2013;167(2):112-18.
22. Babylon Health. NHS111 powered by babylon: outcomes evaluation. London: Babylon Health, 2017.
23. Carter M, Fletcher E, Sansom A, et al. Feasibility, acceptability and effectiveness of an online alternative to face-to-face consultation in general practice: a mixed-methods study of webGP in six Devon practices. *BMJ Open* 2018;8(2):e018688.
24. Cowie J, Calveley E, Bowers G, et al. Evaluation of a digital consultation and self-care advice tool in primary care: a multi-methods study. *International Journal of Environmental Research and Public Health* 2018;15(896)
25. Madan A. WebGP: the Virtual general practice. London: Hurley Group, 2014.
26. Nijland N, van Gemert-Pijnen J, Boer H, et al. Increasing the use of e-consultation in primary care: Results of an online survey among non-users of e-consultation. *International Journal of Medical Informatics* 2009;78(10):688-703. doi: 10.1016/j.ijmedinf.2009.06.002
27. Backman AS, Lagerlund M, Svensson T, et al. Use of healthcare information and advice among non-urgent patients visiting emergency department or primary care. *Emergency Medicine Journal* 2012;29(12):1004-06.
28. Joury AU, Alshathri M, Alkhunaizi M, et al. Internet Websites for Chest Pain Symptoms Demonstrate Highly Variable Content and Quality. *Academic Emergency Medicine* 2016;23(10):1146-52.

For peer review only - http://bmjopen.bmj.com/site/about/guidelines.xhtml

29. Lanseng EJ, Andreassen TW. Electronic healthcare: a study of people's readiness and attitude toward performing self-diagnosis. *International Journal of Service Industry Management* 2007;18(3-4):394-417. doi: 10.1108/09564230710778155
30. Luger TM, Suls J. Online health information and intentions to seek healthcare. *Psychosomatic Medicine* 2011;73 (3):A59.
31. North F, Varkey P, Laing B, et al. Are e-health web users looking for different symptom information than callers to triage centers? *Telemedicine Journal & E-Health* 2011;17(1):19-24.
32. Berry AC, Berry NA, Wang B, et al. Symptom checkers versus doctors: A prospective, head-to-head comparison for GERD vs. Non-GERD Cough. *American Journal of Gastroenterology* 2017;112 (Supplement 1):S190. doi: http://dx.doi.org/10.1038/ajg.2017.299
33. Luger TM, Houston TK, Suls J. Older adult experience of online diagnosis: results from a scenario-based think-aloud protocol. *Journal of Medical Internet Research* 2014;16(1):e16.
34. Marco-Ruiz L, Bones E, de la Asuncion E, et al. Combining multivariate statistics and the think-aloud protocol to assess Human-Computer Interaction barriers in symptom checkers. *Journal of Biomedical Informatics* 2017;74:104-22.
35. Nagykaldi Z, Calmbach W, Dealleaume L, et al. Facilitating patient self-management through telephony and web technologies in seasonal influenza. *Informatics in Primary Care* 2010;18(1):9-16.
36. Fraser HSF, Clamp S, Wilson CJ. Limitations of Study on Symptom Checkers. *JAMA Internal Medicine* 2017;177(5):740-41.
37. Mehrotra A, Semigran HL, Levine DM, et al. Limitations of Study on Symptom Checkers. *JAMA Internal Medicine* 2017;177(5):741-41.
38. Verzantvoort NCM, Teunis T, Verheij TJM, et al. Self-triage for acute primary care via a smartphone application: Practical, safe and efficient? *PLoS One* 2018;13(6):e0199284. doi: 10.1371/journal.pone.0199284 [published Online First: 2018/06/27]

.

For peer review only - http://bmjopen.bmj.com/site/about/guidelines.xhtml

PRISMA 2009 Flow Diagram

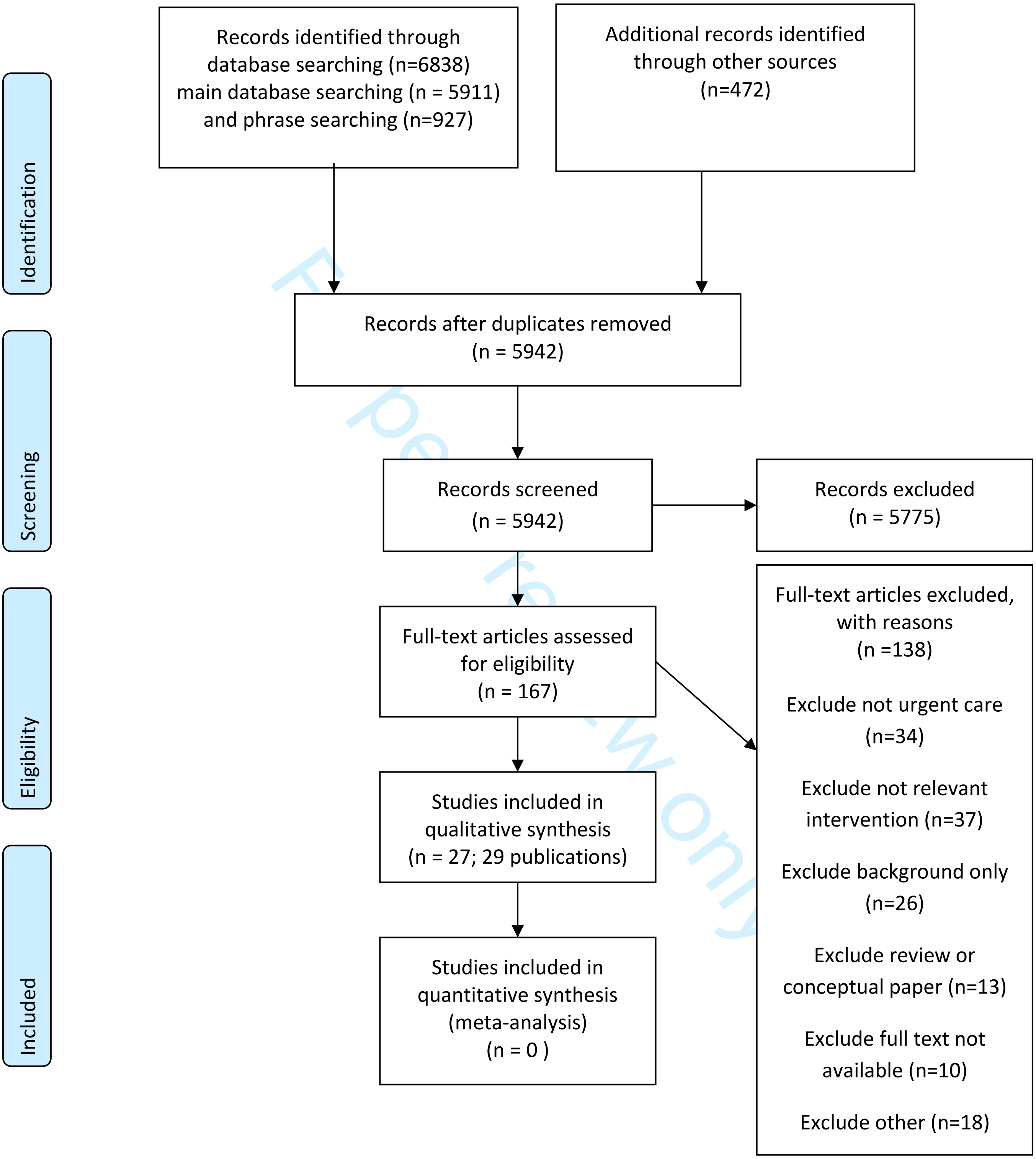

For peer review only

Appendix 1: Database search strategies

Database: Ovid MEDLINE(R) Epub Ahead of Print, In-Process & Other Non-Indexed Citations, Ovid MEDLINE(R) Daily and Ovid MEDLINE(R) <1946 to Present>
Search Strategy:
----
1 (symptom checker or symptoms checker or symptom checkers or symptoms checkers).tw. (21)
2 ("self diagnosis" or "self referral" or "self triage" or "self assessment").tw. (10438)
3 TRIAGE/ (10017)
4 2 or 3 (20415)
5 (online or on-line or web or electronic or automated or internet or digital or app or mobile or smartphone).tw. (658190)
6 4 and 5 (1568)
7 ("online diagnosis" or "web based triage" or "electronic triage" or etriage).tw. (42)
8 1 or 6 or 7 (1608)

****

Embase

----
1 (symptom checker or symptoms checker or symptom checkers or symptoms checkers).tw.
2 ("self diagnosis" or "self referral" or "self triage" or "self assessment").tw.
3 emergency health service/
4 2 or 3
5 (online or on-line or web or electronic or automated or internet or digital or app or mobile or smartphone).tw.
6 4 and 5
7 ("online diagnosis" or "web based triage" or "electronic triage" or etriage).mp. [mp=title, abstract, heading word, drug trade name, original title, device manufacturer, drug manufacturer, device trade name, keyword, floating subheading word, candidate term word]
8 1 or 6 or 7

Cochrane Library

#1 symptom checker or symptoms checker or symptom checkers or symptoms checkers:ti,ab,kw (Word variations have been searched)
#2 "self diagnosis" or "self referral" or "self triage" or "self assessment":ti,ab,kw (Word variations have been searched)
#3 MeSH descriptor: [Triage] explode all trees
#4 #2 or #3
#5 online or on-line or web or electronic or automated or internet or digital or app or mobile or smartphone:ti,ab,kw (Word variations have been searched)
#6 #4 and #5
#7 "online diagnosis" or "web based triage" or "electronic triage" or etriage:ti,ab,kw (Word variations have been searched)
#8 #1 or #6 or #7

CINAHL

S8 (S1 OR S6 OR S7)
S7 TI ("online diagnosis" or "web based triage" or "electronic triage" or etriage) OR AB ("online diagnosis" or "web based triage" or "electronic triage" or etriage)
S6 S4 AND S5

For peer review only

S5 TI (online or on-line or web or electronic or automated or internet or digital or app or mobile or smartphone) OR AB (online or on-line or web or electronic or automated or internet or digital or app or mobile or smartphone)
S4 (S2 OR S3)
S3 (MH "Triage")
S2 TI ("self diagnosis" or "self referral" or "self triage" or "self assessment") OR AB ("self diagnosis" or "self referral" or "self triage" or "self assessment")
S1 TI (symptom checker or symptoms checker or symptom checkers or symptoms checkers) OR AB (symptom checker or symptoms checker or symptom checkers or symptoms checkers)

ACM digital library

WOS
#8 #7 OR #6 OR #1
#7 TS=("online diagnosis" OR "web based triage" OR "electronic triage" OR etriage)
#6 #5 AND #4
#5 TS=(online OR on-line OR web OR electronic OR automated OR internet OR digital OR app OR mobile OR smartphone)
#4 #3 OR #2
#3 TS=triage
#2 TS=("self diagnosis" or "self referral" or "self triage" or "self assessment")
#1 (symptom checker or symptoms checker or symptom checkers or symptoms checkers)

HMIC

1 (symptom checker OR symptoms checker OR symptom checkers OR symptoms checkers).ti,ab
2 ("self diagnosis" OR "self referral" OR "self triage" OR "self assessment").ti,ab
3 TRIAGE/
4 (2 OR 3)
5 (online OR on-line OR web OR electronic OR automated OR internet OR digital OR app OR mobile OR smartphone).ti,ab
6 (4 AND 5)
7 ("online diagnosis" OR "web based triage" OR "electronic triage" OR etriage).ti,ab
8 (1 OR 6 OR 7)

For peer review only

Appendix 2: Risk of bias tables

Risk of bias results for randomised trials

Short Title	Reference	Selection and performance bias	Detection and attrition bias	Reporting and other bias
Little (2016)	**Study ID**<br>• Reference<br>*Little 2016*[12]	**Random sequence generation**<br>• Low risk<br><br>**Allocation concealment**<br>• Low risk<br><br>**Blinding of participants and personnel***<br>• Unclear	**Blinding of outcome assessment***<br>• Low risk<br>*Blinded assessment of primary care records*<br><br>**Incomplete outcome data***<br>• Low risk	**Selective reporting**<br>• Unclear<br><br>**Anything else, ideally prespecified**<br>• Low risk
Yardley (2010)	**Study ID**<br>• Reference<br>*Yardley 2010*[13]	**Random sequence generation**<br>• Low risk<br><br>**Allocation concealment**<br>• Low risk<br><br>**Blinding of participants and personnel***<br>• Low risk	**Blinding of outcome assessment***<br>• Unclear<br><br>**Incomplete outcome data***<br>• Low risk	**Selective reporting**<br>• Unclear<br><br>**Anything else, ideally prespecified**<br>• Low risk

For peer review only

Risk of bias results for cohort/cross-sectional studies

Reference	Questions 1-4	Questions 5-7	Questions 8-10
• Reference<br>*Backman A-S et al. 2012*[30]	**1. Was the research question clearly stated?**<br>• Yes<br>*The aims refer to "non-urgent" but the information is sought prior to visiting ED.*<br><br>**2. Was the study population clearly specified and defined?**<br>• Yes<br><br>**3. Was the participation rate at least 50%?**<br>• Yes<br>*79%*<br><br>**4. Were all the subjects selected or recruited from the same or similar populations?**<br>• Yes<br>*Primary care and ED attendees*	**5. Was a sample size justification provided?**<br>• No<br><br>**6. Did the study examine exposure levels?**<br>• Yes<br>*Health advice seeking*<br><br>**7. Were exposure measures clearly defined?**<br>• Unclear<br>*Measures are vague, e.g. "previous use" of information Also, discriminating between types of information*	**8. Were outcome measures clearly defined?**<br>• Unclear<br>*"Health care information use in the past"*<br><br>**9. Were outcome assessors blinded?**<br>• Not applicable<br><br>**10. Were confounders adjusted for?**<br>• Yes<br>*To some extent: participant and physician attributes assessed for influence on the results.*
• Reference<br>*Carter 2018* [26]	**1. Was the research question clearly stated?**<br>• Yes	**5. Was a sample size justification provided?**<br>• No	**8. Were outcome measures clearly defined?**<br>• Yes<br>*Attitudes and experiences of practice staff and*

For peer review only

	**2. Was the study population clearly specified and defined?**<br>• Yes<br>*GPs, practice staff and their patients at 6 practices in Devon*<br><br>**3. Was the participation rate at least 50%?**<br>• No<br>*Postal survey only had response rate of 35.1% but also GPs judgement of webGP requests and 5GPs and 5 administrators were interviewed.*<br><br>**4. Were all the subjects selected or recruited from the same or similar populations?**<br>• Yes<br>*GPs, practice staff and their patients at 6 practices in Devon*	**6. Did the study examine exposure levels?**<br>• Not applicable<br><br>**7. Were exposure measures clearly defined?**<br>• Not applicable	*patients on webGP.*<br><br>**9. Were outcome assessors blinded?**<br>• Not applicable<br><br>**10. Were confounders adjusted for?**<br>• Not applicable
• Reference *Cowie 2018*[27]	**1. Was the research question clearly stated?**<br>• Yes<br><br>**2. Was the study population clearly specified and defined?**<br>• Yes	**5. Was a sample size justification provided?**<br>• No<br><br>**6. Did the study examine exposure levels?**<br>• No	**8. Were outcome measures clearly defined?**<br>• Yes<br><br>**9. Were outcome assessors blinded?**<br>• No<br><br>**10. Were confounders adjusted for?**

For peer review only

	**3. Was the participation rate at least 50%?**<br>• No<br>*No for patient surveys*<br><br>**4. Were all the subjects selected or recruited from the same or similar populations?**<br>• Yes	**7. Were exposure measures clearly defined?**<br>• Not applicable	• Yes
• Reference<br>*Joury et al. 2016 US*[31]	**1. Was the research question clearly stated?**<br>• Yes<br><br>**2. Was the study population clearly specified and defined?**<br>• Not applicable<br><br>**3. Was the participation rate at least 50%?**<br>• Not applicable<br><br>**4. Were all the subjects selected or recruited from the same or similar populations?**<br>• Not applicable	**5. Was a sample size justification provided?**<br>• No<br><br>**6. Did the study examine exposure levels?**<br>• Not applicable<br><br>**7. Were exposure measures clearly defined?**<br>• Not applicable	**8. Were outcome measures clearly defined?**<br>• Yes<br>*Scores used for readability, popularity, content and quality*<br><br>**9. Were outcome assessors blinded?**<br>• Not applicable<br><br>**10. Were confounders adjusted for?**<br>• Unclear
• Reference<br>*Kellermann 2010* [11]	**1. Was the research question clearly stated?**<br>• Unclear<br><br>**2. Was the study population clearly specified and**	**5. Was a sample size justification provided?**<br>• Not applicable	**8. Were outcome measures clearly defined?**<br>• Not applicable<br><br>**9. Were outcome assessors blinded?**

For peer review only

	**defined?**<br>• Unclear<br>*Patients with influenza-like illness in US that accessed one of 2 websites http://www.flu.gov and www.H1N2 ResponseCenter.com*<br><br>**3. Was the participation rate at least 50%?**<br>• Not applicable<br><br>**4. Were all the subjects selected or recruited from the same or similar populations?**<br>• Unclear<br>*Only counted web hits, no demographic data available on patients. No data on usage of algorithm by clinicians or call centers.*	**6. Did the study examine exposure levels?**<br>• Not applicable<br><br>**7. Were exposure measures clearly defined?**<br>• Not applicable	• Not applicable<br><br>**10. Were confounders adjusted for?**<br>• Not applicable
• Reference<br>*Lanseng & Andreassen 2007 Norway*[32]	**1. Was the research question clearly stated?**<br>• Yes<br><br>**2. Was the study population clearly specified and defined?**<br>• Yes<br><br>**3. Was the participation rate at least 50%?**<br>• Unclear	**5. Was a sample size justification provided?**<br>• No<br><br>**6. Did the study examine exposure levels?**<br>• No<br>*Readiness*<br><br>**7. Were exposure**	**8. Were outcome measures clearly defined?**<br>• Yes<br>*Use of TRI*<br><br>**9. Were outcome assessors blinded?**<br>• No<br><br>**10. Were confounders adjusted for?**<br>• Unclear

For peer review only

	**4. Were all the subjects selected or recruited from the same or similar populations?**<br>• Yes	**measures clearly defined?**<br>• Not applicable	
• Reference *Luger et al. 2014*[23]	**1. Was the research question clearly stated?**<br>• Yes<br><br>**2. Was the study population clearly specified and defined?**<br>• Yes<br><br>**3. Was the participation rate at least 50%?**<br>• Unclear<br><br>**4. Were all the subjects selected or recruited from the same or similar populations?**<br>• Yes	**5. Was a sample size justification provided?**<br>• No<br><br>**6. Did the study examine exposure levels?**<br>• No<br><br>**7. Were exposure measures clearly defined?**<br>• Not applicable	**8. Were outcome measures clearly defined?**<br>• Yes<br><br>**9. Were outcome assessors blinded?**<br>• Not applicable<br><br>**10. Were confounders adjusted for?**<br>• Unclear
• Reference *Marco-Ruiz et al. 2017 Norway*[24]	**1. Was the research question clearly stated?**<br>• Yes<br><br>**2. Was the study population clearly specified and defined?**<br>• No<br><br>**3. Was the participation rate at least 50%?**	**5. Was a sample size justification provided?**<br>• No<br><br>**6. Did the study examine exposure levels?**<br>• No	**8. Were outcome measures clearly defined?**<br>• Not applicable<br><br>**9. Were outcome assessors blinded?**<br>• Not applicable<br><br>**10. Were confounders adjusted for?**<br>• Unclear

For peer review only

	• Yes<br>*53%*<br><br>**4. Were all the subjects selected or recruited from the same or similar populations?**<br>• Unclear	**7. Were exposure measures clearly defined?**<br>• Not applicable	
• Reference<br>*Nagykaldi 2010* [25]	**1. Was the research question clearly stated?**<br>• Yes<br><br>**2. Was the study population clearly specified and defined?**<br>• Yes<br>*Study population was patients from 12 primary care practices in US.*<br><br>**3. Was the participation rate at least 50%?**<br>• Not applicable<br><br>**4. Were all the subjects selected or recruited from the same or similar populations?**<br>• Yes<br>*All participants were patients from 12 primary care practices that accessed customised practice website or telephone helpline*	**5. Was a sample size justification provided?**<br>• Not applicable<br><br>**6. Did the study examine exposure levels?**<br>• Not applicable<br><br>**7. Were exposure measures clearly defined?**<br>• Not applicable	**8. Were outcome measures clearly defined?**<br>• Yes<br>*Web hits on customised practice website influenza self-management webpages. Downloads of self-management influeza toolkit. Completion of Iflueza self-triage module sessions. Volume of calls to telephone hotlines. Qualitative feedback from patients on statisfaction with and utility of self-management websites and telephone hotline. Qualitative feedback from clinicians around their involvement and their perceptionsof patient self-management techniques.*<br><br>**9. Were outcome assessors blinded?**<br>• Not applicable<br><br>**10. Were confounders adjusted for?**<br>• Not applicable

• Reference *Nijland 2009*[29]	**1. Was the research question clearly stated?**<br>• Yes<br><br>**2. Was the study population clearly specified and defined?**<br>• Yes<br><br>**3. Was the participation rate at least 50%?**<br>• Unclear<br><br>**4. Were all the subjects selected or recruited from the same or similar populations?**<br>• Yes	**5. Was a sample size justification provided?**<br>• No<br><br>**6. Did the study examine exposure levels?**<br>• Not applicable<br><br>**7. Were exposure measures clearly defined?**<br>• Not applicable	**8. Were outcome measures clearly defined?**<br>• Yes<br><br>**9. Were outcome assessors blinded?**<br>• No<br><br>**10. Were confounders adjusted for?**<br>• Yes<br>*Methods not very clearly reported but appears to be multiple regression*
• Reference *Nijland 2016*[19]	**1. Was the research question clearly stated?**<br>• Yes<br><br>**2. Was the study population clearly specified and defined?**<br>• Yes<br><br>**3. Was the participation rate at least 50%?**<br>• No<br>*Low participation rate in survey relative to users of triage system (though unclear how many were invited to participate)*	**5. Was a sample size justification provided?**<br>• No<br><br>**6. Did the study examine exposure levels?**<br>• Not applicable<br><br>**7. Were exposure measures clearly defined?**	**8. Were outcome measures clearly defined?**<br>• Yes<br><br>**9. Were outcome assessors blinded?**<br>• No<br><br>**10. Were confounders adjusted for?**<br>• Unclear

For peer review only

	**4. Were all the subjects selected or recruited from the same or similar populations?**<br>• Yes	• Not applicable	
• Reference<br>*North et. al. 2011*[34]	**1. Was the research question clearly stated?**<br>• Yes<br><br>**2. Was the study population clearly specified and defined?**<br>• Yes<br><br>**3. Was the participation rate at least 50%?**<br>• Not applicable<br><br>**4. Were all the subjects selected or recruited from the same or similar populations?**<br>• Not applicable	**5. Was a sample size justification provided?**<br>• Not applicable<br><br>**6. Did the study examine exposure levels?**<br>• Yes<br>*Self-exposure*<br><br>**7. Were exposure measures clearly defined?**<br>• Not applicable	**8. Were outcome measures clearly defined?**<br>• Yes<br><br>**9. Were outcome assessors blinded?**<br>• Not applicable<br><br>**10. Were confounders adjusted for?**<br>• Unclear<br>*Some discussion of potential confounders.*
• Reference<br>*Sole 2006*[18]	**1. Was the research question clearly stated?**<br>• Yes<br>*"The primary purpose of this study was to identify and describe the demographic profile of students who used the newly implemented Web-based triage system. A secondary purpose was to compare Web-based triage diagnoses to the diagnoses made in clinic for a subset*	**5. Was a sample size justification provided?**<br>• No<br><br>**6. Did the study examine exposure**	**8. Were outcome measures clearly defined?**<br>• Not applicable<br><br>**9. Were outcome assessors blinded?**<br>• Not applicable

For peer review only

	of students who requested appointments"<br><br>**2. Was the study population clearly specified and defined?**<br>• Yes<br>*Students who used the web based triage over a four month implementation period (1290 students). Then of those students, those who requested an appointment via email (143 students), then of those 59 who attended the health centre after requesting an email appointment.*<br><br>**3. Was the participation rate at least 50%?**<br>• Not applicable<br><br>**4. Were all the subjects selected or recruited from the same or similar populations?**<br>• Yes	**levels?**<br>• Yes<br><br>**7. Were exposure measures clearly defined?**<br>• Yes	**10. Were confounders adjusted for?**<br>• Not applicable

Risk of bias results for diagnostic studies

Reference	Questions 1 to 4	Questions 5 to 8	Questions 9 to 11
**Study ID**<br>•<br>Reference<br>*Poote*	**1. Representative spectrum?**<br>• No<br>*Study participants were all patients registered at a student health centre in England attending with new acute*	**5. Differential verification avoided?**<br>• Not applicable?	**9. Relevant clinical information?**<br>• Yes<br><br>**10. Were uninterpretable results reported?**

For peer review only

2014 [17]	*symptoms. If the self-assessment triage system was only for students to be representative the study population would have needed to include range of student health centres in different areas. If the system was for any UK general practices the study population would have needed to include patients of all ages, ethnicity, gender etc from a range GP practices in different areas.*<br><br>**2. Acceptable reference standard?**<br>• Yes<br><br>**3. Acceptable delay between tests?**<br>• Yes<br><br>**4. Partial verification avoided?**<br>• Yes<br>*All patients that completed self-triage also had a GP consultation where the GP rated the urgency of their consultation.*	**6. Was the reference standard independent of the index test?**<br>• Unclear<br>*Patients took the assessment from self-triage through to their GP consultation.*<br><br>**7. Index test results blinded?**<br>• No<br>*Patients took the assessment from self-triage through to their GP consultation.*<br><br>**8. Reference standard results blinded?**<br>• Yes	• Not applicable<br><br>**11. Were withdrawals from the study explained?**<br>• Yes
**Study ID**	**1. Representative spectrum?**	**5. Differential**	**9. Relevant clinical information?**

For peer review only

•<br>Reference<br>*Price 2013* [20]	• No<br>*SORT was only trialled in 2 Emergency Departments in US, a larger range would be needed for a representative spectrum. Also, patients were from ED not home so potentially sicker patients in the sample.*<br><br>**2. Acceptable reference standard?**<br>• Yes<br>*Sensitivity of SORT for kids algorithm in identifying the need for ED care was based on an explicit gold standard: documented evidence that the child received 1 or more of 5 ED-specific interventions.*<br><br>**3. Acceptable delay between tests?**<br>• Yes<br><br>**4. Partial verification avoided?**<br>• Yes	**verification avoided?**<br>• Not applicable?<br><br>**6. Was the reference standard independent of the index test?**<br>• Yes<br><br>**7. Index test results blinded?**<br>• Yes<br><br>**8. Reference standard results blinded?**<br>• Yes	• Yes<br><br>**10. Were uninterpretable results reported?**<br>• Not applicable<br><br>**11. Were withdrawals from the study explained?**<br>• No
**Study ID**<br>•<br>Reference<br>*Semigran 2015* [4]	**1. Representative spectrum?**<br>• Unclear<br>*There were 45 standardised patient vignettes which were divided into three levels of triage urgency and included more and less common conditions. It is not clear how closely this replicates the spectrum of conditions that people use symptom checkers for.*	**5. Differential verification avoided?**<br>• Not applicable?<br><br>**6. Was the reference standard independent of the**	**9. Relevant clinical information?**<br>• Yes<br>*This is the clinical information that would be supplied by the patient which may or may not differ from the information given by the vignette.*<br>[#548 Semigran 2015.pdf] Page 8: *ion of the true clinical accuracy of symptom checkers.33 Some standardized patient vignettes con- tained specifc clinical language (for*

	**2. Acceptable reference standard?**<br>• Yes<br>[#548 Semigran 2015.pdf] Page 2: *The source for each vignette also provided the associated correct diagnosis.*<br><br>**3. Acceptable delay between tests?**<br>• Not applicable<br><br>**4. Partial verification avoided?**<br>• Not applicable	**index test?**<br>• Yes<br><br>**7. Index test results blinded?**<br>• Yes<br><br>**8. Reference standard results blinded?**<br>• Yes	*example, mouth ulcers, tonsils with exudate), and actual patients with the same condition might struggle with the words to use to describe their symptoms or use diferent terms. Therefore, our analysis represents an indirect assess- ment of how well symptom checkers would perform with actual patients*<br><br>**10. Were uninterpretable results reported?**<br>• Yes<br>[#548 Semigran 2015.pdf] Page 3: *ns for diagnosis and triage was high (Cohen's κ 0.90). In some cases we could not evaluate a vignette because some symptom checkers focus only on children or on adults or the symptom checker did not list or ask for the key symp- tom in the vignette. To avoid penalizing these symptom checkers, we referred to standardized patient vignettes that successfully yielded an output as "standardized patient evaluations."*<br><br>**11. Were withdrawals from the study explained?**<br>• Not applicable
**Study ID**<br>•<br>Reference *Semigran 2016* [8]	**1. Representative spectrum?**<br>• Unclear<br>*There were 45 standardised patient vignettes which were divided into three levels of triage urgency and included more and less common conditions. It is not clear how closely this replicates the spectrum of conditions that people use symptom checkers for.*	**5. Differential verification avoided?**<br>• Not applicable?<br><br>**6. Was the**	**9. Relevant clinical information?**<br>• Yes<br>*The physicians and the symptom checkers used the same vignettes*<br><br>**10. Were uninterpretable results reported?**

For peer review only

	**2. Acceptable reference standard?**<br>• Yes<br><br>**3. Acceptable delay between tests?**<br>• Not applicable<br><br>**4. Partial verification avoided?**<br>• No<br>*There was a total of 234 physicians involved in the study and of the 45 vignettes, each was solved by at least 20 physicians but it is not clear why they chose the specific vignettes to solve.*	**reference standard independent of the index test?**<br>• Not applicable<br><br>**7. Index test results blinded?**<br>• Yes<br><br>**8. Reference standard results blinded?**<br>• Yes	• Not applicable<br><br>**11. Were withdrawals from the study explained?**<br>• No<br>*It is unclear why the physicians chose to solve the specific vignettes*

For peer review only

PRISMA 2009 Checklist

Section/topic	#	Checklist item	Reported on page #
**TITLE**			
Title	1	Identify the report as a systematic review, meta-analysis, or both.	1
**ABSTRACT**			
Structured summary	2	Provide a structured summary including, as applicable: background; objectives; data sources; study eligibility criteria, participants, and interventions; study appraisal and synthesis methods; results; limitations; conclusions and implications of key findings; systematic review registration number.	2-3
**INTRODUCTION**			
Rationale	3	Describe the rationale for the review in the context of what is already known.	4-5
Objectives	4	Provide an explicit statement of questions being addressed with reference to participants, interventions, comparisons, outcomes, and study design (PICOS).	5
**METHODS**			
Protocol and registration	5	Indicate if a review protocol exists, if and where it can be accessed (e.g., Web address), and, if available, provide registration information including registration number.	5
Eligibility criteria	6	Specify study characteristics (e.g., PICOS, length of follow-up) and report characteristics (e.g., years considered, language, publication status) used as criteria for eligibility, giving rationale.	6
Information sources	7	Describe all information sources (e.g., databases with dates of coverage, contact with study authors to identify additional studies) in the search and date last searched.	5-6
Search	8	Present full electronic search strategy for at least one database, including any limits used, such that it could be repeated.	Appendix 1
Study selection	9	State the process for selecting studies (i.e., screening, eligibility, included in systematic review, and, if applicable, included in the meta-analysis).	6
Data collection process	10	Describe method of data extraction from reports (e.g., piloted forms, independently, in duplicate) and any processes for obtaining and confirming data from investigators.	7
Data items	11	List and define all variables for which data were sought (e.g., PICOS, funding sources) and any assumptions and simplifications made.	7
Risk of bias in individual studies	12	Describe methods used for assessing risk of bias of individual studies (including specification of whether this was done at the study or outcome level), and how this information is to be used in any data synthesis.	7
Summary measures	13	State the principal summary measures (e.g., risk ratio, difference in means).	N/A
Synthesis of results	14	Describe the methods of handling data and combining results of studies, if done, including measures of consistency (e.g., I^2) for each meta-analysis.	N/A

For peer review only - http://bmjopen.bmj.com/site/about/guidelines.xhtml

PRISMA 2009 Checklist

Section/topic	#	Checklist item	Reported on page #
Risk of bias across studies	15	Specify any assessment of risk of bias that may affect the cumulative evidence (e.g., publication bias, selective reporting within studies).	N/A
Additional analyses	16	Describe methods of additional analyses (e.g., sensitivity or subgroup analyses, meta-regression), if done, indicating which were pre-specified.	7
**RESULTS**			
Study selection	17	Give numbers of studies screened, assessed for eligibility, and included in the review, with reasons for exclusions at each stage, ideally with a flow diagram.	9
Study characteristics	18	For each study, present characteristics for which data were extracted (e.g., study size, PICOS, follow-up period) and provide the citations.	10-16
Risk of bias within studies	19	Present data on risk of bias of each study and, if available, any outcome level assessment (see item 12).	Appendix 2
Results of individual studies	20	For all outcomes considered (benefits or harms), present, for each study: (a) simple summary data for each intervention group (b) effect estimates and confidence intervals, ideally with a forest plot.	17-22
Synthesis of results	21	Present results of each meta-analysis done, including confidence intervals and measures of consistency.	N/A
Risk of bias across studies	22	Present results of any assessment of risk of bias across studies (see Item 15).	N/A
Additional analysis	23	Give results of additional analyses, if done (e.g., sensitivity or subgroup analyses, meta-regression [see Item 16]).	22-23
**DISCUSSION**			
Summary of evidence	24	Summarize the main findings including the strength of evidence for each main outcome; consider their relevance to key groups (e.g., healthcare providers, users, and policy makers).	26-27
Limitations	25	Discuss limitations at study and outcome level (e.g., risk of bias), and at review-level (e.g., incomplete retrieval of identified research, reporting bias).	28
Conclusions	26	Provide a general interpretation of the results in the context of other evidence, and implications for future research.	28-29
**FUNDING**			
Funding	27	Describe sources of funding for the systematic review and other support (e.g., supply of data); role of funders for the systematic review.	29

From: Moher D, Liberati A, Tetzlaff J, Altman DG, The PRISMA Group (2009). Preferred Reporting Items for Systematic Reviews and Meta-Analyses: The PRISMA Statement. PLoS Med 6(7): e1000097. doi:10.1371/journal.pmed1000097

For more information, visit: **www.prisma-statement.org**.

For peer review only - http://bmjopen.bmj.com/site/about/guidelines.xhtml

The PRISMA for Abstracts Checklist

TITLE	CHECKLIST ITEM	REPORTED ON PAGE #
1. Title:	Identify the report as a systematic review, meta-analysis, or both.	1 (also in 'Design')
**BACKGROUND**		
2. Objectives:	The research question including components such as participants, interventions, comparators, and outcomes.	3 (Objectives)
**METHODS**		
3. Eligibility criteria:	Study and report characteristics used as criteria for inclusion.	3 (Eligibility criteria)
4. Information sources:	Key databases searched and search dates.	3 (Data sources)
5. Risk of bias:	Methods of assessing risk of bias.	3 (DE and synthesis)
**RESULTS**		
6. Included studies:	Number and type of included studies and participants and relevant characteristics of studies.	3 (Results)
7. Synthesis of results:	Results for main outcomes (benefits and harms), preferably indicating the number of studies and participants for each. If meta-analysis was done, include summary measures and confidence intervals.	3 (Results)
8. Description of the effect:	Direction of the effect (i.e. which group is favoured) and size of the effect in terms meaningful to clinicians and patients.	3 (Results)
**DISCUSSION**		
9. Strengths and Limitations of evidence:	Brief summary of strengths and limitations of evidence (e.g. inconsistency, imprecision, indirectness, or risk of bias, other supporting or conflicting evidence)	3 (Results)
10. Interpretation:	General interpretation of the results and important implications	3 (Conclusions)

**OTHER**		
11. Funding:	Primary source of funding for the review.	In text
12. Registration:	Registration number and registry name.	3

For peer review only

BMJ Open

Digital and online symptom checkers and health assessment/triage services for urgent health problems: systematic review

Journal:	*BMJ Open*
Manuscript ID	bmjopen-2018-027743.R2
Article Type:	Research
Date Submitted by the Author:	12-Jun-2019
Complete List of Authors:	Chambers, Duncan ; The University of Sheffield, ScHARR<br>Cantrell, Anna; The University of Sheffield, ScHARR<br>Johnson, Maxine; The University of Sheffield, ScHARR<br>Preston, Louise; The University of Sheffield, ScHARR<br>Baxter, Susan; The University of Sheffield, ScHARR<br>Booth, Andrew; The University of Sheffield, ScHARR<br>Turner, Janette; The University of Sheffield, ScHARR
**Primary Subject Heading**:	Health services research
Secondary Subject Heading:	Diagnostics
Keywords:	urgent care, symptom checkers, systematic reviews

SCHOLARONE™
Manuscripts

Digital and online symptom checkers and health assessment/triage services for urgent health problems: systematic review

Duncan Chambers[1], Anna Cantrell[1], Maxine Johnson[1], Louise Preston[1], Susan K Baxter[1], Andrew Booth[1] and Janette Turner[1]

[1]School of Health and Related Research (ScHARR), University of Sheffield, Regent Court, Sheffield S1 4DA, UK

*Correspondence to Duncan Chambers: d.chambers@sheffield.ac.uk

Contributor/guarantor information:

DC contributed to the planning (project co-ordination and protocol development), conduct (study selection, data extraction and quality assessment) and reporting (report writing) of the study. AC contributed to the planning (protocol development), conduct (information retrieval, study selection, data extraction and quality assessment) and reporting (report writing) of the study. MJ contributed to the planning (protocol development), conduct (study selection, data extraction and quality assessment) and reporting (report writing) of the study. LP contributed to the planning (protocol development), conduct (study selection, data extraction and quality assessment) and reporting (report writing) of the study. SB contributed to the planning (protocol development), conduct (study selection, data extraction and quality assessment) and reporting (report writing) of the study. AB contributed to the planning (protocol development), conduct (information retrieval and study selection) and reporting (report writing) of the study. JT contributed to the planning, conduct and reporting of the study by providing expert topic advice at all stages. All the authors contributed to the study conception and design (protocol development), acquisition of data (study selection and data extraction) and analysis or interpretation of data (writing sections and/or commenting on drafts of the report). Duncan Chambers is the guarantor for this work. The corresponding author attests

For peer review only - http://bmjopen.bmj.com/site/about/guidelines.xhtml

that all listed authors meet authorship criteria and that no others meeting the criteria have been omitted.

Competing interests

None of the authors have any competing interests

Copyright

The Corresponding Author has the right to grant on behalf of all authors and does grant on behalf of all authors, a non-exclusive worldwide licence to the Publishers and its licensees in perpetuity, in all forms, formats and media (whether known now or created in the future), to i) publish, reproduce, distribute, display and store the Contribution, ii) translate the Contribution into other languages, create adaptations, reprints, include within collections and create summaries, extracts and/or, abstracts of the Contribution, iii) create any other derivative work(s) based on the Contribution, iv) to exploit all subsidiary rights in the Contribution, v) the inclusion of electronic links from the Contribution to third party material where-ever it may be located; and, vi) licence any third party to do any or all of the above.

For peer review only

Abstract

Objectives: In England, the NHS111 service provides assessment and triage by telephone for urgent health problems. A digital version of this service has recently been introduced. We aimed to systematically review the evidence on digital and online symptom checkers and similar services.

Design: Systematic review.

Data sources: We searched MEDLINE, EMBASE, the Cochrane Library, CINAHL, HMIC (Health Management Information Consortium), Web of Science and ACM Digital Library up to April 2018, supplemented by phrase searches for known symptom checkers and citation searching of key studies.

Eligibility criteria: Studies of any design that evaluated a digital or online symptom checker or health assessment service for people seeking advice about an urgent health problem.

Data extraction and synthesis: Data extraction and quality assessment (using the Cochrane Collaboration version of QUADAS for diagnostic accuracy studies and the National Heart, Lung and Blood Institute tool for observational studies) -were done by one reviewer with a sample checked for accuracy and consistency. We performed a narrative synthesis of the included studies structured around pre-defined research questions and key outcomes.

Results: We included 29 publications (27 studies). Evidence on patient safety was weak. Diagnostic accuracy varied between different systems but was generally low. Algorithm-based triage tended to be more risk-averse than that of health professionals. There was very limited evidence on patients' compliance with online triage advice. Study participants generally expressed high levels of satisfaction, albeit in mainly uncontrolled studies. Younger and more highly educated people were more likely to use these services.

Conclusions: The English 'digital 111' service has been implemented against a background of uncertainty around the likely impact on important outcomes. The health system may need to respond to short-term changes and/or shifts in demand. The popularity of online and digital services with younger and more educated people has implications for health equity.

Registration: PROSPERO (registration number CRD42018093564)

Strengths and limitations of this study

For peer review only - http://bmjopen.bmj.com/site/about/guidelines.xhtml

- This systematic review was based on a rigorous search of the literature which maximised efficiency by combining an initial focused search with subsequent rounds of follow-up searching, including searches for named symptom checker systems.
- Our narrative synthesis approach used a mixture of description and tabulation to summarise the evidence, including overall strength of the evidence base for each of the pre-specified outcomes of interest.
- Given the decision to implement a national urgent care service based on digital symptom checkers in the NHS in England, our study highlights areas of uncertainty that will need to be resolved by research and data collection.
- The review inclusion criteria were relatively broad and findings from symptom checker systems for specific conditions may not be applicable to more general systems and vice versa.
- We have also included studies of symptom checkers as part of electronic consultation systems in general practice, which again represents a slightly different setting from a general 'digital 111' service, and this should be kept in mind when interpreting the results.

For peer review only

Introduction

Digital and online symptom checkers and assessment services are used by patients seeking guidance about health problems, including some that may require urgent action. These services generally provide people with possible alternative diagnoses based on their reported symptoms and/or suggest a course of action (e.g. self-care, make a GP appointment or go to an emergency department (ED)).

In England, the NHS111 service provides assessment and triage by telephone for problems that are urgent but not classified as emergencies. The latest data from NHS England[1] show that in September2018 there were over 1.27 million calls to NHS111, an average of 42,400 per day. Outcomes of these calls were that 13.2% had ambulances despatched; 9.5% were recommended to attend an ED; 58.7% were recommended to attend primary care; 4.8% to attend another service; and 13.8% were not recommended to attend another service (e.g. their condition was considered suitable for self-care)

NHS England has recently introduced a digital platform to make NHS111accessible via a website or smartphone app. A beta version of the service (referred to as 'NHS111 Online') is available at https://111.nhs.uk/ (accessed 1 April 2019). The 'digital 111' service is seen as key to reducing demand for the telephone 111 service, enabling resources to be redirected to supporting 'integrated urgent and emergency care systems' as outlined in the 'NHS 5-year Forward View' and its 2017 update 'Next Steps on the NHS 5-year Forward View'[2 3].

There is an expectation that a digital 111 platform will help to manage demand and increase efficiency in the urgent and emergency care system, complementing the agenda of locally based Sustainability and Transformation Partnerships (STPs) which involve the health service and local government working together to integrate and co-ordinate care[4]. However, there is a risk of increasing demand, duplicating healthcare contacts (by increasing the number of potential access routes into the system) and providing advice that is not safe or clinically appropriate. For example, an evaluation of the NHS111 telephone service at four pilot sites and three control sites found that in its first year the service was not successful in reducing 999 emergency calls or in shifting patients from emergency to urgent care[5]. A recent study of 23 symptom checker algorithms providing diagnostic and triage advice that would form the

For peer review only - http://bmjopen.bmj.com/site/about/guidelines.xhtml

basis of a ‘digital 111’ platform found deficiencies in both their diagnostic and triage capabilities (based on patient vignettes)[6].

In 2017, NHS England carried out pilot evaluations of different systems in four regions of England. The evaluations aimed to assess whether digital/online triage was acceptable to users and connected them to appropriate clinical care[7]. The full report of the evaluations was not yet published at the time of writing. The objective of this systematic review was to inform further development of the proposed digital platform by summarising and critiquing the previous research in this area, both from the UK and overseas. The overall research question was: for people seeking guidance about an urgent health problem, what is the effect of digital and online services designed to assess symptoms and signpost patients to appropriate services (compared with non-digital services or no comparator) on important clinical and health service outcomes? Outcomes include safety; clinical and cost-effectiveness; diagnostic and triage accuracy; impact on service use; patient/carer satisfaction; compliance with advice received; and outcomes related to equity and inclusion.

Methods

The review protocol was registered with PROSPERO (registration number CRD42018093564) and is available from the project website (https://www.journalslibrary.nihr.ac.uk/programmes/hsdr/164717/).

Literature search and screening

Initial scoping searches revealed that a highly sensitive search strategy, as typically conducted for systematic reviews, retrieved a disproportionately high number of references on GP decision-making and triage as demonstrated by examination of sample search results (e.g. first 100). We therefore devised a three stage retrieval strategy as an acceptable alternative to comprehensive topic-based searching. This involved:

1. Targeted searches of precise high specificity terms in seven databases (MEDLINE, EMBASE, the Cochrane Library, CINAHL, HMIC (Health Management Information Consortium), Web of Science and ACM Digital Library). These searches were not restricted

For peer review only - http://bmjopen.bmj.com/site/about/guidelines.xhtml

by language or date. The search strategies used for this part of the review are presented in Appendix 1.

2. Phrase searching for names of known symptom checkers using a list compiled from Semigran 2015 and other sources

3. Citation searches and reference checking of key included studies and reviews, complemented by contact with service providers (directly and via websites).

The main literature search was completed in April 2018 and follow-up searches in May 2018. Search results were stored in a reference management system (EndNote) and imported into EPPI-Reviewer software for screening, data extraction and quality assessment. The search results were screened against the inclusion criteria by one reviewer, with a 10% random sample screened by a second reviewer. Uncertainties were resolved by discussion among the review team.

Inclusion and exclusion criteria

Population: General population seeking information online or digitally to address an urgent health problem, including adults and children and issues arising from both acute and long-term chronic illness.

Intervention: Any online or digital service designed to assess symptoms, provide health advice and direct patients to appropriate services. Services that only provide health advice were excluded, as were those that offer treatment, e.g. online CBT services.

Comparator: The ‘gold standard’ comparator is current practice of telephone assessment (e.g. NHS111) or face to face assessment (e.g. general practice, urgent care centre or ED). However, studies with other relevant comparators (e.g. comparative performance in tests or simulations) or with no comparator were included if they addressed the research questions.

Outcomes: The main outcomes of interest were safety (e.g. any evidence of adverse events arising from following or ignoring advice from online/digital services); clinical effectiveness; costs/cost-effectiveness; accuracy; impact on service use; compliance with advice received; patient/carer satisfaction; and equity and inclusion. ‘Accuracy covered 1) ability to provide a correct diagnosis and 2) ability to distinguish between high and low acuity/urgency problems (and hence direct patients to appropriate services).

For peer review only - http://bmjopen.bmj.com/site/about/guidelines.xhtml

Study design: We did not restrict inclusion by study design (and included relevant audits or service evaluations in addition to formal research studies) but included studies had to evaluate (quantitatively or qualitatively) some aspect of an online/digital service

Other: Studies from any developed country healthcare system were eligible for inclusion

Excluded: Purely descriptive studies, conceptual papers, projections of possible future developments and studies conducted in low or middle income countries were excluded from the review.

Data extraction and quality/strength of evidence assessment

We extracted and tabulated key data from the included studies, including study design, population/setting, results and key limitations. Data extraction was performed by one reviewer, with a 10% random sample checked for accuracy and consistency.

To characterise the included digital and online systems as interventions, we identified studies reporting on a particular system and extracted data from all relevant studies using a modification of the TIDieR (Template for Intervention Description and Replication) checklist[8] which we designated TIDieST (Template for Intervention Description for Systems for Triage). Further details may be found in the full report (Chambers et al., *Health Services & Delivery Research* 2019 (in press)).

Quality (risk of bias) assessment was undertaken for peer-reviewed full publications only (i.e. not grey literature publications (such as research reports, working papers, or reports produced by government departments, academics, business and industry) or conference abstracts). Randomised controlled trials were assessed using the Cochrane Collaboration risk of bias tool. For diagnostic accuracy type studies, we used the Cochrane Collaboration version of QUADAS[9] and for other study designs we used the National Heart Lung and Blood Institute tool for observational cohort and cross-sectional studies (https://www.nhlbi.nih.gov/health-topics/study-quality-assessment-tools, accessed 25th March 2019). Quality assessment was performed by one reviewer, with a random 10% sample checked for accuracy and consistency.

Assessment of the overall strength (quality and relevance) of evidence for each research question is part of the narrative synthesis. Overall strength of the evidence base for key

For peer review only - http://bmjopen.bmj.com/site/about/guidelines.xhtml

outcomes was assessed using an adaptation of the method described by Baxter et al.[10] This involves classifying evidence as 'stronger', 'weaker', 'conflicting' or 'insufficient' based on study numbers and design. Specifically, "stronger evidence" represented generally consistent findings in multiple studies with a comparator group design or comparative diagnostic accuracy studies; "weaker evidence" represented generally consistent findings in one study with a comparator group design and several non-comparator studies or multiple non-comparator studies; "very limited evidence" represented an outcome reported by a single study; and finally, "inconsistent evidence" represented an outcome where fewer than 75% of studies agreed on the direction of effect. All studies in the review, including those that did not meet criteria for risk of bias assessment, were included in the strength of evidence assessment.

Evidence synthesis

We performed a narrative synthesis structured around the pre-specified research questions and outcomes. We did not perform any meta-analyses because the included studies varied widely in terms of design, methodology and outcomes.

Patient and public involvement (PPI)

The review was discussed at two meetings of an existing PPI group covering the programme from which the review was commissioned (Sheffield HS&DR Evidence Synthesis Centre). At the meetings there was discussion regarding the focus of the work, including a presentation on previous research on NHS111 telephone services to provide a context for understanding the current work. The meetings also included presentation and discussion of the findings of the review, in order to explore key messages for patients which could inform dissemination of the findings. Discussion during one meeting was structured using a SWOT (strengths, weaknesses, opportunities and threats) analysis approach, which revealed a number of potential concerns amongst patients (e.g. reliability and consistency; high costs of programming and development; whether patients would follow advice given; and threats to equity) as well as potential perceived benefits (e.g. improved access to care at all hours; value to those who might feel embarrassed discussing their problem with a health professional). Involvement of the advisory group was beneficial in highlighting some issues that had also emerged from the systematic review, and enabled the reviewers to structure the review findings taking this into account. For example, the group's uncertainty about the likely

For peer review only - http://bmjopen.bmj.com/site/about/guidelines.xhtml

impact of ‘digital 111’ was reflected in the review findings and recommendations for ongoing evaluation and further research. The review report also reflects the group’s relatively cautious attitude (while recognising the need to update the way services are accessed) which contrasts with the strong belief in some quarters that ‘digital 111’ will help to ensure that patients receive appropriate care more quickly while reducing ‘inappropriate’ visits to EDs and GP appointments.

Results

Results of literature search

Twenty-seven studies (29 publications) were included in the review. Figure 1 presents the flow of studies through the selection process. Inter-rater agreement on initial study selection was moderate (Kappa = 0.582). This reflects a degree of learning by the review team: our initial sift of the search results consciously favoured inclusivity and items found not to meet the inclusion criteria on detailed examination were subsequently discarded.

For peer review only

For peer review only - http://bmjopen.bmj.com/site/about/guidelines.xhtml

Figure 1: PRISMA flow diagram

Characteristics of included studies

Seventeen studies (Table 1) evaluated symptom checkers as a self-contained intervention, of which eight covered a limited range of symptoms, e.g. respiratory[11-13] or gastrointestinal[14 15] symptoms which we considered to be 'urgent'. The remaining studies in this group evaluated symptom checkers covering a wider range of common urgent care symptoms. Studies either evaluated a single system[16-19] or multiple systems[6 20]. We found only one study of a symptom checker specifically intended for assessment of children's symptoms, a development of the SORT (Strategy for Off-Site Rapid Triage) system for influenza-like illness[21] Two reports with some overlap of content evaluated the 'babylon check' app[16 22]. Studies were conducted in the USA, UK or other European countries.

Five studies (four from the UK)[7 23-26] evaluated symptom checkers as part of a broader self-assessment and consultation system (often referred to as electronic consultation or e-consultation). Study characteristics are summarised in Table 2. In this type of system, the role of symptom checkers is to help patients decide whether their symptoms require a consultation with a doctor or other health professional or can be dealt with by self-care. If a consultation is required, details of the symptoms and a request for an appointment or call-back can be submitted electronically. This type of study is important because it considers the service within the broader context of the urgent and emergency care system. A limitation is that some studies focused mainly on the 'downstream' elements of the pathway, e.g. consultation with GPs, and provided limited data on the symptom checker element of the system.

A final group of five studies examined patient and/or public attitudes to online self-diagnosis in the context of urgent care[27-31].See the full report for further details (Chambers et al. *Health Services & Delivery Research* 2019 (in press)).

For peer review only - http://bmjopen.bmj.com/site/about/guidelines.xhtml

Table 1: Studies of symptom checkers as a self-contained intervention

Reference	Study design	System type	Comparator	Population/sample
Babylon Health 2017[22] UK	• Uncontrolled observational *No control group but some comparison with NHS111 telephone data*	• Digital *Smartphone app*	• Health professional performance on real-world data • Other *NHS111 data for 12 months from February 2017*	• General population *Participants in the London pilot evaluation of 'digital 111' services*
Berry 2016[14] USA	• Simulation *Evaluation of symptom checker performance on clinical vignettes*	• Online *17 symptom checkers*	• None	• Specific condition(s) *Gastrointestinal symptoms*
Berry 2017[32] USA	• Controlled observational	• Online *Three online symptom checkers (WebMD, iTriage and FreeMD)*	• Health professional performance on real-world data	• Specific condition(s) *Patients with a cough presenting to an internal medicine clinic*
Berry 2017[15] USA	• Controlled observational	• Online *Three online symptom checkers (WebMD, iTriage, FreeMD)*	• Health professional performance on real-world data	• Specific condition(s) *Abdominal pain*
Kellermann 2010 [11] USA	• Simulation *The developed algorithm was tested against past patient records..*	• Online *SORT was available on 2 interactive websites*	• Health professional performance on real-world data *The algorithm was tested against clinicians' decision on past patient records.*	• Specific condition(s) *Influenza symptoms*

For peer review only - http://bmjopen.bmj.com/site/about/guidelines.xhtml

Little 2016[12] UK	• Experimental *Randomised controlled trial (RCT)*	• Online *'Internet Doctor' website*	• Other *Usual GP care without access to the Internet Doctor website*	• Specific condition(s) *Respiratory infections and associated symptoms*
Luger et al. 2014 [33] USA	• Simulation *Described as "human-computer interaction study" using think-aloud protocols.*	• Online *Google and WebMD*	• Other *Comparing two internet health tools.*	• General population *Older adults (50 years or older)*
Marco-Ruiz et al. 2017 [34] Norway	• Qualitative *Qualitative element* • Other *1. Online evaluation by users (problem detection) 2. Think aloud technique by smaller sample of participants (usability)*	• Online *Erdusyk*	• None	• General population *Internet tool users*
Middleton 2016[16] UK	• Simulation	• Digital *'babylon check' automatic triage system*	• Health professional performance on test/simulation *Twelve 'clinicians' (doctors) and 17 nurses*	• General population
Nagykaldi 2010 [35] USA	• Uncontrolled observational	• Online *Customised practice website including a bilingual influenza self-triage module, a downloadable influenza toolkit and electronic messaging capability. A bilingual seasonal influenza telephone hotline was*	• None	• Specific condition(s) *Influenza*

For peer review only - http://bmjopen.bmj.com/site/about/guidelines.xhtml

		available as an alternative.		
Nijland 2016[19] Netherlands	• Uncontrolled observational *Retrospective analysis of 15 months' data*	• Online *Web-based triage system (http://www.dokterdokter.nl)*	• None	• General population
Poote 2014 [17] UK	• Uncontrolled observational	• Online *Prototype self-assessment triage system*	• Health professional performance on real-world data *GPs triage rating was compared with rating from the self-assessment system*	• General population *Students attending a University Student Health Centre with new acute symptoms*
Price 2013 [21] USA	• Uncontrolled observational	• Online *A web-based decision support tool - Strategy for Off-site Rapid Triage (SORT) for Kids designed to help parents and adult caregivers decide whether a child with possible influenza symptoms needs to visit the emergency department for immediate care.*	• Health professional performance on real-world data *The sensitivity of the algorithm was compared with a gold standard - evidence form child's medical records that they received 1 or more of 5 ED-specific interventions.*	• Specific condition(s) *Influenza in children*
Semigran 2015 [6] N/A	• Experimental *Described as an audit study*	• Multiple *23 symptom checkers were evaluated. Symptom checkers available as apps (via the App Store and Google Play) were identified through searching for "symptom checker" and "medical diagnosis" and screened the first 240 results. Symptom checkers available online were identified through searching Google and Google Scholar for "symptom checker"*	• Other *Vignettes had a diagnosis and triage attached to them and these were compared against the symptom checker advice.*	• General population *Where a single class of illness was examined by the symptom checker, the symptom checker was excluded from the study.*

For peer review only - http://bmjopen.bmj.com/site/about/guidelines.xhtml

		and "medical diagnosis" and screened the first 300 results.		
Semigran 2016 [20] USA	• Experimental *Comparison of physician and symptom checker diagnoses based on clinical vignettes*	• Multiple *"Human Dx is a web-and app based platform"*	• Health professional performance on test/simulation *Clincial vignettes - a comparison of 23 symptom checkers with physician diagnosis for 45 vignettes*	• General population *Of the 45 condition vignettes - there were 15 low, 15 medium and 15 huigh acuity vignettes - there were 26 common and 19 uncommon condition vignettes*
Sole 2006 [18] USA	• Uncontrolled observational *Descriptive comparative study*	• Online *A web-based triage system (24/7 WebMed)*	• Health professional performance on real-world data *Data was evaluated from students who had used the web based triage and then requested an appointment via email (so triage data was available for comparison).*	• General population
Yardley 2010[13] UK	• Experimental *Exploratory randomised trial*	• Online *'Internet Doctor' website*	• Other *Self-care information provided as a static web page with no symptom checker or triage advice*	• Specific condition(s) *Minor respiratory symptoms, e.g. cough, sore throat, fever, runny nose*

For peer review only - http://bmjopen.bmj.com/site/about/guidelines.xhtml

Table 2: Studies of symptom checkers as part of an electronic consultation system

Reference	Study design	System type	Comparator	Population/sample
Carter 2018 [23]<br>UK	• Uncontrolled observational<br>*Mixed-methods evaluation*	• Online<br>*webGP(subsequently known as eConsult)*	• Other<br>*Investigate patient experience by surveying patients who had used webGP and comparing their experience with controls (patients who had received a face-to-face consultation during the same time period) matched for age and gender*	• General population<br>*General practices in NHS Northern, Eastern and Western Devon Clinical Commissioning Group's area*
Cowie 2018[24]<br>UK	• Uncontrolled observational<br>*6-month evaluation at 11 GP practices in Scotland*	• Online<br>*eConsult, accessed via GP surgery websites. Service provides self-care assessment and advice, including symptom checkers; triage and signposting to alternative services; access to NHS24 (phone service); and e-consults allowing submission of details by e-mail)*	• None	• General population<br>*Patients registered with participating GP practices*
Madan 2014[25]<br>UK	• Uncontrolled observational<br>*Report of 6-month pilot study*	• Online<br>*webGP (subsequently known as eConsult)*	• None	• General population
NHS England[7]<br>UK	• Uncontrolled observational<br>*Analysis of data from four pilot studies together with data from other*	• Multiple<br>*Pilots featured NHS Pathways (Web-based; West Yorkshire); Sense.ly ('voice-activated avatar'; West Midlands); Espert 24 (Web-based; Suffolk) and babylon (app; North Central London)*	• None<br>*Authors stated it was not appropriate to compare pilot sites because of differences in starting date, 'footprints' covered, method of uptake and underlying population*	• General population

For peer review only - http://bmjopen.bmj.com/site/about/guidelines.xhtml

	sources			
Nijland 2009[26] Netherlands	• Other<br>*Online survey*	• Online<br>*Responses of interest relate to 'indirect e-consultation' (consulting a GP via secure e-mail with intervention of a Web-based triage system)*	• None	• General population<br>*Patients with Internet access but no experience of e-consultation*

For peer review only

Results by outcome

Safety

None of the six included studies that reported on safety outcomes identified any problems or differences in outcomes between symptom checkers and health professionals. Most of the studies compared system performance with that of health professionals using real or simulated data. The only study with no comparison group was the 6-month pilot study of webGP[25], which reported 'no major incidents'.

Limitations of the studies included not being based on real patient data[16]; covering only a limited range of conditions[11 21]; and sampling a young healthy population (students) not representative of the general population of users of the urgent care system[17]. Studies of e-consultation systems did not generally collect data on those respondents who decided not to seek an appointment, limiting their ability to assess any impact on safety for this group. Overall, the evidence should be interpreted cautiously as indicating no evidence of a detrimental impact on safety rather than evidence of no detrimental effect.

Clinical effectiveness

Only two studies reported on clinical effectiveness outcomes, making it difficult to draw any firm conclusions. In the study by Little et al., those who used the Internet Doctor website experienced longer illness duration and more days of illness rated moderately bad or worse than the usual care group[12] The pilot study of the webGP system[25] reported that several patients received advice to seek treatment for serious symptoms that might otherwise have been ignored. However, no details or quantitative data were provided.

Costs/cost-effectiveness

Two included studies provided limited data on possible cost savings. Based on 6 months of pilot data, Madan[25]estimated savings of £11,000 annually for an average general practice (6,500 patients) compared with current practice. The report also suggested a saving to commissioners equivalent to £414,000 annually for a CCG (Clinical Commissioning Group, responsible for specifying and purchasing most health services in the NHS in England) covering 250,000 patients. These savings were specifically related to self-reported diversion of patients from GP appointments to self-care and from urgent care to e-consultation. Using

For peer review only - http://bmjopen.bmj.com/site/about/guidelines.xhtml

similar methodology, the manufacturers of the 'babylon check' app claimed average savings of over £10/triage compared with NHS111 by telephone, based on a higher proportion of patients being recommended to self-care[22].

Diagnostic accuracy

Eight studies reported at least some data on the diagnostic accuracy of symptom checkers. In spite of the diverse methods and comparisons in the included studies, almost all agreed that the diagnostic accuracy of symptom checkers was poor in absolute terms (e.g. in evaluating 'vignettes' designed to test knowledge of specific conditions, where the correct diagnosis was already known by definition) or relative to that of health professionals. In the most comprehensive evaluation, Semigran et al. evaluated 23 symptom checkers across 770 standardised patient evaluations[6]. Overall the correct diagnosis was made in 34% of cases (95% CI 31%-37%), although performance varied widely between symptom checkers, high and low acuity conditions and common and rare conditions. When the same authors compared the 23 symptom checkers with physicians using 43 vignettes, physicians were more likely to list the correct diagnosis first (out of three differential diagnoses) (72.1% vs. 34% $p<0.001$) as well as among the top three diagnoses (84.3% vs. 51.2% $p<0.001$)[20].

The only exception to the rule was an evaluation carried out at a student health centre[18]. Using data from 59 participants who used the 24/7 WebMed system and who were subsequently treated at the health centre, the study found good agreement between chief complaint, 24/7 WebMed classification and provider diagnosis (kappa values 0f 0.89 to 0.94). This study differed from the others in using data from students rather than a general population sample. In addition, the students' complaints were generally common and uncomplicated, a scenario in which symptom checkers performed relatively well in the study by Semigran et al.[20].

Accuracy of disposition (triage and signposting to appropriate services)

Six included studies reported on this outcome, all except one of which[15] evaluated a 'general purpose' symptom checker. As with diagnostic accuracy, diverse methodologies and outcome measures were used.

For peer review only - http://bmjopen.bmj.com/site/about/guidelines.xhtml

The results overall presented a mixed picture but most studies indicated that symptom checkers were inferior and/or more cautious in their triage advice compared with doctors or other health professionals. In their review of 23 symptom checkers, Semigran et al. found that the systems provided appropriate triage advice in 57% (95% CI 52% to 61%) of cases[6]. Performance varied across the systems evaluated, correct triage ranging from 33% to 78%. The NHS England pilot evaluation of four systems[7] found that agreement with clinical experts varied from 30% to 95%, although the number of responses also varied, reducing the comparability of the results.

For abdominal pain, Berry et al. evaluated three symptom checkers and found that 33% of diagnoses were at the same level of urgency as physician diagnoses (emergency, non-emergency or self-care); 39% were diagnosed as more serious and 30% less serious than the physician's judgement[15]. A similar level of agreement between algorithm and clinician (39%) was reported by Poote et al.[17], while the system evaluated by Nijland et al. advised patients to visit a doctor in 85% of cases, even when the symptoms were appropriate for self-care[19].

The only studies to report clearly equal or superior accuracy of disposition using an automated system were the evaluations of Babylon check by the company that developed the system. Middleton et al.[16] reported that using patient vignettes, the app gave an accurate triage outcome in 88.2% of cases, compared with 75.5% for doctors and 73.5% for nurses (unaware of the 'correct' diagnosis for the vignettes). When vignettes were delivered by a medical professional rather than actors, the accuracy of Babylon check increased to over 90%. A later report looked at triage results obtained as part of the NHS England pilot evaluation, concluding that all of 74 referrals to urgent or emergency care were appropriate[22].

Impact on service use/diversion

Eight studies reported on this outcome, although one of them[11] merely stated that it was not possible to assess the effect of the intervention (a web-based influenza triage system) on patients' use of health services.

The pilot evaluation of the webGP system reported that 18% of users planned to book an appointment but chose not to do so[25] In addition, 14% of users reported that they would have

For peer review only - http://bmjopen.bmj.com/site/about/guidelines.xhtml

attended a walk-in centre or other urgent care service if they had not had access to the webGP system.

The NHS England pilot evaluation of four online/digital systems in different regions of England[7] compared the recommendations of the digital systems with those of the NHS111 telephone service over a similar time period (the first months of 2017). Compared with the telephone service, the online and digital services directed a slightly higher proportion of patients to self-care (18% vs. 14%) and a lower proportion to other primary care services such as GPs, dental and pharmacy (40 vs. 60%). The manufacturer's data on the 'babylon check' app collected as part of the NHS England evaluation indicated that patients were more likely to be triaged to self-care by the app compared with NHS111 by telephone (40 vs. 14%)[22]. This figure includes people who received information leaflets on self-care as well as those who were actively triaged. If the former group is excluded, the figures for the two services are similar (14% for NHS111 and 15.6% for 'babylon check'[22].

In their study of self-assessment for students attending a university health centre, Poote et al. found that the prototype system they studied was able to identify a proportion of cases that doctors considered appropriate for self-care, suggesting a potential to reduce service use[17]. Similarly, Little et al's RCT of a web-based symptom checker designed to support self-care for respiratory symptoms[12] reported that patients in the intervention group had fewer contacts with doctors than the usual care control group despite having a longer duration of illness and more days with relatively severe symptoms. This was balanced by an increase in contacts with the NHS Direct telephone service (which preceded NHS 111) and it should be noted that the system under evaluation recommended people needing treatment to contact NHS Direct rather than go directly to a doctor. Finally, a study of young adults (students) found that intention to seek treatment for a hypothetical illness was stronger when the diagnosis was made with the aid of WebMD or Google than with no electronic aid[30].

Patient compliance with triage advice

Only two of the included studies reported specifically on patients' compliance (or intention to comply) with advice received. The NHS England pilot evaluation in four regions asked participants in two of those regions (Suffolk and London) what they intended to do based on the advice received[7]. No quantitative data were provided but the report stated that in the

For peer review only - http://bmjopen.bmj.com/site/about/guidelines.xhtml

Suffolk pilot, 'overall users would have followed the advice given'. However, those who were recommended to call 999 or attend an ED were more likely to seek advice from primary care or self-management. Similarly, in the London region there was generally good agreement between advice and intended action but patients recommended to call 999 or go to an ED indicated that they would seek advice from a GP. In a study of a web-based triage system in the Netherlands, 192 patients were asked about their intention to comply immediately after receiving advice from the system[19]. Thirty-five patients responded to a follow-up survey on actual compliance, of whom 20 (57%) reported that they had followed the advice. Compliance was correlated with intention to comply, which in turn was correlated with the patient's attitude towards the advice received.

Equity and inclusion

Fourteen studies investigated the outcome of equity and inclusion or compared users and non-users. One study [12]reported that patients who were classed as less deprived were more likely to agree to use "Internet Doctor" than decline participation, although no relationship was found between deprivation and results in this study or between e-Consult use and deprivation in another study [24]. Association between e-consultation use and education levels was explored in a third study. Patients with low to medium levels of education tended to be motivated toward indirect e-consultation (which involves contact with a health professional via e-mail), mainly to reduce uncertainty [26]

Evidence from included studies suggests that users of e-consultation were more likely to be young[7 23-25], employed[19 23 25] and female[7 19 24 25]than non-users. One study also found a significantly larger use by white patients (78%) than other ethnicities[24].

Risk of bias assessment

We assessed risk of bias in the two included RCTs[12 13] using the Cochrane risk of bias tool. Thirteen studies[11 18 19 23 24 26-29 31 33-35] were assessed with the tool for cross-sectional and cohort studies and four (six publications[6 17 20 21 36 37]) with the modified QUADAS tool. Seven grey literature reports and conference abstracts were not formally assessed for risk of bias[7 14-16 25 30 32]. Identified limitations were extracted for all included studies.

For peer review only - http://bmjopen.bmj.com/site/about/guidelines.xhtml

Risk of bias results are presented in Appendix 2. With the possible exception of the two randomised trials, the included studies generally had at least a moderate risk of bias. However, the diverse designs and objectives of the studies made risk of bias difficult to assess in some cases with the available tools. Grey literature reports containing relevant data were included in the review but not formally assessed for risk of bias. Reports prepared by individuals with a commercial interest in a specific system and published without independent peer review[16 25] should be treated with particular caution because of possible conflicts of interest.

Overall strength of evidence assessment/evidence map

The overall strength of evidence for key outcomes is summarised in Table 3. We found relatively strong evidence that the diagnostic accuracy of digital and online symptom checkers tends to be lower than that of health professionals; and that patients who have used these systems generally show high levels of satisfaction (mainly in non-comparative studies). Areas where evidence is lacking or inconsistent include clinical and cost-effectiveness, accuracy of disposition to appropriate services and patient compliance with advice received. For safety, we found no evidence of an increased risk with digital/online systems but the available evidence was weak.

For peer review only

For peer review only - http://bmjopen.bmj.com/site/about/guidelines.xhtml

Table 3: Overall strength of evidence by outcome

Outcome	Relevant studies	Evidence statement	Strength of evidence	Comments
Safety	**=Kellermann 2010[11]**<br>**=Little 2016[12]**<br>**=Middleton 2016[16]**<br>**=Poote 2014[17]**<br>**=Price 2013[21]**<br>Madan 2014[25]	No evidence of a difference in risk between health professionals and symptom checkers	Weaker	Rating changed from stronger based on study numbers and design to weaker because of low numbers of adverse events reported
Clinical effectiveness	**-Little 2016[12]**<br>?Madan 2014[25]	Insufficient evidence to draw any firm conclusions	Very limited	
Costs/cost-effectiveness	+Babylon Health 2017[22]<br>+/-Cowie 2018[24]<br>+Madan 2014[25]	Insufficient evidence to draw any firm conclusions	Inconsistent	
Diagnostic accuracy	?Berry 2016[14]<br>**-Berry 2017[32]**<br>**-Berry 2017[15]**<br>**-Price 2013[21]**<br>?Semigran 2015[6]<br>**-Semigran 2016[20]**<br>**=Sole 2006[18]**	Symptom checkers appear inferior to health professionals in terms of diagnostic accuracy	Stronger	Mainly for specific conditions or pre-prepared vignettes
Disposition accuracy	=Babylon Health 2017[22]<br>**-Berry 2017[15]**<br>**=Middleton 2016[16]**<br>?Nijland 2010[19]<br>**-Poote 2014[17]**<br>+/-Semigran 2015[6]	Inconsistent findings on accuracy of disposition	Inconsistent	Performance variable between different systems

For peer review only - http://bmjopen.bmj.com/site/about/guidelines.xhtml

Outcome	Relevant studies	Evidence statement	Strength of evidence	Comments
	+/-**NHS England 2017**[7]			
Service use/diversion	**?Kellermann 2010**[11]<br>**+/-Little 2016**[12]<br>+/-**Poote 2014**[17]<br>?**Carter 2018**[23]<br>?Cowie 2018[24]<br>+Madan 2014[25]<br>+/- **NHS England 2017**[7]<br>+Babylon Health 2017[22]<br>-Luger 2011[33]	Inconsistent findings on effects on service use	Inconsistent	
Compliance	?Nijland[19]<br>?NHS England 2017[7]	No comparative data on compliance	Very limited	
Patient/carer satisfaction	?Nagykaldi 2010[35]<br>?Nijland[19]<br>?Price 2013[21]<br>+Yardley[13]<br>?Carter 2018[23]<br>?Cowie 2018[24]<br>?Madan 2014[25]<br>?NHS England 2017[7]<br>?Lanseng 2007[29]	Most studies report high rates of patient satisfaction with symptom checkers and e-consultation systems generally	Weaker	Few studies with comparator data

Controlled studies in bold; = means no significant difference in outcomes; + means better outcome with symptom checker; +/- varying results within study; ? results difficult to interpret in comparative terms

For peer review only - http://bmjopen.bmj.com/site/about/guidelines.xhtml

Discussion

Main findings

The literature search identified 29 publications describing 27 studies that met the inclusion criteria. The overall strength of the evidence base varied between outcomes (Table 3), but in absolute terms the evidence is weak, being based largely on observational studies. A substantial component of grey literature of uncertain quality complicates the interpretation of the evidence. Interpretation of the evidence should also take into account risks of bias in individual studies. In addition, one included study evaluated 23 symptom checkers and only the overall findings are summarised in this review[6].

We found little evidence to indicate whether or not digital and online symptom checkers are detrimental to patient safety. The studies that reported on the outcome were mostly short-term and involved relatively small samples and hence reported few or no adverse events. Some were limited to people with specific types of symptoms and others recruited from specific population groups not representative of typical users of urgent care services. This body of evidence should therefore be interpreted cautiously and not extrapolated to the possible impact of a nationally available digital urgent care service being used by millions of people annually.

The evidence on patient satisfaction with digital and online systems also had some limitations but these findings appear more likely to be generalisable. Study participants generally expressed high levels of satisfaction, albeit in uncontrolled studies. For example, in the NHS England pilot evaluation, 70–80% of users were satisfied with their experience at each of the pilot sites[7]. This evidence, together with the increasing reliance on digital technology in all areas of life, suggests that any national digital urgent care service may be popular and well-used, although different sections of the population may differ in their degree of engagement (see the discussion of equity and inclusion below).

Digital and online systems have yet to achieve a high level of accuracy in the diagnosis of specific conditions. This finding applies both to ‘general purpose’ symptom checkers and to those limited to particular conditions. Although the evidence was classified as relatively

For peer review only - http://bmjopen.bmj.com/site/about/guidelines.xhtml

strong, several caveats should be applied. Some of the included studies did not recruit representative populations and others were based on standardised vignettes rather than real-world data. In addition, studies that compared symptom checkers with health professionals tended to use the doctors' clinical diagnosis as the reference standard, which would bias the comparison in favour of the health professionals. Poor diagnostic accuracy could also have implications for patient safety, although the limited evidence on safety outcomes (small samples and small numbers of events) makes it difficult to draw any firm conclusions. If symptom checkers are generally risk averse, this could potentially mitigate any effects on safety.

Accuracy of signposting of patients to the most appropriate level of service is closely related to diagnostic accuracy, but results for this outcome were inconsistent between studies. In general, algorithm-based triage tended to be more risk-averse than that of health professionals, with 85% of respondents being advised to visit their doctor in one study[19]. While there is considerable uncertainty about the magnitude of the effect, a national digital urgent care service could result in considerable numbers of patients receiving inappropriate advice to visit the ED or request an urgent GP appointment. Middleton and colleagues[16] claimed that the 'babylon check' app had a high degree of triage accuracy for vignettes compared with health professionals, but this non-peer-reviewed report requires further validation.

We also found inconsistent evidence on effects on service use. There was some indication that symptom checkers can influence the pattern of service use but the magnitude and direction of the effect varied between studies. Patients' reactions to online triage advice and whether they follow the advice or seek further help or information would have implications for service use but we found limited evidence for this outcome. Preliminary findings from the NHS England evaluation suggest that patients may be more likely to seek further advice for more urgent conditions[7] but further confirmation is required.

Over half of the included studies considered equity and inclusion issues either directly or by comparing users and non-users of digital triage systems. Not surprisingly, studies revealed a clear consensus that younger and more highly educated people are more likely to use these services while older and less educated patients are more likely to prefer telephone or face-to-face contact. This could have implications for health equity if urgent care pathways prioritise

For peer review only - http://bmjopen.bmj.com/site/about/guidelines.xhtml

For peer review only

(or appear to prioritise) requests originating from digital sources. Problems have arisen in primary care because patients using e-consultation systems to request an appointment following online triage may be seen more quickly than those contacting the practice by telephone.

Strengths and limitations

This systematic review was undertaken on a short timescale using a relatively large team of experienced researchers, including both methodological and topic experts. We performed a rigorous search of the literature including reference checking and citation searching. Rather than a conventional highly sensitive search (which would have resulted in inefficiencies in the screening process), we combined an initial focused search with subsequent rounds of follow-up searching, including searches for named symptom checker systems. We assessed risk of bias in individual studies using a variety of appropriate checklists as well as summarising the overall strength of evidence for key outcomes (Table 3).

The heterogeneous and descriptive nature of the included studies meant that meta-analysis was not feasible for any of the outcomes of interest. Our narrative synthesis approach used a mixture of description and tabulation to summarise the evidence for each of the pre-specified outcomes of interest. This was a review of published (including non-peer-reviewed) literature and the coverage of systems is not exhaustive; for example, we did not extract data from websites. We also did not carry out any original analyses of raw data even where such data were available. The timing of the review meant that final results of NHS England's pilot evaluation were not available to us. We were able to make use of a draft report that was published online[7] but we acknowledge that the findings of the final evaluation report, when available, will supersede those of the 2017 draft.

The review inclusion criteria were relatively broad and findings from symptom checker systems for specific conditions may not be applicable to more general systems and vice versa. We have also included studies of symptom checkers as part of electronic consultation systems in general practice, which again represents a slightly different setting from a general 'digital 111' service, and this should be kept in mind when interpreting the results.

For peer review only - http://bmjopen.bmj.com/site/about/guidelines.xhtml

A systematic review in such a topical area of research will require regular updating to keep track of new studies. For example, Verzantvoort et al.[38] published a study of self-triage using a smartphone app for out-of-hours primary care in the Netherlands shortly after our literature searches were completed. The app was rated highly for clarity and patient satisfaction. Sensitivity and specificity (using nurse telephone triage as reference standard) were 84% and 74% respectively, although diagnostic accuracy was only evaluated in a sample of participants (126/4456). Inclusion of this study would not have affected the main conclusions of our review.

For peer review only

Implications for service delivery and research

The implications of this systematic review for service delivery should be considered in the context that a decision has already been taken to introduce a 'digital 111' service and the service became available across England by December 2018. Achieving a high level of diagnostic accuracy will be key to the success of a 'digital 111' service. Failure to provide an accurate diagnosis may result in outcomes including patient dissatisfaction and unwillingness to use the service again; increased use of other urgent and emergency care services; and possible risks to patient safety (although the cautious approach characteristic of most existing systems may help to mitigate this).

The studies included in the review suggest a high level of uncertainty about the impact of 'digital 111' on the urgent care system and the wider healthcare system. Some of these uncertainties can be addressed by research and data collection but the health service may need to respond to short-term increases (or decreases) in demand and/or shifts from one part of the system to another. This may increase pressure on the system, at least in the short-term. In the longer-term, if usage of the 111 telephone service decreases as planned, there may be opportunities to reconfigure the workforce to support the integrated urgent care agenda.

Based on the areas of limited evidence identified by the review, priorities for research (in addition to ongoing collection of data to monitor usage and safety of the 'digital 111' service) include studies to compare the performance of different systems directly; rigorous economic evaluations based on real-world data; research to investigate the pathways followed by patients using the service; evaluation of systems designed for childhood illnesses; and

For peer review only - http://bmjopen.bmj.com/site/about/guidelines.xhtml

investigation of the possible role of behaviour change theory in the development and implementation of symptom checkers. Qualitative research to investigate perceptions of symptom checkers and barriers to their use by people who are less familiar with digital technology would also be of value.

Ethical approval

Ethical approval was not required for this work

Funding

This report presents independent research funded by the National Institute for Health Research (NIHR) Health Services & Delivery Research Programme (project number HSDR16/47/17). The funding programme approved the review protocol but had no role in the collection, analysis and interpretation of the data, the writing of this paper or the decision to submit the paper for publication. The views and opinions expressed are those of the authors and do not necessarily reflect those of the NHS, the NIHR, NETSCC, the HS&DR programme or the Department of Health.

Data sharing

No new data have been created in the preparation of this report and therefore there is nothing available for access and further sharing. All queries should be submitted to the corresponding author.

References

1. NHS England. NHS 111 minimum data set 2018-19 2018 [Available from: https://www.england.nhs.uk/statistics/statistical-work-areas/nhs-111-minimum-data-set/statistical-work-areas-nhs-111-minimum-data-set-nhs-111-minimum-data-set-2018-19/ accessed 29 October 2018.
2. NHS England. Five year forward view. Leeds: NHS England, 2014.
3. NHS England. Next steps on the NHS Five Year Forward View. Leeds: NHS England, 2017.
4. NHS England. Sustainability and transformation partnerships [Available from: https://www.england.nhs.uk/integratedcare/stps/ accessed March 25 2019.

For peer review only - http://bmjopen.bmj.com/site/about/guidelines.xhtml

5. Turner J, O'Cathain A, Knowles E, et al. Impact of the urgent care telephone service NHS 111 pilot sites: a controlled before and after study. *BMJ Open* 2013;3(11):e003451. doi: 10.1136/bmjopen-2013-003451
6. Semigran HL, Linder JA, Gidengil C, et al. Evaluation of symptom checkers for self diagnosis and triage: audit study. *BMJ* 2015;351:h3480.
7. NHS England. NHS111 online evaluation. Leeds: NHS England, 2017.
8. Hoffmann TC, Glasziou PP, Boutron I, et al. Better reporting of interventions: template for intervention description and replication (TIDieR) checklist and guide. *BMJ* 2014;348:g1687. doi: 10.1136/bmj.g1687
9. Reitsma JB, Rutjes AWS, Whiting P, et al. Chapter 9: Assessing methodological quality. In: Deeks JJ, Bossuyt PM, Gatsonis C, eds. Cochrane Handbook for Systematic Reviews of Diagnostic Test Accuracy Version 100: The Cochrane Collaboration 2009.
10. Baxter S, Johnson M, Chambers D, et al. The effects of integrated care: a systematic review of UK and international evidence. *BMC Health Serv Res* 2018;18(1):350. doi: 10.1186/s12913-018-3161-3
11. Kellermann AL, Isakov AP, Parker R, et al. Web-based self-triage of influenza-like illness during the 2009 H1N1 influenza pandemic. *Annals of Emergency Medicine* 2010;56(3):288-94.e6.
12. Little P, Stuart B, Andreou P, et al. Primary care randomised controlled trial of a tailored interactive website for the self-management of respiratory infections (Internet Doctor).[Erratum appears in BMJ Open. 2017 Mar 21;7(3):e009769corr1; PMID: 28325861]. *BMJ Open* 2016;6(4):e009769.
13. Yardley L, Joseph J, Michie S, et al. Evaluation of a Web-based intervention providing tailored advice for self-management of minor respiratory symptoms: exploratory randomized controlled trial. *Journal of Medical Internet Research* 2010;12(4):e66.
14. Berry AC, Berry BB, Nakshabendi R, et al. Evaluation of Accuracy Between Online Symptom Checkers for Diagnosis of Gastrointestinal Symptoms from MKSAP Clinical Vignette Board Review Questions. *Gastroenterology* 2016;150(4):S849-S50. doi: 10.1016/s0016-5085(16)32869-4
15. Berry AC, Cash BD, Mulekar MS, et al. SYMPTOM CHECKERS VS. DOCTORS, THE ULTIMATE TEST: A PROSPECTIVE STUDY OF PATIENTS PRESENTING WITH ABDOMINAL PAIN. *Gastroenterology* 2017;152(5):S852-S53. doi: 10.1016/s0016-5085(17)32937-2
16. Middleton K, Butt M, Hammerla N, et al. Sorting out symptoms: design and evaluation of the 'babylon check' automated triage system. London: Babylon Health, 2016.
17. Poote AE, French DP, Dale J, et al. A study of automated self-assessment in a primary care student health centre setting. *Journal of Telemedicine & Telecare* 2014;20(3):123-7.
18. Sole ML, Stuart PL, Deichen M. Web-based triage in a college health setting. *Journal of American College Health* 2006;54(5):289-94.
19. Nijland N, Cranen K, Boer H, et al. Patient use and compliance with medical advice delivered by a web-based triage system in primary care. *Journal of Telemedicine and Telecare* 2010;16(1):8-11. doi: 10.1258/jtt.2009.001004
20. Semigran HL, Levine DM, Nundy S, et al. Comparison of Physician and Computer Diagnostic Accuracy. *JAMA Intern Med* 2016;176(12):1860-61. doi: 10.1001/jamainternmed.2016.6001
21. Price RA, Fagbuyi D, Harris R, et al. Feasibility of web-based self-triage by parents of children with influenza-like illness: A cautionary tale. *JAMA Pediatrics* 2013;167(2):112-18.
22. Babylon Health. NHS111 powered by babylon: outcomes evaluation. London: Babylon Health, 2017.
23. Carter M, Fletcher E, Sansom A, et al. Feasibility, acceptability and effectiveness of an online alternative to face-to-face consultation in general practice: a mixed-methods study of webGP in six Devon practices. *BMJ Open* 2018;8(2):e018688.
24. Cowie J, Calveley E, Bowers G, et al. Evaluation of a digital consultation and self-care advice tool in primary care: a multi-methods study. *International Journal of Environmental Research and Public Health* 2018;15(896)
25. Madan A. WebGP: the Virtual general practice. London: Hurley Group, 2014.

For peer review only - http://bmjopen.bmj.com/site/about/guidelines.xhtml

26. Nijland N, van Gemert-Pijnen J, Boer H, et al. Increasing the use of e-consultation in primary care: Results of an online survey among non-users of e-consultation. *International Journal of Medical Informatics* 2009;78(10):688-703. doi: 10.1016/j.ijmedinf.2009.06.002
27. Backman AS, Lagerlund M, Svensson T, et al. Use of healthcare information and advice among non-urgent patients visiting emergency department or primary care. *Emergency Medicine Journal* 2012;29(12):1004-06.
28. Joury AU, Alshathri M, Alkhunaizi M, et al. Internet Websites for Chest Pain Symptoms Demonstrate Highly Variable Content and Quality. *Academic Emergency Medicine* 2016;23(10):1146-52.
29. Lanseng EJ, Andreassen TW. Electronic healthcare: a study of people's readiness and attitude toward performing self-diagnosis. *International Journal of Service Industry Management* 2007;18(3-4):394-417. doi: 10.1108/09564230710778155
30. Luger TM, Suls J. Online health information and intentions to seek healthcare. *Psychosomatic Medicine* 2011;73 (3):A59.
31. North F, Varkey P, Laing B, et al. Are e-health web users looking for different symptom information than callers to triage centers? *Telemedicine Journal & E-Health* 2011;17(1):19-24.
32. Berry AC, Berry NA, Wang B, et al. Symptom checkers versus doctors: A prospective, head-to-head comparison for GERD vs. Non-GERD Cough. *American Journal of Gastroenterology* 2017;112 (Supplement 1):S190. doi: http://dx.doi.org/10.1038/ajg.2017.299
33. Luger TM, Houston TK, Suls J. Older adult experience of online diagnosis: results from a scenario-based think-aloud protocol. *Journal of Medical Internet Research* 2014;16(1):e16.
34. Marco-Ruiz L, Bones E, de la Asuncion E, et al. Combining multivariate statistics and the think-aloud protocol to assess Human-Computer Interaction barriers in symptom checkers. *Journal of Biomedical Informatics* 2017;74:104-22.
35. Nagykaldi Z, Calmbach W, Dealleaume L, et al. Facilitating patient self-management through telephony and web technologies in seasonal influenza. *Informatics in Primary Care* 2010;18(1):9-16.
36. Fraser HSF, Clamp S, Wilson CJ. Limitations of Study on Symptom Checkers. *JAMA Internal Medicine* 2017;177(5):740-41.
37. Mehrotra A, Semigran HL, Levine DM, et al. Limitations of Study on Symptom Checkers. *JAMA Internal Medicine* 2017;177(5):741-41.
38. Verzantvoort NCM, Teunis T, Verheij TJM, et al. Self-triage for acute primary care via a smartphone application: Practical, safe and efficient? *PLoS One* 2018;13(6):e0199284. doi: 10.1371/journal.pone.0199284 [published Online First: 2018/06/27]

.

For peer review only

For peer review only

For peer review only - http://bmjopen.bmj.com/site/about/guidelines.xhtml

PRISMA 2009 Flow Diagram

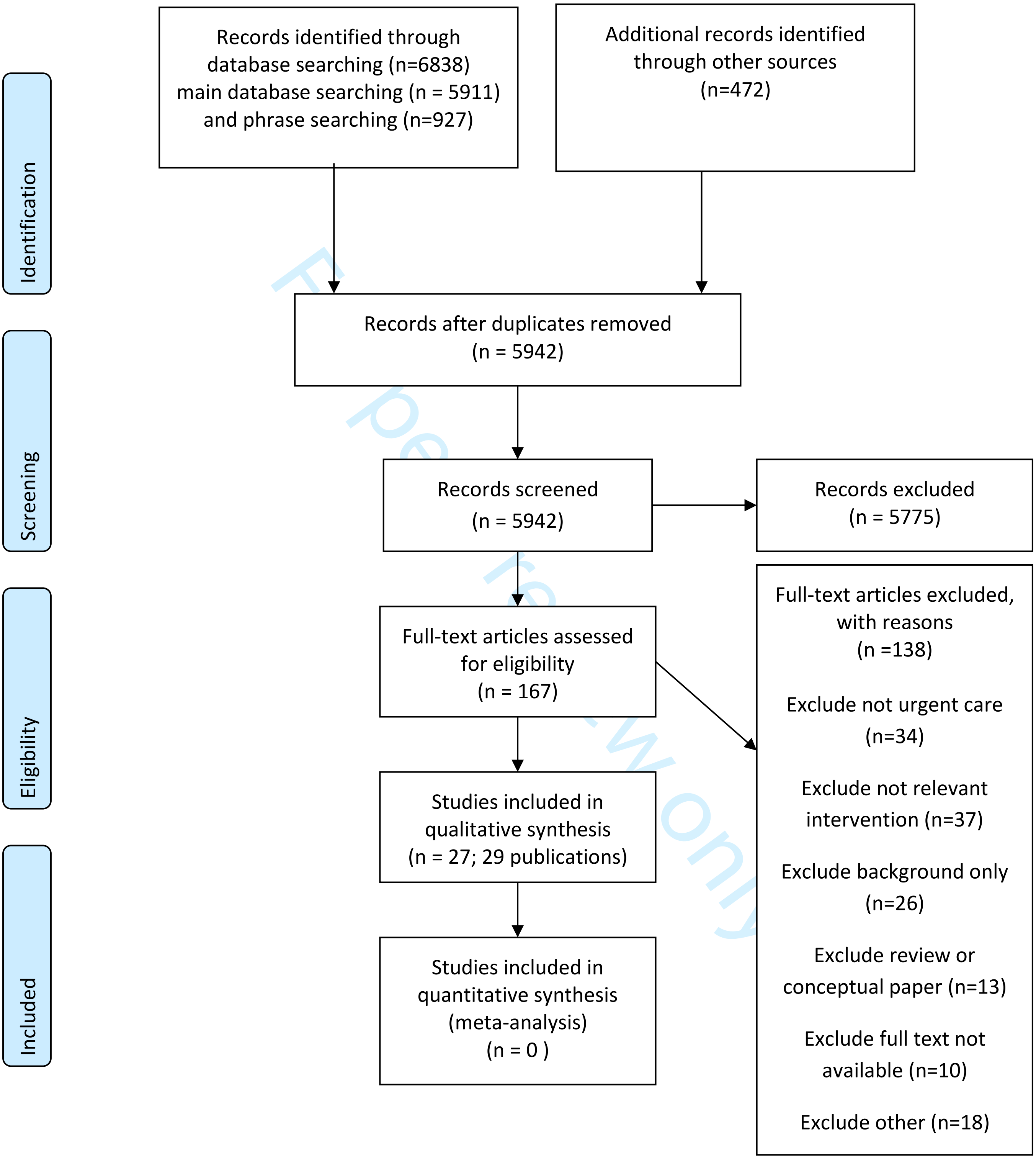

For peer review only

Appendix 1: Database search strategies

Database: Ovid MEDLINE(R) Epub Ahead of Print, In-Process & Other Non-Indexed Citations, Ovid MEDLINE(R) Daily and Ovid MEDLINE(R) <1946 to Present>
Search Strategy:
----
1 (symptom checker or symptoms checker or symptom checkers or symptoms checkers).tw. (21)
2 ("self diagnosis" or "self referral" or "self triage" or "self assessment").tw. (10438)
3 TRIAGE/ (10017)
4 2 or 3 (20415)
5 (online or on-line or web or electronic or automated or internet or digital or app or mobile or smartphone).tw. (658190)
6 4 and 5 (1568)
7 ("online diagnosis" or "web based triage" or "electronic triage" or etriage).tw. (42)
8 1 or 6 or 7 (1608)

****

For peer review only

Embase
----
1 (symptom checker or symptoms checker or symptom checkers or symptoms checkers).tw.
2 ("self diagnosis" or "self referral" or "self triage" or "self assessment").tw.
3 emergency health service/
4 2 or 3
5 (online or on-line or web or electronic or automated or internet or digital or app or mobile or smartphone).tw.
6 4 and 5
7 ("online diagnosis" or "web based triage" or "electronic triage" or etriage).mp. [mp=title, abstract, heading word, drug trade name, original title, device manufacturer, drug manufacturer, device trade name, keyword, floating subheading word, candidate term word]
8 1 or 6 or 7

Cochrane Library
#1 symptom checker or symptoms checker or symptom checkers or symptoms checkers:ti,ab,kw (Word variations have been searched)
#2 "self diagnosis" or "self referral" or "self triage" or "self assessment":ti,ab,kw (Word variations have been searched)
#3 MeSH descriptor: [Triage] explode all trees
#4 #2 or #3
#5 online or on-line or web or electronic or automated or internet or digital or app or mobile or smartphone:ti,ab,kw (Word variations have been searched)
#6 #4 and #5
#7 "online diagnosis" or "web based triage" or "electronic triage" or etriage:ti,ab,kw (Word variations have been searched)
#8 #1 or #6 or #7

CINAHL

S8 (S1 OR S6 OR S7)
S7 TI ("online diagnosis" or "web based triage" or "electronic triage" or etriage) OR AB ("online diagnosis" or "web based triage" or "electronic triage" or etriage)
S6 S4 AND S5

S5 TI (online or on-line or web or electronic or automated or internet or digital or app or mobile or smartphone) OR AB (online or on-line or web or electronic or automated or internet or digital or app or mobile or smartphone)
S4 (S2 OR S3)
S3 (MH "Triage")
S2 TI ("self diagnosis" or "self referral" or "self triage" or "self assessment") OR AB ("self diagnosis" or "self referral" or "self triage" or "self assessment")
S1 TI (symptom checker or symptoms checker or symptom checkers or symptoms checkers) OR AB (symptom checker or symptoms checker or symptom checkers or symptoms checkers)

ACM digital library

WOS
#8 #7 OR #6 OR #1
#7 TS=("online diagnosis" OR "web based triage" OR "electronic triage" OR etriage)
#6 #5 AND #4
#5 TS=(online OR on-line OR web OR electronic OR automated OR internet OR digital OR app OR mobile OR smartphone)
#4 #3 OR #2
#3 TS=triage
#2 TS=("self diagnosis" or "self referral" or "self triage" or "self assessment")
#1 (symptom checker or symptoms checker or symptom checkers or symptoms checkers)

HMIC

1 (symptom checker OR symptoms checker OR symptom checkers OR symptoms checkers).ti,ab
2 ("self diagnosis" OR "self referral" OR "self triage" OR "self assessment").ti,ab
3 TRIAGE/
4 (2 OR 3)
5 (online OR on-line OR web OR electronic OR automated OR internet OR digital OR app OR mobile OR smartphone).ti,ab
6 (4 AND 5)
7 ("online diagnosis" OR "web based triage" OR "electronic triage" OR etriage).ti,ab
8 (1 OR 6 OR 7)

For peer review only

Appendix 2: Risk of bias tables

Risk of bias results for randomised trials

Short Title	Reference	Selection and performance bias	Detection and attrition bias	Reporting and other bias
Little (2016)	**Study ID**<br>• Reference<br>*Little 2016*[12]	**Random sequence generation**<br>• Low risk<br><br>**Allocation concealment**<br>• Low risk<br><br>**Blinding of participants and personnel***<br>• Unclear	**Blinding of outcome assessment***<br>• Low risk<br>*Blinded assessment of primary care records*<br><br>**Incomplete outcome data***<br>• Low risk	**Selective reporting**<br>• Unclear<br><br>**Anything else, ideally prespecified**<br>• Low risk
Yardley (2010)	**Study ID**<br>• Reference<br>*Yardley 2010*[13]	**Random sequence generation**<br>• Low risk<br><br>**Allocation concealment**<br>• Low risk<br><br>**Blinding of participants and personnel***<br>• Low risk	**Blinding of outcome assessment***<br>• Unclear<br><br>**Incomplete outcome data***<br>• Low risk	**Selective reporting**<br>• Unclear<br><br>**Anything else, ideally prespecified**<br>• Low risk

For peer review only

Risk of bias results for cohort/cross-sectional studies

Reference	Questions 1-4	Questions 5-7	Questions 8-10
• Reference<br>*Backman A-S et al. 2012*[30]	**1. Was the research question clearly stated?**<br>• Yes<br>*The aims refer to "non-urgent" but the information is sought prior to visiting ED.*<br><br>**2. Was the study population clearly specified and defined?**<br>• Yes<br><br>**3. Was the participation rate at least 50%?**<br>• Yes<br>*79%*<br><br>**4. Were all the subjects selected or recruited from the same or similar populations?**<br>• Yes<br>*Primary care and ED attendees*	**5. Was a sample size justification provided?**<br>• No<br><br>**6. Did the study examine exposure levels?**<br>• Yes<br>*Health advice seeking*<br><br>**7. Were exposure measures clearly defined?**<br>• Unclear<br>*Measures are vague, e.g. "previous use" of information Also, discriminating between types of information*	**8. Were outcome measures clearly defined?**<br>• Unclear<br>*"Health care information use in the past"*<br><br>**9. Were outcome assessors blinded?**<br>• Not applicable<br><br>**10. Were confounders adjusted for?**<br>• Yes<br>*To some extent: participant and physician attributes assessed for influence on the results.*
• Reference<br>*Carter 2018* [26]	**1. Was the research question clearly stated?**<br>• Yes	**5. Was a sample size justification provided?**<br>• No	**8. Were outcome measures clearly defined?**<br>• Yes<br>*Attitudes and experiences of practice staff and*

For peer review only

	**2. Was the study population clearly specified and defined?**<br>• Yes<br>*GPs, practice staff and their patients at 6 practices in Devon*<br><br>**3. Was the participation rate at least 50%?**<br>• No<br>*Postal survey only had response rate of 35.1% but also GPs judgement of webGP requests and 5GPs and 5 administrators were interviewed.*<br><br>**4. Were all the subjects selected or recruited from the same or similar populations?**<br>• Yes<br>*GPs, practice staff and their patients at 6 practices in Devon*	**6. Did the study examine exposure levels?**<br>• Not applicable<br><br>**7. Were exposure measures clearly defined?**<br>• Not applicable	*patients on webGP.*<br><br>**9. Were outcome assessors blinded?**<br>• Not applicable<br><br>**10. Were confounders adjusted for?**<br>• Not applicable
• Reference *Cowie 2018*[27]	**1. Was the research question clearly stated?**<br>• Yes<br><br>**2. Was the study population clearly specified and defined?**<br>• Yes	**5. Was a sample size justification provided?**<br>• No<br><br>**6. Did the study examine exposure levels?**<br>• No	**8. Were outcome measures clearly defined?**<br>• Yes<br><br>**9. Were outcome assessors blinded?**<br>• No<br><br>**10. Were confounders adjusted for?**

For peer review only

	**3. Was the participation rate at least 50%?**<br>• No<br>*No for patient surveys*<br><br>**4. Were all the subjects selected or recruited from the same or similar populations?**<br>• Yes	**7. Were exposure measures clearly defined?**<br>• Not applicable	• Yes
• Reference *Joury et al. 2016 US*[31]	**1. Was the research question clearly stated?**<br>• Yes<br><br>**2. Was the study population clearly specified and defined?**<br>• Not applicable<br><br>**3. Was the participation rate at least 50%?**<br>• Not applicable<br><br>**4. Were all the subjects selected or recruited from the same or similar populations?**<br>• Not applicable	**5. Was a sample size justification provided?**<br>• No<br><br>**6. Did the study examine exposure levels?**<br>• Not applicable<br><br>**7. Were exposure measures clearly defined?**<br>• Not applicable	**8. Were outcome measures clearly defined?**<br>• Yes<br>*Scores used for readability, popularity, content and quality*<br><br>**9. Were outcome assessors blinded?**<br>• Not applicable<br><br>**10. Were confounders adjusted for?**<br>• Unclear
• Reference *Kellermann 2010* [11]	**1. Was the research question clearly stated?**<br>• Unclear<br><br>**2. Was the study population clearly specified and**	**5. Was a sample size justification provided?**<br>• Not applicable	**8. Were outcome measures clearly defined?**<br>• Not applicable<br><br>**9. Were outcome assessors blinded?**

For peer review only

	**defined?**<br>• Unclear<br>*Patients with influenza-like illness in US that accessed one of 2 websites http://www.flu.gov and www.H1N2 ResponseCenter.com*<br><br>**3. Was the participation rate at least 50%?**<br>• Not applicable<br><br>**4. Were all the subjects selected or recruited from the same or similar populations?**<br>• Unclear<br>*Only counted web hits, no demographic data available on patients. No data on usage of algorithm by clinicians or call centers.*	**6. Did the study examine exposure levels?**<br>• Not applicable<br><br>**7. Were exposure measures clearly defined?**<br>• Not applicable	• Not applicable<br><br>**10. Were confounders adjusted for?**<br>• Not applicable
• Reference<br>*Lanseng & Andreassen 2007 Norway*[32]	**1. Was the research question clearly stated?**<br>• Yes<br><br>**2. Was the study population clearly specified and defined?**<br>• Yes<br><br>**3. Was the participation rate at least 50%?**<br>• Unclear	**5. Was a sample size justification provided?**<br>• No<br><br>**6. Did the study examine exposure levels?**<br>• No<br>*Readiness*<br><br>**7. Were exposure**	**8. Were outcome measures clearly defined?**<br>• Yes<br>*Use of TRI*<br><br>**9. Were outcome assessors blinded?**<br>• No<br><br>**10. Were confounders adjusted for?**<br>• Unclear

	**4. Were all the subjects selected or recruited from the same or similar populations?**<br>• Yes	**measures clearly defined?**<br>• Not applicable	
• Reference *Luger et al. 2014*[23]	**1. Was the research question clearly stated?**<br>• Yes<br><br>**2. Was the study population clearly specified and defined?**<br>• Yes<br><br>**3. Was the participation rate at least 50%?**<br>• Unclear<br><br>**4. Were all the subjects selected or recruited from the same or similar populations?**<br>• Yes	**5. Was a sample size justification provided?**<br>• No<br><br>**6. Did the study examine exposure levels?**<br>• No<br><br>**7. Were exposure measures clearly defined?**<br>• Not applicable	**8. Were outcome measures clearly defined?**<br>• Yes<br><br>**9. Were outcome assessors blinded?**<br>• Not applicable<br><br>**10. Were confounders adjusted for?**<br>• Unclear
• Reference *Marco-Ruiz et al. 2017 Norway*[24]	**1. Was the research question clearly stated?**<br>• Yes<br><br>**2. Was the study population clearly specified and defined?**<br>• No<br><br>**3. Was the participation rate at least 50%?**	**5. Was a sample size justification provided?**<br>• No<br><br>**6. Did the study examine exposure levels?**<br>• No	**8. Were outcome measures clearly defined?**<br>• Not applicable<br><br>**9. Were outcome assessors blinded?**<br>• Not applicable<br><br>**10. Were confounders adjusted for?**<br>• Unclear

For peer review only

	• Yes<br>*53%*<br><br>**4. Were all the subjects selected or recruited from the same or similar populations?**<br>• Unclear	**7. Were exposure measures clearly defined?**<br>• Not applicable	
• Reference<br>*Nagykaldi 2010* [25]	**1. Was the research question clearly stated?**<br>• Yes<br><br>**2. Was the study population clearly specified and defined?**<br>• Yes<br>*Study population was patients from 12 primary care practices in US.*<br><br>**3. Was the participation rate at least 50%?**<br>• Not applicable<br><br>**4. Were all the subjects selected or recruited from the same or similar populations?**<br>• Yes<br>*All participants were patients from 12 primary care practices that accessed customised practice website or telephone helpline*	**5. Was a sample size justification provided?**<br>• Not applicable<br><br>**6. Did the study examine exposure levels?**<br>• Not applicable<br><br>**7. Were exposure measures clearly defined?**<br>• Not applicable	**8. Were outcome measures clearly defined?**<br>• Yes<br>*Web hits on customised practice website influenza self-management webpages. Downloads of self-management influeza toolkit. Completion of Iflueza self-triage module sessions. Volume of calls to telephone hotlines. Qualitative feedback from patients on satisfaction with and utility of self-management websites and telephone hotline. Qualitative feedback from clinicians around their involvement and their perceptionsof patient self-management techniques.*<br><br>**9. Were outcome assessors blinded?**<br>• Not applicable<br><br>**10. Were confounders adjusted for?**<br>• Not applicable

For peer review only

• Reference *Nijland 2009*[29]	**1. Was the research question clearly stated?**<br>• Yes<br><br>**2. Was the study population clearly specified and defined?**<br>• Yes<br><br>**3. Was the participation rate at least 50%?**<br>• Unclear<br><br>**4. Were all the subjects selected or recruited from the same or similar populations?**<br>• Yes	**5. Was a sample size justification provided?**<br>• No<br><br>**6. Did the study examine exposure levels?**<br>• Not applicable<br><br>**7. Were exposure measures clearly defined?**<br>• Not applicable	**8. Were outcome measures clearly defined?**<br>• Yes<br><br>**9. Were outcome assessors blinded?**<br>• No<br><br>**10. Were confounders adjusted for?**<br>• Yes<br>*Methods not very clearly reported but appears to be multiple regression*
• Reference *Nijland 2016*[19]	**1. Was the research question clearly stated?**<br>• Yes<br><br>**2. Was the study population clearly specified and defined?**<br>• Yes<br><br>**3. Was the participation rate at least 50%?**<br>• No<br>*Low participation rate in survey relative to users of triage system (though unclear how many were invited to participate)*	**5. Was a sample size justification provided?**<br>• No<br><br>**6. Did the study examine exposure levels?**<br>• Not applicable<br><br>**7. Were exposure measures clearly defined?**	**8. Were outcome measures clearly defined?**<br>• Yes<br><br>**9. Were outcome assessors blinded?**<br>• No<br><br>**10. Were confounders adjusted for?**<br>• Unclear

For peer review only

	**4. Were all the subjects selected or recruited from the same or similar populations?**<br>• Yes	• Not applicable	
• Reference<br>*North et. al. 2011*[34]	**1. Was the research question clearly stated?**<br>• Yes<br><br>**2. Was the study population clearly specified and defined?**<br>• Yes<br><br>**3. Was the participation rate at least 50%?**<br>• Not applicable<br><br>**4. Were all the subjects selected or recruited from the same or similar populations?**<br>• Not applicable	**5. Was a sample size justification provided?**<br>• Not applicable<br><br>**6. Did the study examine exposure levels?**<br>• Yes<br>*Self-exposure*<br><br>**7. Were exposure measures clearly defined?**<br>• Not applicable	**8. Were outcome measures clearly defined?**<br>• Yes<br><br>**9. Were outcome assessors blinded?**<br>• Not applicable<br><br>**10. Were confounders adjusted for?**<br>• Unclear<br>*Some discussion of potential confounders.*
• Reference<br>*Sole 2006*[18]	**1. Was the research question clearly stated?**<br>• Yes<br>*"The primary purpose of this study was to identify and describe the demographic profile of students who used the newly implemented Web-based triage system. A secondary purpose was to compare Web-based triage diagnoses to the diagnoses made in clinic for a subset*	**5. Was a sample size justification provided?**<br>• No<br><br>**6. Did the study examine exposure**	**8. Were outcome measures clearly defined?**<br>• Not applicable<br><br>**9. Were outcome assessors blinded?**<br>• Not applicable

For peer review only

	of students who requested appointments"<br><br>**2. Was the study population clearly specified and defined?**<br>• Yes<br>*Students who used the web based triage over a four month implementation period (1290 students). Then of those students, those who requested an appointment via email (143 students), then of those 59 who attended the health centre after requesting an email appointment.*<br><br>**3. Was the participation rate at least 50%?**<br>• Not applicable<br><br>**4. Were all the subjects selected or recruited from the same or similar populations?**<br>• Yes	**levels?**<br>• Yes<br><br>**7. Were exposure measures clearly defined?**<br>• Yes	**10. Were confounders adjusted for?**<br>• Not applicable

Risk of bias results for diagnostic studies

Reference	Questions 1 to 4	Questions 5 to 8	Questions 9 to 11
**Study ID**<br>•<br>Reference<br>*Poote*	**1. Representative spectrum?**<br>• No<br>*Study participants were all patients registered at a student health centre in England attending with new acute*	**5. Differential verification avoided?**<br>• Not applicable?	**9. Relevant clinical information?**<br>• Yes<br><br>**10. Were uninterpretable results reported?**

For peer review only

2014 [17]	*symptoms. If the self-assessment triage system was only for students to be representative the study population would have needed to include range of student health centres in different areas. If the system was for any UK general practices the study population would have needed to include patients of all ages, ethnicity, gender etc from a range GP practices in different areas.*<br><br>**2. Acceptable reference standard?**<br>• Yes<br><br>**3. Acceptable delay between tests?**<br>• Yes<br><br>**4. Partial verification avoided?**<br>• Yes<br>*All patients that completed self-triage also had a GP consultation where the GP rated the urgency of their consultation.*	**6. Was the reference standard independent of the index test?**<br>• Unclear<br>*Patients took the assessment from self-triage through to their GP consultation.*<br><br>**7. Index test results blinded?**<br>• No<br>*Patients took the assessment from self-triage through to their GP consultation.*<br><br>**8. Reference standard results blinded?**<br>• Yes	• Not applicable<br><br>**11. Were withdrawals from the study explained?**<br>• Yes
**Study ID**	**1. Representative spectrum?**	**5. Differential**	**9. Relevant clinical information?**

For peer review only

• Reference *Price 2013* [20]	• No *SORT was only trialled in 2 Emergency Departments in US, a larger range would be needed for a representative spectrum. Also, patients were from ED not home so potentially sicker patients in the sample.* <br>**2. Acceptable reference standard?** • Yes *Sensitivity of SORT for kids algorithm in identifying the need for ED care was based on an explicit gold standard: documented evidence that the child received 1 or more of 5 ED-specific interventions.* <br>**3. Acceptable delay between tests?** • Yes <br>**4. Partial verification avoided?** • Yes	**verification avoided?** • Not applicable? <br>**6. Was the reference standard independent of the index test?** • Yes <br>**7. Index test results blinded?** • Yes <br>**8. Reference standard results blinded?** • Yes	• Yes <br>**10. Were uninterpretable results reported?** • Not applicable <br>**11. Were withdrawals from the study explained?** • No
**Study ID** • Reference *Semigran 2015* [4]	**1. Representative spectrum?** • Unclear *There were 45 standardised patient vignettes which were divided into three levels of triage urgency and included more and less common conditions. It is not clear how closely this replicates the spectrum of conditions that people use symptom checkers for.*	**5. Differential verification avoided?** • Not applicable? <br>**6. Was the reference standard independent of the**	**9. Relevant clinical information?** • Yes *This is the clinical information that would be supplied by the patient which may or may not differ from the information given by the vignette.* [#548 Semigran 2015.pdf] Page 8: *ion of the true clinical accuracy of symptom checkers.33 Some standardized patient vignettes con- tained specifc clinical language (for*

For peer review only

	**2. Acceptable reference standard?**<br>• Yes<br>[#548 Semigran 2015.pdf] Page 2: *The source for each vignette also provided the associated correct diagnosis.*<br><br>**3. Acceptable delay between tests?**<br>• Not applicable<br><br>**4. Partial verification avoided?**<br>• Not applicable	**index test?**<br>• Yes<br><br>**7. Index test results blinded?**<br>• Yes<br><br>**8. Reference standard results blinded?**<br>• Yes	*example, mouth ulcers, tonsils with exudate), and actual patients with the same condition might struggle with the words to use to describe their symptoms or use diferent terms. Therefore, our analysis represents an indirect assess- ment of how well symptom checkers would perform with actual patients*<br><br>**10. Were uninterpretable results reported?**<br>• Yes<br>[#548 Semigran 2015.pdf] Page 3: *ns for diagnosis and triage was high (Cohen's κ 0.90). In some cases we could not evaluate a vignette because some symptom checkers focus only on children or on adults or the symptom checker did not list or ask for the key symp- tom in the vignette. To avoid penalizing these symptom checkers, we referred to standardized patient vignettes that successfully yielded an output as "standardized patient evaluations."*<br><br>**11. Were withdrawals from the study explained?**<br>• Not applicable
**Study ID**<br>•<br>Reference *Semigran 2016* [8]	**1. Representative spectrum?**<br>• Unclear<br>*There were 45 standardised patient vignettes which were divided into three levels of triage urgency and included more and less common conditions. It is not clear how closely this replicates the spectrum of conditions that people use symptom checkers for.*	**5. Differential verification avoided?**<br>• Not applicable?<br><br>**6. Was the**	**9. Relevant clinical information?**<br>• Yes<br>*The physicians and the symptom checkers used the same vignettes*<br><br>**10. Were uninterpretable results reported?**

For peer review only

	**2. Acceptable reference standard?**<br>• Yes<br><br>**3. Acceptable delay between tests?**<br>• Not applicable<br><br>**4. Partial verification avoided?**<br>• No<br>*There was a total of 234 physicians involved in the study and of the 45 vignettes, each was solved by at least 20 physicians but it is not clear why they chose the specific vignettes to solve.*	**reference standard independent of the index test?**<br>• Not applicable<br><br>**7. Index test results blinded?**<br>• Yes<br><br>**8. Reference standard results blinded?**<br>• Yes	• Not applicable<br><br>**11. Were withdrawals from the study explained?**<br>• No<br>*It is unclear why the physicians chose to solve the specific vignettes*

For peer review only

PRISMA 2009 Checklist

Section/topic	#	Checklist item	Reported on page #
**TITLE**			
Title	1	Identify the report as a systematic review, meta-analysis, or both.	1
**ABSTRACT**			
Structured summary	2	Provide a structured summary including, as applicable: background; objectives; data sources; study eligibility criteria, participants, and interventions; study appraisal and synthesis methods; results; limitations; conclusions and implications of key findings; systematic review registration number.	2-3
**INTRODUCTION**			
Rationale	3	Describe the rationale for the review in the context of what is already known.	4-5
Objectives	4	Provide an explicit statement of questions being addressed with reference to participants, interventions, comparisons, outcomes, and study design (PICOS).	5
**METHODS**			
Protocol and registration	5	Indicate if a review protocol exists, if and where it can be accessed (e.g., Web address), and, if available, provide registration information including registration number.	5
Eligibility criteria	6	Specify study characteristics (e.g., PICOS, length of follow-up) and report characteristics (e.g., years considered, language, publication status) used as criteria for eligibility, giving rationale.	6
Information sources	7	Describe all information sources (e.g., databases with dates of coverage, contact with study authors to identify additional studies) in the search and date last searched.	5-6
Search	8	Present full electronic search strategy for at least one database, including any limits used, such that it could be repeated.	Appendix 1
Study selection	9	State the process for selecting studies (i.e., screening, eligibility, included in systematic review, and, if applicable, included in the meta-analysis).	6
Data collection process	10	Describe method of data extraction from reports (e.g., piloted forms, independently, in duplicate) and any processes for obtaining and confirming data from investigators.	7
Data items	11	List and define all variables for which data were sought (e.g., PICOS, funding sources) and any assumptions and simplifications made.	7
Risk of bias in individual studies	12	Describe methods used for assessing risk of bias of individual studies (including specification of whether this was done at the study or outcome level), and how this information is to be used in any data synthesis.	7
Summary measures	13	State the principal summary measures (e.g., risk ratio, difference in means).	N/A
Synthesis of results	14	Describe the methods of handling data and combining results of studies, if done, including measures of consistency (e.g., I^2) for each meta-analysis.	N/A

For peer review only

PRISMA 2009 Checklist

Section/topic	#	Checklist item	Reported on page #
Risk of bias across studies	15	Specify any assessment of risk of bias that may affect the cumulative evidence (e.g., publication bias, selective reporting within studies).	N/A
Additional analyses	16	Describe methods of additional analyses (e.g., sensitivity or subgroup analyses, meta-regression), if done, indicating which were pre-specified.	7
**RESULTS**			
Study selection	17	Give numbers of studies screened, assessed for eligibility, and included in the review, with reasons for exclusions at each stage, ideally with a flow diagram.	9
Study characteristics	18	For each study, present characteristics for which data were extracted (e.g., study size, PICOS, follow-up period) and provide the citations.	10-16
Risk of bias within studies	19	Present data on risk of bias of each study and, if available, any outcome level assessment (see item 12).	Appendix 2
Results of individual studies	20	For all outcomes considered (benefits or harms), present, for each study: (a) simple summary data for each intervention group (b) effect estimates and confidence intervals, ideally with a forest plot.	17-22
Synthesis of results	21	Present results of each meta-analysis done, including confidence intervals and measures of consistency.	N/A
Risk of bias across studies	22	Present results of any assessment of risk of bias across studies (see Item 15).	N/A
Additional analysis	23	Give results of additional analyses, if done (e.g., sensitivity or subgroup analyses, meta-regression [see Item 16]).	22-23
**DISCUSSION**			
Summary of evidence	24	Summarize the main findings including the strength of evidence for each main outcome; consider their relevance to key groups (e.g., healthcare providers, users, and policy makers).	26-27
Limitations	25	Discuss limitations at study and outcome level (e.g., risk of bias), and at review-level (e.g., incomplete retrieval of identified research, reporting bias).	28
Conclusions	26	Provide a general interpretation of the results in the context of other evidence, and implications for future research.	28-29
**FUNDING**			
Funding	27	Describe sources of funding for the systematic review and other support (e.g., supply of data); role of funders for the systematic review.	29

From: Moher D, Liberati A, Tetzlaff J, Altman DG, The PRISMA Group (2009). Preferred Reporting Items for Systematic Reviews and Meta-Analyses: The PRISMA Statement. PLoS Med 6(7): e1000097. doi:10.1371/journal.pmed1000097

For more information, visit: **www.prisma-statement.org**.

For peer review only - http://bmjopen.bmj.com/site/about/guidelines.xhtml

The PRISMA for Abstracts Checklist

TITLE	CHECKLIST ITEM	REPORTED ON PAGE #
1. Title:	Identify the report as a systematic review, meta-analysis, or both.	1 (also in 'Design')
**BACKGROUND**		
2. Objectives:	The research question including components such as participants, interventions, comparators, and outcomes.	3 (Objectives)
**METHODS**		
3. Eligibility criteria:	Study and report characteristics used as criteria for inclusion.	3 (Eligibility criteria)
4. Information sources:	Key databases searched and search dates.	3 (Data sources)
5. Risk of bias:	Methods of assessing risk of bias.	3 (DE and synthesis)
**RESULTS**		
6. Included studies:	Number and type of included studies and participants and relevant characteristics of studies.	3 (Results)
7. Synthesis of results:	Results for main outcomes (benefits and harms), preferably indicating the number of studies and participants for each. If meta-analysis was done, include summary measures and confidence intervals.	3 (Results)
8. Description of the effect:	Direction of the effect (i.e. which group is favoured) and size of the effect in terms meaningful to clinicians and patients.	3 (Results)
**DISCUSSION**		
9. Strengths and Limitations of evidence:	Brief summary of strengths and limitations of evidence (e.g. inconsistency, imprecision, indirectness, or risk of bias, other supporting or conflicting evidence)	3 (Results)
10. Interpretation:	General interpretation of the results and important implications	3 (Conclusions)

**OTHER**		
11. Funding:	Primary source of funding for the review.	In text
12. Registration:	Registration number and registry name.	3

For peer review only
